# Cause or Effect of Arteriogenesis: Compositional Alterations of Microparticles from CAD Patients Undergoing External Counterpulsation Therapy

**DOI:** 10.1371/journal.pone.0046822

**Published:** 2012-10-08

**Authors:** Ali Al Kaabi, Tobias Traupe, Monika Stutz, Natasha Buchs, Manfred Heller

**Affiliations:** 1 Department of Clinical Research, University of Bern, Bern, Switzerland; 2 Department of Cardiology, University Hospital Bern, Bern, Switzerland; University of Cincinnati, United States of America

## Abstract

Recently, a clinical study on patients with stable coronary artery disease (CAD) showed that external counterpulsation therapy (ECP) at high (300 mmHg) but not at low inflation pressure (80 mmHg) promoted coronary collateral growth, most likely due to shear stress-induced arteriogenesis. The exact molecular mechanisms behind shear stress-induced arteriogenesis are still obscure. We therefore characterized plasma levels of circulating microparticles (MPs) from these CAD patients because of their ambivalent nature as a known cardiovascular risk factor and as a promoter of neovascularization in the case of platelet-derived MPs. MPs positive for Annexin V and CD31CD41 were increased, albeit statistically significant (*P*<0.05, vs. baseline) only in patients receiving high inflation pressure ECP as determined by flow cytometry. MPs positive for CD62E, CD146, and CD14 were unaffected. In high, but not in low, inflation pressure treatment, change of CD31CD41 was inversely correlated to the change in collateral flow index (CFI), a measure for collateral growth. MPs from the high inflation pressure group had a more sustained pro-angiogenic effect than the ones from the low inflation pressure group, with the exception of one patient showing also an increased CFI after treatment. A total of 1005 proteins were identified by a label-free proteomics approach from MPs of three patients of each group applying stringent acceptance criteria. Based on semi-quantitative protein abundance measurements, MPs after ECP therapy contained more cellular proteins and increased CD31, corroborating the increase in MPs. Furthermore, we show that MP-associated factors of the innate immune system were decreased, many membrane-associated signaling proteins, and the known arteriogenesis stimulating protein transforming growth factor beta-1 were increased after ECP therapy. In conclusion, our data show that ECP therapy increases platelet-derived MPs in patients with CAD and that the change in protein cargo of MPs is likely in favor of a pro angiogenic/arteriogenic property.

**Trial Registration:**

ClinicalTrials.gov NCT00414297

## Introduction

In patients with coronary artery disease (CAD), therapeutic promotion of coronary collateral growth is an attractive option for re-vascularizing myocardial tissue aside from percutaneous coronary intervention (PCI) and surgical bypass grafting. Collateral growth, or arteriogenesis, is triggered by increased tangential fluid shear stress at the endothelium and results in remodeling of pre-existing arteriolar anastomoses and improved blood flow [1]. Lower-leg, high-pressure external counterpulsation (ECP) triggered during diastole induces a flow velocity signal and thus, tangential endothelial shear stress in addition to the flow signal caused by cardiac stroke volume [2], [3].

During the last decades, ECP has been shown to be a safe and effective non-invasive therapy for patients with refractory angina pectoris [2], [3], [4], but the underlying mechanisms have not been clarified yet. Aside from increased collateral growth [5], [6], an improvement of myocardial perfusion [7], endothelial function, and/or nitric oxide bioavailability [5], [8], [9], [10] have been demonstrated after ECP treatment. In a recent study where chronic stable CAD patients were randomly assigned to low (80 mmHg) or high (300 mmHg) pressure ECP for a total of 30 hours, a significant increase of collateral function was demonstrated in the 300 mmHg group [5]. Similar results on collateral function have been obtained in an open, non-randomized trial of patients with chronic CAD undergoing 35 hours of ECP [6].

Microparticles (MPs) are shed from cells in response to diverse stimuli including injury, complement activation, apoptosis or high shear stress [11]. They are small, spherical vesicles ranging in size from 0.1–1.0 µm, and originate from endothelial cells, leukocyte, erythrocytes, and platelets. The main MP pool in human blood is platelet derived, abbreviated as PMP [12], [13]. MPs display cell surface proteins indicating their cellular origin, e.g. PMPs were found to be positive for CD31 (platelet endothelial cell adhesion molecule, PECA1) and CD41 (integrin alpha-IIb, ITA2B). In humans, plasma levels of circulating MPs are known to increase with the presence of cardiovascular risk factors and progression of atherosclerosis [14]. In contrast, PMPs have also been shown to promote angiogenesis *in vitro* through activation of endothelial cells indicating a potential beneficial effect [15], [16], [17]. Furthermore, PMPs may induce monocyte adhesion to endothelial cells by stimulating the proliferation, survival and adhesion of hematopoietic cells [18]. This in turn induces the release of growth factors and cytokines triggering inflammation-like processes [19]. Currently, there is no data available regarding these potential benefits of PMPs on arteriogenesis or collateral growth.

Given the above stated beneficial therapeutic effects of ECP in patients with CAD and the many properties related to MPs, we hypothesized that increased tangential fluid shear stress has an effect on circulating MPs *in vivo* and that these MPs are involved in ECP induced collateral growth. Retrospectively, we sought to determine plasma levels and the cell-specific origin of MPs obtained from CAD patients before and after treatment with either high or low inflation pressure ECP using flow cytometry. Furthermore, we studied the biological activity of these MPs with an *in vitro* angiogenesis assay and characterized the protein composition by a proteomics approach. Our results indicate to our knowledge for the first time, that ECP therapy alters the MP composition towards a pro-angiogenic/pro-arteriogenic property.

## Methods

### Study design

This investigation was part of a clinical trial evaluating the effect of ECP therapy on coronary collateral function (www.clinicaltrials.gov identifier NCT00414297) [5]. The independent institutional research ethics committee (Kantonale Ethikkommission Bern, Study No. KEK-BE 61/06) approved this study, and the patients gave written informed consent before participation. The study design has been described previously. Here, a subset of fifteen out of 20 patients from the former ECP study (age 63±10 years, 12 men) [5], for which blood plasma samples were available, were included. Patients had chronic stable one- (n = 1), two- (n = 8) or three-vessel (n = 6) CAD eligible for percutaneous coronary intervention (PCI) of at least one stenotic lesion. All patients underwent diagnostic coronary angiography because of symptoms related to CAD and were randomly assigned to lower-leg, high (300 mmHg, n = 7) or low inflation pressure (80 mmHg, n = 8) ECP treatment, that consisted of 20 sessions of 90 minutes duration each, given at five days per week during four weeks ( = 30 hours total).

If suitable and possible, pressure-derived collateral flow index measurements (CFI) [20], [21] were performed before and after ECP treatment in the coronary artery regarded to be the lesion responsible for the patient's symptoms (stenosed) and additionally in an angiographically and functionally normal coronary artery (normal vessel). In the 80 mmHg group, there were nine normal and seven stenosed vessel CFI values available, in the 300 mmHg group six normal and six stenosed. All patients taking medication at the start of the study continued with their medication throughout the study period.

### Collection of blood samples

At the end of the invasive procedure, arterial blood was collected from the right femoral artery using the 6F PCI guiding sheath in 9-mL EDTA tubes (Sarstedt, Germany) supplemented with protease inhibitor (Roche Applied Sciences, Switzerland). Blood samples were centrifuged within 15 minutes (2000× g for 20 min at 20°C) and plasma was collected by aspiration at 1 cm above the cell layer. Plasma was further centrifuged (16000× g for 2 min at 20°C) and aliquots of 250 µL or 500 µL were frozen and stored at −70°C for further analysis. Additionally, venous plasma samples were collected from healthy control subjects (n = 17).

### Characterization of microparticles by flow cytometry

Microparticles (MP) were isolated and incubated with antibodies as described elsewhere [22]. MP suspension aliquots of 5 µL, each, were separately diluted with 40 µL of a 1∶1 mixture of Tris/PBS buffer (50 mM Tris/HCl pH 7.2/10 mM PBS pH 7.2) and incubated at room temperature after the addition of 5 µL of the following monoclonal antibodies and their isotype negative controls: mouse anti-CD31 (PECAM-1, FITC, Sigma, Germany; isotype IgG1, Dako, Denmark), mouse anti-CD41 (platelet glycoprotein llb, RPE, Dako; isotype IgG1, Dako), mouse anti-CD62E (E-selectin, PE, Santa Cruz, U.S.A; isotype IgG1, Dako), mouse anti-CD146 (MCAM, PE, BD Biosciences, U.S.A; isotype IgG1, Dako), mouse anti-CD14 (monocyte, RPE, Dako; isotype IgG2a κ, Dako). For double staining, mouse anti-CD31 and mouse anti-CD41 were added together. Numbers of MPs were determined by Annexin V positive staining. Annexin binding buffer (10 mM HEPES, 150 mM NaCl, 5 mM KCl, 1 mM MgCl_2_ and 1.8 mM CaCl_2_, pH 7.4) was used for incubation with the Annexin V Cy5 (MBL international, Woborn, MA, U.S.A). Tris/PBS buffer served as Annexin negative control. After incubation, 900 µL Tris/PBS buffer was added and samples were analyzed with a LSR II flow cytometer with FACSDiva software (BD Biosciences, San Jose, California). In order to determine the flow rate of the flow cytometer, water flow rate was measured for 10 min. Taking dilution into account, numbers of MPs per mL plasma were calculated based on events per analyses period of 60 sec during a total analysis time of 70 sec. Data were evaluated by FlowJo software 7.5 (Tree Star, Inc. Oregon, U.S.A.). Assays were carried out with 5 replicates. Intra-assay and inter-assay reproducibility was evaluated in 10 independent experiments, showing an overall coefficient of variation of 13.0% and 10.3%, respectively (not shown).

### Angiogenesis Assay

Matrigel substrate (Millipore, Zug, Switzerland) was distributed in pre-cooled 96-well tissue culture plates according to the manufacturer's recommendations. MPs were isolated and suspended in 500 µL of EBM-2 medium (Lonza, Basel, Switzerland) and mixed with 3.5×10^4^ human umbilical vein endothelial cells (HUVEC). Aliquots of 150 µL of cell/MP suspension were distributed in three wells. EBM-2 and growth factor supplemented EGM-2 media served as controls. Development of tube formation was recorded with pictures taken of the same well area after 2, 4, 6, 8, 12, and 24 h using a Nikon microscope equipped with a charge-couple device camera. Each MP sample was measured in triplicate. The number of meshes and branching points were counted using ImageJ software. HUVECs were isolated from umbilical cords here at the local women's hospital (gift of D. Surbeck).

### MP analysis by mass spectrometry

MPs isolated as described above from 250 µL plasma were lysed in SDS-PAGE sample buffer and proteins separated on 10% SDS-PAGE gels. Gels were stained with Sypro Ruby**®** protein gel stain (Sigma, Buchs, Switzerland), images recorded followed by destaining of gels. Each sample lane was cut horizontally with a scalpel accurately into 14 slices per lane, and each slice was cut into small (∼1 mm3) cubes. Each slice was in-gel digested as described elsewhere [23]. Peptide sequencing was made on a LTQ Orbitrap XL mass spectrometer (ThermoFisher Scientific, Bremen; Germany) equipped with a Rheos Allegro nano flow system with AFM flow splitting (Flux Instruments, Reinach; Switzerland) and a nano electrospray ion source operated at a voltage of 1.7 kV. Peptide separation was performed on a Magic C18 column (5 µm, 100 Å, 0.075×70 mm) using a flow rate of ∼400 nL/min and a linear gradient of 5 to 40% acetonitrile in water/0.1% (v/v) formic acid during 60 min. Data acquisition was in data dependent mode on the top five ions with m/z between 360–1400. Precursor ions were excluded for 15 sec (±10 ppm). Survey full scan MS spectra were recorded in the orbitrap at resolution 60000 with automatic gain control (AGC) target of 5×10^5^ and ion time of 500 ms parallel to precursor ion fragmentation in the LTQ with the following settings: normalized collision energy of 30%, isolation width of 4.0 units, ion time of 200 ms, AGC target of 10^4^.

### Protein identification and quantification

Mascot generic files were generated by using Hardklör [24]. Database searches were done with Phenyx [25] against the forward and reversed UniprotKB SwissProt protein database (Release 2010_12) of human entries applying a two round search strategy. First round parameters were with a parent error tolerance of 20 ppm, normal cleavage mode with 1 missed cleavage and static carbamidomethylation on Cys and one variable oxidation on Met, respectively. During second round trypsin specificity was relaxed to half cleaved with 4 missed cleavages and variable amino acid modifications of carbamidomethylation on Cys, deamidation of Gln/Asn (maximum of 2 per peptide), oxidation of Met (2), and pyrrolidone carboxylic acid on n-terminal Asn (1), respectively. Based on reversed database peptide matches a z-score threshold of 1% peptide false discovery rate together with at least two unique peptide matches per protein were set as a protein acceptance criteria. Protein and peptide matches of each MP proteome were separately exported into MIAPE accepted excel format. Protein identifications from all samples were compared and annotated to a single consensus AC based on numbers of unique peptide matches. This filtering caused the loss of certain protein isoform identifications and some proteins remained with only one unique peptide match (Table S1). The peptide match score summation (PMSS) value for each identified protein was used as a semi-quantitative abundance estimate [23]. PMSS values were normalized by division with the median PMSS across all proteins in each sample and multiplication with the median across all samples. Repeatability of the applied proteomics approach was tested with three technical MP replicates of the same blood donor resulting in 175 proteins identified in all three runs. Normalized PMSS values correlated well between runs with linear correlations (R^2^) of 0.984, 0969, and 0.984 and linear regression slopes of 0.964, 0.984 and 1.02 respectively. The overall coefficient of variation was 26% (not shown). In this manuscript, we use the SwissProt ID as the protein name.

### Statistical Analyses

Data are given as mean ±1 standard deviation (SD). Baseline characteristics between the groups were analyzed by Mann-Whitney test for continuous data and by Fisher's exact test for categorical data. Two-tailed Wilcoxon signed rank tests were used for analytical results. A *P*-value<0.05 was considered as statistically significant. Matlab (R2010a) software was used.

## Results

### Patient characteristics and clinical data at baseline

Patient characteristics and clinical data at baseline are presented in Table 1. Patients presented without statistically significant differences between the high and low inflation pressure group regarding age, gender, duration of angina pectoris, history of myocardial infarction in a remote vascular area, and body mass index. No difference in the frequency of cardiovascular risk factors was observed except from family history for CAD (*P* = 0.04). Moreover, blood chemistry values including serum lipids did not differ at baseline between the groups (Table 1) nor between before (base-line, BL) and after (follow-up, Fup) ECP therapy (Table 2). The use of acetylsalicylic acid, clopidogrel, vasoactive drugs, statins or antihypertensive drugs did not differ significantly.

**Table 1:** Patient characteristics at start of therapy (baseline, BL) with n = 8 for ECP 80 mmHg and n = 7 for 300 mmHg group, respectively.

Variable	ECP 80 mmHg	ECP 300 mmHg	*P*
Age (years)	67±5	59±13	0.25
Male gender	6 (75%)	6 (86%)	1
Duration of angina (months)	9.5±17.2	8.3±9.6	0.39
History of prior myocardial infarction (%)	2 (25%)	2 (29%)	1
Body mass index (kg/m^2^)	28.1±6.3	28.1±3.9	0.56
**Cardiovascular risk factors**			
Systemic hypertension	5 (63%)	4 (57%)	1
Smoking	1 (13%)	1 (14%)	1
Cumulative pack years	15.0	15.7	0.77
Hypercholesterolemia	7 (88%)	6 (86%)	1
Family history for coronary artery disease	1 (13%)	5 (71%)	0.04
Obesity (BMI>30 kg/m^2^)	1 (13%)	3 (43%)	0.28
Diabetes mellitus	1 (13%)	2 (29%)	0.57
**Blood chemistry values**			
Total cholesterol (mmol/L)	4.6±1.3	4.2±1.0	0.49
Low density lipoprotein cholesterol (mmol/L)	2.9±1.0	2.5±0.8	0.18
High density lipoprotein cholesterol (mmol/L)	1.2±0.2	1.1±0.3	0.49
Triglycerides (mmol/L)	1.9±1.2	2.2±1.7	0.82
Creatinine (mmol/L)	81±15	71±15	0.15
Fasting glucose (mmol/L)	5.8±0.5	7.0±3.3	0.82
**Medication**			
Acetylsalicylic acid	8 (100%)	6 (86%)	0.47
Clopidogrel	4 (50%)	4 (57%)	0.62
Betablockers	4 (50%)	5 (71%)	0.61
Calcium channel blockers	0 (0%)	0 (0%)	-
Nitrates	1 (13%)	2 (29%)	0.57
Statins	8 (100%)	7 (100%)	1
ACE inhibitors	4 (50%)	3 (43%)	1
Angiotensin receptor blockers	3 (38%)	2 (29%)	1
Diuretics	2 (25%)	3 (43%)	0.61

**Table 2:** Plasma levels of blood cell counts, markers, and lipid levels.

	ECP 80 mmHg (n = 8)			ECP 300 mmHg (n = 7)		
*Cells* a	*BL*	*Fup*	*P* e	*BL*	*Fup*	*P* e
leukocytes	6.33±1.32	5.96±0.19	0.438	5.90±2.03	5.44±0.19	0.219
monocytes	0.47±0.19	0.39±0.18	0.063	0.44±0.19	0.35±0.20	0.313
Platelets	206±54	193±57	0.945	202±45	208±39	0.219
***Markers***						
CD146^b^	2.2±2.7	0.8±0.6	0.313	1.0±1.2	1.0±0.8	0.813
CD62E^b^	200±140	330±150	0.078	120±30	180±130	0.297
CD14^b^	7.0±4.1	5.5±4.5	0.461	9.2±6.3	9.5±6.0	0.938
hsCRP^c^	4.21±6.19	4.31±8.72	0.383	1.43±1.85	1.37±1.20	0.297
***Lipids*** d						
LDL	2.9±1.0	2.5±0.9	0.498	2.5±0.8	2.2±0.6	0.590
HDL	1.2±0.2	1.1±0.3	0.416	1.1±0.3	1.1±0.2	1.00
TG	1.9±1.2	1.7±1.3	0.141	2.2±1.7	1.8±1.0	0.563

a Cell mass is given as g/L, mean±SD.

b Cell markers are given as ×10^4^ counts/mL, mean±SD.

c hsCRP is given as mg/L, mean±SD.

d LDL and HDL associated cholesterol and total triglycerides (TG) are given as mmol/L, mean ± SD.

e *P* is the statistical probability that the measurement of BL are similar to Fup.

### High pressure ECP therapy improves collateral function in patients suffering from CAD

Collateral flow index increased significantly (*P*<0.05) from 0.141±0.060 to 0.201±0.067 after high pressure ECP as determined in twelve vessels, but not after low pressure ECP (from 0.127±0.091 to 0.137±0.063; sixteen vessels).

### Effect of ECP therapy on numbers and origin of MPs

**Figure 1:**
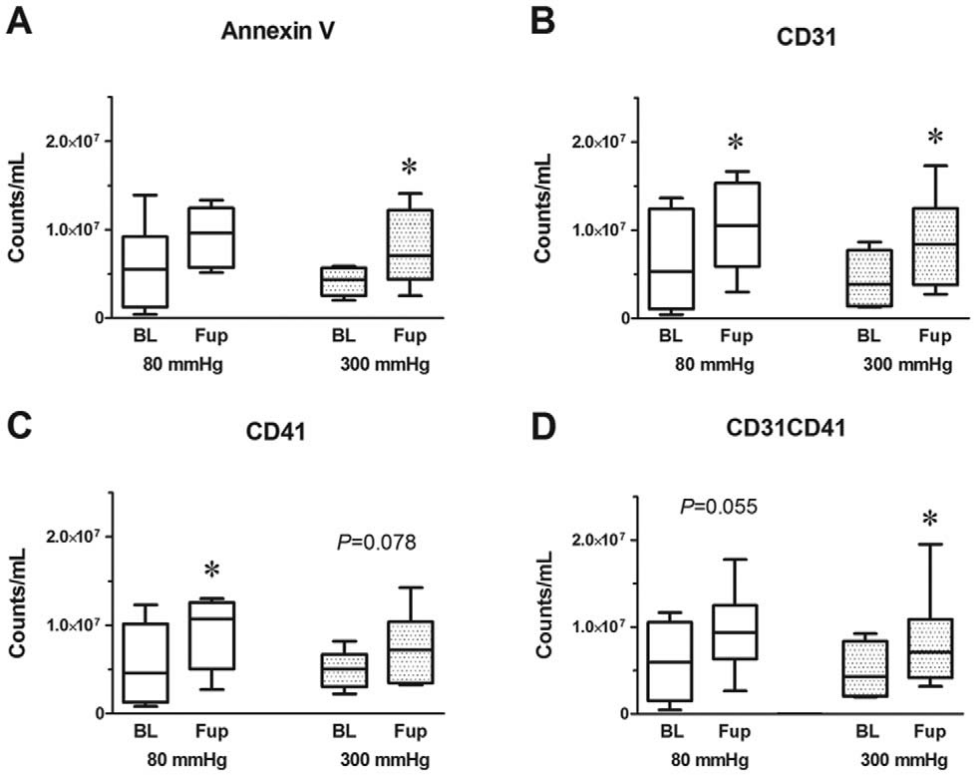
Characterization of circulating microparticles (MPs) by flow cytometry. MPs were isolated from plasma of CAD patients before (BL) and after (Fup) ECP therapy using low (80 mmHg) or high inflation pressure (300 mmHg). A, number of MPs determined by Annexin V. B and C, CD31 and CD41 positive MPs. D, PMPs double-stained for CD31 and CD41. Whiskers indicate minimum and maximum values. *P<0.05.

Annexin V positive MP counts were more than 3.4-fold higher (*P*<0.05) in this study population of CAD patients (5.1×10^6^±3.7×10^6^ counts/mL; n = 15) than in healthy controls (1.5×10^6^±1.0×10^6^ counts/mL; n = 17), not shown. High pressure ECP therapy in CAD patients increased numbers of Annexin V positive MPs significantly by 1.8-fold (*P*<0.05; n = 7), while low pressure ECP therapy resulted in no statistically significant effect (*P* = 0.148; n = 8) albeit with a 1.6-fold increase (Figure 1A).

MPs were stained for platelet endothelial cell adhesion molecule (CD31) and platelet membrane glycoprotein IIb (CD41). CD31 and CD41 positive MPs were both increased 1.6-fold in CAD patients treated by low pressure ECP (*P*<0.05), whereas only CD31 positive MPs increased significantly after high pressure ECP (1.95-fold; *P*<0.05), and the CD41+ increase did not reach statistical significance (1.5-fold; *P* = 0.078) (Figure 1B and C).

Platelet-derived MPs (PMPs), double stained for CD31 and CD41, increased significantly in CAD patients who underwent high pressure ECP (1.6-fold; *P*<0.05) and tended to increase after low inflation pressure therapy (1.8-fold; *P* = 0.055; Figure 1D). There was no statistically significant correlation between CD31 and CD41 PMPs and intact platelet numbers (Table 2). In addition, clopidogrel medication, an inhibitor of platelet activation, did not have any measurable effect on PMP or platelet numbers (not shown).

There were no statistically significant differences in lipid status or cell numbers of leukocytes, monocytes and platelets between baseline and follow-up, respectively. Furthermore, MPs derived from monocytes (stained for monocyte differentiation antigen CD14) and endothelial cells (stained with the cell surface glycoprotein MUC18, CD146, and the endothelial leukocyte adhesion molecule 1, CD62E or E-selectin) were not affected by ECP therapy in patients with CAD (Table 2).

### Correlation of changes between PMP and CFI

**Figure 2:**
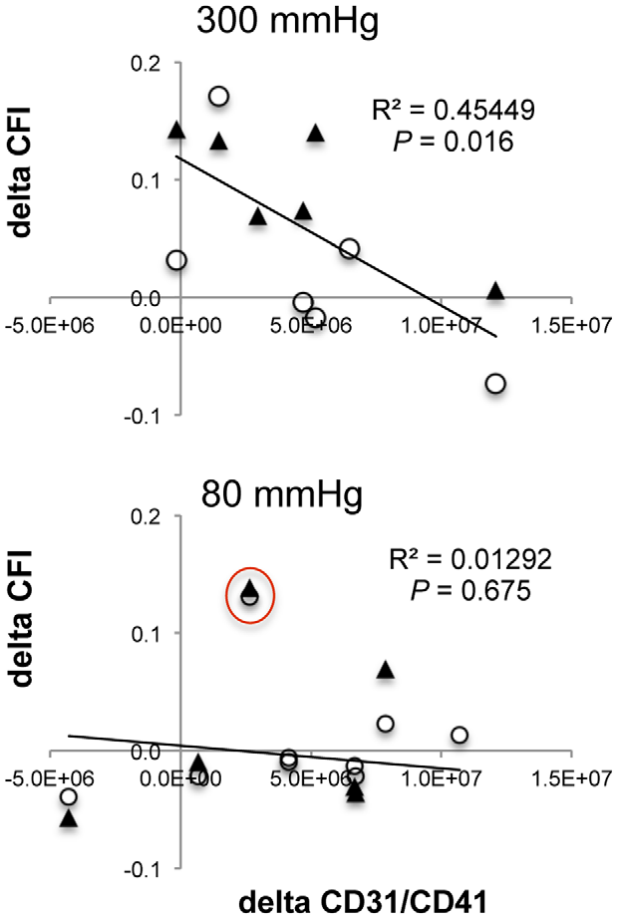
Correlation of PMP and CFI changes. Plotted values are the differences between follow-up and baseline measurements with platelet-derived microparticle positive for CD31CD41 on the horizontal axis and collateral flow index (CFI) on the vertical axis. Values are given for ECP therapy at high (300 mmHg) on top or low inflation pressure (80 mmHg) at bottom, respectively. Filled triangles represent CFI values from stenosed and open circles from normal vessels. The same correlations were detected with MPs stained positive with CD31 or CD41 alone (Figure S1). The values encircled in red in the 80 mmHg inflation pressure group belong to patient #14.

High, but not low, pressure ECP therapy of patients resulted in an inverse correlation of MP count changes (Fup minus BL values) positive for CD31, CD41, and CD31CD41, respectively, with the corresponding CFI changes (all *P*<0.05, Figure 2, Figure S1). The Pearson correlation coefficients did not differ significantly enough when considering all, or only stenosed or non-stenosed vessels alone (for instance for CD31CD41 double positive MPs with r_stenosed_ = −0.8173, r_non-stenosed_ = −0.695, and r_all_ = −0.6742). However, the CFI increased only in half of the non-stenosed vessels of the 300 mmHg inflation pressure group. This might indicate that a pro-arteriogenic effect of ECP therapy might be more efficient in occluded vessels. Unfortunately, there are not enough data points available for an unambiguous interpretation of this tendency.

One patient (#14) had a substantial increase in CFI after low inflation pressure treatment (marked with a red circle in Figure 2 and Figure S1). There were no significant correlations between changes of CD62E, CD146, CD14, or Annexin V counts and the corresponding CFI (data not shown).

### MPs of high inflation pressure group have increased pro-angiogenic properties

**Figure 3:**
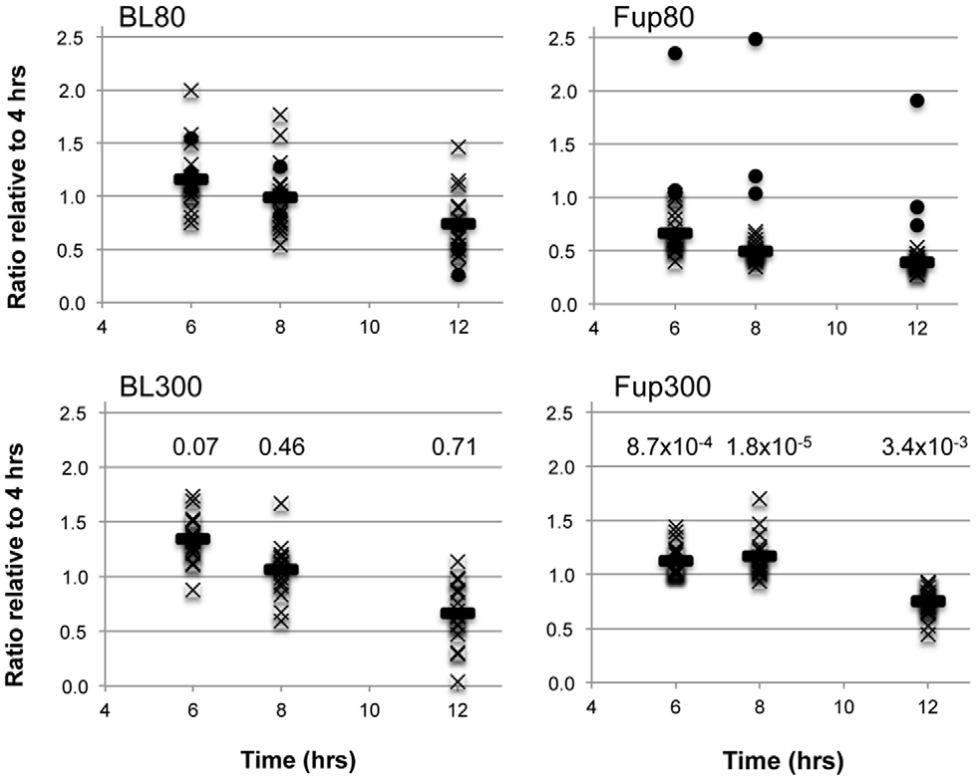
*In vitro* angiogenesis assay with isolated MPs. Circulating MPs were isolated from patient's plasma before (BL) and after (Fup) ECP therapy at 80 mmHg (top row) or 300 mmHg inflation pressure (bottom row). Displayed are numbers of meshes formed by HUVECs relative to the four-hour time point. The horizontal bars represent the mean value at each time point. The dots in the BL80 and Fup80 plots are the triplicate values measured for patient #14. The mean values between the 80 mmHg and 300 mmHg inflation pressure were tested for their likelihood to be the same by Student's t-test (two-tailed, equal variance) and *P* values at each time point are given in the bottom row graphs.

Angiogenesis assays with HUVECs using EBM2 and EGM2 medium resulted in fast tube formation within the first four hours of incubation quantified by mesh and branching point numbers. Mesh numbers decreased to 48.4±17.5% after 12 hours relative to the value at four hours (Figure S2 and S3). Tube formation was increased and sustained longer when MPs isolated from individual patient plasma samples at BL were added to EBM2 (at 8 hours, BL: 1.02±0.28, n = 34; medium alone: 0.72±0.22, n = 11; Students t-test: *P* = 0.0027). This effect was even more potent with MPs isolated from the high inflation pressure treatment group while it was reversed with the low inflation pressure therapy samples (see *P* values given in Figure 3). However, MPs of patient #14 treated with 80 mmHg ECP had a similar property as MPs of the 300 mmHg group (black dots in Figure 3).

### Qualitative MP protein composition assessment

MPs from three patients of each ECP treatment group were analyzed at baseline and follow-up by a proteomics approach. We identified a total of 1005 unique proteins, of which 123 were identified in all twelve samples and 470 proteins in at least half of them. This latter protein set was assigned ‘core proteins’. We compared our list of proteins with the ones published recently, namely circulating plasma MPs from 12 normal human subjects published by Østergaard *et al* [26], and from 42 CAD patients by Little *et al* [27], as well as PMPs from activated platelets by Garcia *et al* [28] and endothelial MPs derived from HUVEC by Peterson *et al* [29]. Our MP core protein set covered 83.6% of the Little core and 82.2% of the Østergaard core MP protein sets, respectively. The coverage was similar when considering all identified proteins, with 78.8% and 87.7% respectively (Figure S4). Our MP protein set covered similarly well the Garcia activated PMP protein set (76.8%) while the endothelial MP protein set of Peterson was covered only by 35.9% (Figure S4). In summary, 702 proteins ( = 69.9%) from our own complete MP set were confirmed by at least one of the other proteome studies (Table S1).

We performed gene ontology (GO) enrichment analyses with the core protein set using Cytoscape 2.8.2-beta2 and the BINGO 2.44 plugin by applying a hyper geometric test coupled with Benjamini & Hochberg false discovery rate correction. The highest significant enrichments for *cellular localization* (Table S2) were found in the GO terms of cytoplasmic part (including membrane-bounded vesicles and cytoskeleton), extracellular region, and platelet alpha granule; for *molecular function* (Table S3) in protein/actin binding, GTPase activity and GTP binding, and (endo-) peptidase inhibitor/regulator activity; for *biological processes* (Table S4) in response to wounding (hemostasis/coagulation), acute inflammatory response/complement activation/innate immune response, respectively.

### Quantitative MP protein composition assessment, core protein set

**Figure 4:**
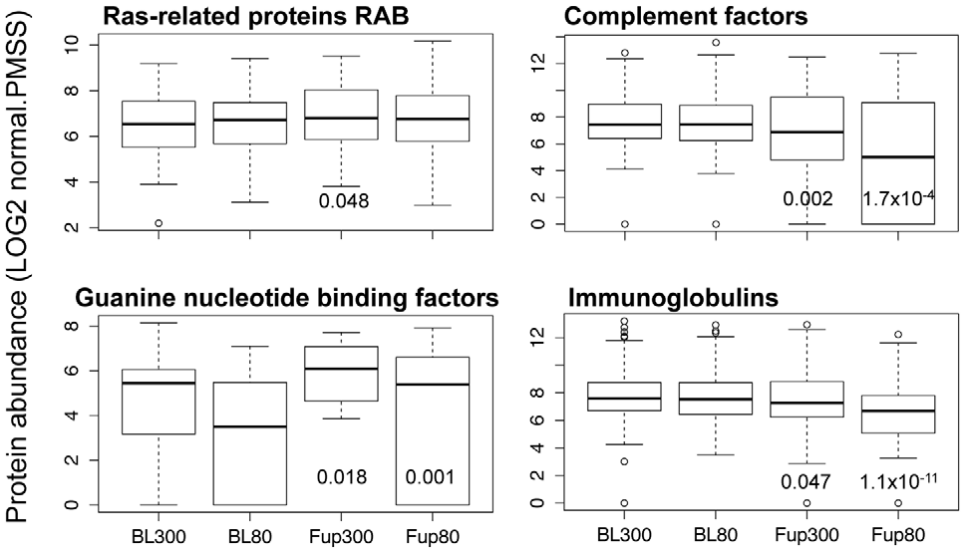
MP Protein quantification by label-free proteomics. Expression levels (LOG2 values of normalized protein abundance values) of cellular signaling proteins (left side) and proteins involved in the innate immune response (right side) are shown in boxplot format. RAS-related proteins RAB encompass RAB1B, RAB5C, RAB6B, RAB7A, RAB8A, RAB10, RAB11B, RAB15, RB27B, RAB35, RAP1B, and RAP2B, identified in all twelve analyzed MP samples. Guanine nucleotide binding factors encompass GNA13, GNAI2, GNAQ, GNAZ, GBB1, and GBB2, identified in at least eight MP samples. Complement factors considered were identified in at least eight MP samples: C1QA, C1QB, C1QC, C1R, C1S, CO2, CO3, CO4A, CO4B, CO5, CO6, CO7, CO8B, CO8G, CO9, and CFAB. All immunoglobulin heavy and light chains identified in at least 10 MP samples were considered. When one of the proteins was not identified its value was set at zero for statistical analysis of *P* values. *P* values are given below each box if the likelihood for a statistical difference between BL and Fup was at least 95%.

**Figure 5:**
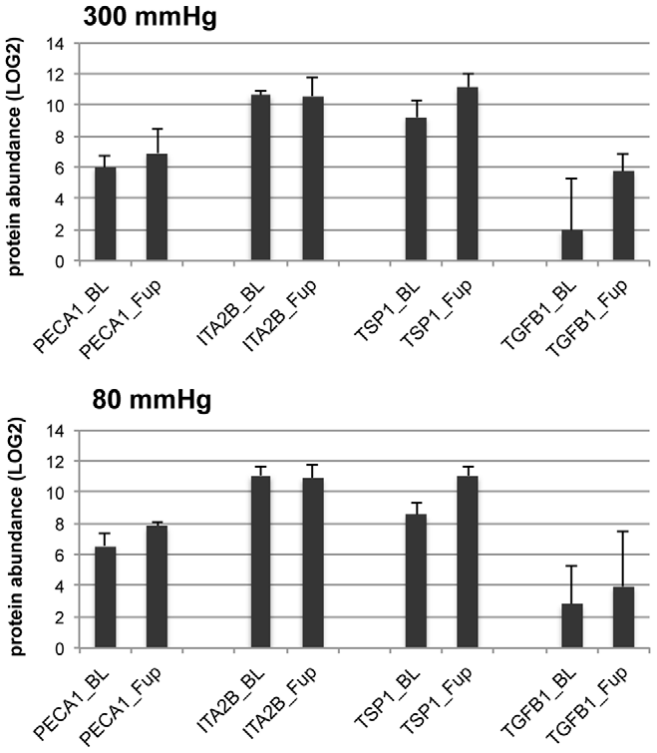
Label-free quantification of individual proteins. Bars represent mean abundance values (LOG2 values of normalized PMSS) of individual proteins before (BL) or after (Fup) ECP therapy with 300 mmHg (top) or 80 mmHg inflation pressure (bottom). Whiskers represent one standard deviation. Proteins displayed are PECA1 (CD31), ITA2B (CD41), TSP1 (thrombospondin-1), and TGFB1 (transforming growth factor beta-1).

**Figure 6:**
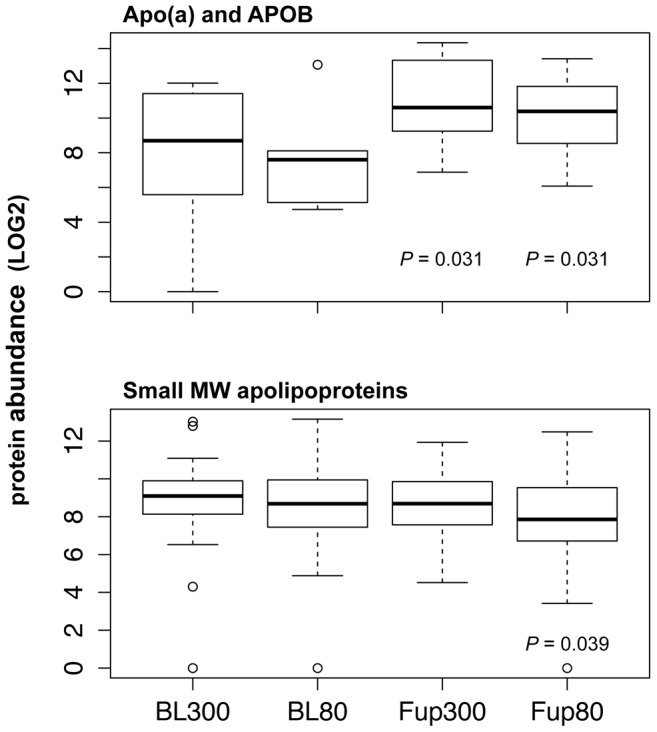
ECP therapy increases associations of Apo(a) and APOB to MPs. The sum of Apo(a) and APOB (top panel) was increased after low and high inflation pressure treatment of CAD patients. Small molecular weight apolipoproteins (APOA1, APOA2, APOA4, APOC1, APOC2, APOC3, APOD, APOE, and APOL1) were not changed or slightly decreased (bottom panel) in case of 80 mmHg inflation pressure treatment (*P* = 0.039), respectively.

Proteins involved in the biological process of inflammatory response/complement activation/innate immune response, such as immunoglobulins and complement factors, were statistically significant down regulated after high, and even more pronounced after low pressure ECP therapy (Figure 4). Certain classes of cellular proteins were up regulated after ECP therapy, e.g. myosins, tubulins, integrins and platelet markers, corroborating the increase in MP numbers as measured by flow cytometry (Figure S5). CD31 (PECA1) appeared to be increased after ECP therapy, also corroborating flow cytometry results, while CD41 (ITA2B) expression was unchanged (Figure 5). We also found that Ras-related proteins RAB and Guanine nucleotide binding factors, both classes of proteins involved in many intracellular signaling mechanisms, were up-regulate after ECP therapy (Figure 4). Two other proteins with many known effects on cellular homeostasis, angiogenesis and arteriogenesis, thrombospondin-1 (TSP1) and transforming growth factor beta-1 (TGFB1), were potentially increased in concentration after ECP therapy (Figure 5). Despite the fact that plasma lipid concentrations were not affected by ECP therapy (Table 2), we detected a statistically relevant increase (*P*<0.05) of Apo(a) and APOB while the group of small apolipoproteins remained unchanged, or decreased slightly after 80 mmHg treatment (Figure 6).

## Discussion

In our present study, we provide evidence that patients with stable CAD undergoing ECP therapy exhibit, on average, increased plasma levels of platelet-derived microparticles (PMPs) stained positive for CD31 and CD41 antigens. Importantly, the increase in MP numbers was not due to an increase of cells carrying CD31 antigen (platelets, leukocytes, and monocytes) or CD41 (platelets), and the increase of MPs was confirmed by semi-quantitative proteomics data revealing an increase in CD31 (PECA1) and cellular proteins. Furthermore, ECP did not induce monocyte (CD14) or endothelial cell (CD146 and CD62E) derived MPs. This was confirmed by a comparison of our MP proteome with the ones from MPs, PMPs and EMPs as recently published [26], [27], [28], [29]. We also demonstrate that patients with stable CAD have higher Annexin V positive MP counts than healthy controls, which is in agreement with previous studies [14]. Most interestingly, comparing changes between baseline and follow-up of individual patients, we found that changes in CFI inversely correlated with PMP count changes only in the high inflation pressure therapy group. However, one patient (#14) in the low inflation pressure group had a CFI increase concomitant with only a small increase in PMP numbers, a trait matching well with the high inflation pressure group characteristics. Furthermore, isolated MPs after ECP therapy from this patient had the same stimulating effect on endothelial cell tube formation in an *in-vitro* angiogenesis assay like the follow-up MPs isolated from the high inflation pressure group (Figure 3). This observation indicates that shear forces created with low inflation pressure therapy can already be sufficient for improved collateral vessel blood flow. The similarity of protein abundance patterns between high and low inflation pressure MP specimen, as discussed below, supports furthermore this finding.

The extent of collateral growth appears to be larger in response to ECP therapy than with other forms of physical [30] or pharmacological [31] coronary arteriogenesis. Regarding the cellular mechanisms of collateral growth, it is essential to distinguish arteriogenesis, defined as remodeling of collateral arterioles, independent of ischemia and mainly triggered by fluid shear stress, from angiogenesis, which is capillary sprouting initiated by tissue ischemia. Our findings point to a possible involvement of PMPs and their protein cargo in collateral growth, something not discussed so far in the existing literature. Additionally, the same PMPs also show a pro-angiogenic property in an *in vitro* angiogenesis assay. The effects of circulating MPs on angiogenesis described so far in the literature are abundant but inconsistent. It is well known that these effects depend on the origin of MPs. Lymphocyte- as well as endothelial-derived MPs were described as either pro- or anti-angiogenic [32], [33], [34], [35]. PMPs, which we found to be increased after ECP therapy, were previously reported to affect different stages of the angiogenic response, with a tendency towards a pro-angiogenic net effect even in the presence of angiogenesis inhibitors [36]. Furthermore, PMPs stimulated proliferation of cultured human endothelial cells in a dose-dependent manner and the phospholipids released from platelets induced endothelial cell migration and tube formation [15]. Additionally, stimulation of endothelial cells by PMPs *in vitro* resulted in cytokine release and expression of adhesion molecules [37].

One explanation for these observations might be that PMPs act as active transporters for different factors in the form of proteins, lipids and messenger RNA [15], [38], [39], [40]. Indeed, we identified with our proteomics approach after ECP therapy increased concentrations of platelet markers, integrins (Figure S5), intracellular signaling proteins (Figure 4), and growth factor modulating proteins TSP1 and TGFB1 (Figure 5). TGFB1 activates monocytes for binding to endothelial cells and it was found up-regulated at sites of growing collateral vessels in a rabbit hind-limb arteriogenesis model [41]. TSP1 is an extracellular matrix protein that binds many growth factors and acts as a pleiotropic growth regulator affecting morphogenesis and homeostasis of vessel cells [42]. We also detected an increase in Apo(a) and APOB, that together form at a 1∶1 ratio lipoprotein(a) particles. Our MP isolation protocol can potentially sediment lipoproteins together with MPs. Lipoprotein(a) particles have in average a lower density than high-density lipoprotein (HDL) particles that are composed of many small apolipoproteins and have a higher protein to lipid ratio. It has been reported that CD36 can interact with major lipoprotein classes, most likely through their lipid cargo as well as oxidized lipids carried by low-density lipoproteins [43]. CD36 was identified as a protein component of MPs with our proteomics approach (Figure S5). Although we do not have any direct evidence, our finding of an increase in Apo(a)/APOB concomitantly with unchanged small apolipoprotein concentrations in the MP fraction indicates that lipoprotein(a) might interact more strongly than HDL with PMPs possibly through CD36. Furthermore, we have recently shown that medium sized Apo(a) is a marker for good myocardial collaterization [44]. Whether Apo(a), or special lipids carried by it, is favorable for collateral growth still needs to be determined.

Another important class of proteins found associated with MPs and down-regulated after ECP therapy are complement factors and immunoglobulins. The involvement of the complement system in myocardial ischemia and reperfusion injury is well established [45]. Injury of vessel walls leads to the activation of the innate immune system. It has recently been shown that in the autoimmune disease systemic lupus erythematosus, MPs carry more complement and IgG proteins than controls [46]. Our findings confirm that these factors do associate with MP and that the MP load is decreased by ECP therapy. This again implies that vessel injury could be cured in CAD patients even with low inflation pressure, also called *sham* therapy (see above).

*In vitro*, it has been shown that PMPs promote adhesion and paracrine activity of angiogenic early outgrowth cells, potentially leading to improved endothelial restoration after vascular injury [47]. Most importantly however, an *in vivo* study demonstrated that locally injected PMPs are capable of increasing the number of capillaries after left coronary artery ligation (i.e. coronary angiogenesis) [16]. Taking these and our own experimental data and the difference between angiogenesis and arteriogenesis into account, it may be reasonable to speculate that PMPs might be involved in shear stress-induced effects of ECP directly at the endothelium or in regulating arteriogenesis, e.g. via cytokines, nitric oxide or delivery of activating cellular factors.

The main questions raised by our data are i) why an increase of PMPs was observed already with low pressure ECP, ii) why there was no pressure-dependent effect on the increase of PMPs, and iii) why we observed in patients receiving high pressure ECP therapy a significant inverse correlation of PMP changes with the corresponding changes of collateral flow index? First, the diastolic shear stress during 80 mmHg ECP therapy may be substantial and strong enough to cause shedding of platelets, and therefore release of PMPs. This notion is supported by a recently published study demonstrating that a short *ex vivo* application of low shear stress (10 dyne/cm^2^ for 3 minutes) already leads to significant formation of PMPs [48]. In comparison, it was shown during an experimental setting that high pressure ECP with sufficient diastolic augmentation (i.e. with a peak diastolic to a peak systolic amplitude ratio >1.2) increases diastolic shear stress to values greater than 50 dyne/cm^2^ [49]. Second, previous ECP studies with CAD patients repeatedly reported significant improvement of symptoms even in the absence of sufficient diastolic augmentation [2], which may not alone be due to a placebo effect but may also indicate neurohumoral effects even with *sham* therapy. Third, the changed protein composition of PMPs after four weeks of ECP therapy may point to a chaperon-like function of PMPs in arteriogenesis. The pressure-dependent diverging correlations between PMP and CFI changes during the four week therapy period could therefore be explained by a multi-factorial interrelationship between shear stress, inflammation, PMP protein cargo related stimulation of vessel cells, genetic disposition, and duration of treatment. Therefore, it can be speculated that higher shear stress, e.g. 300 mmHg ECP therapy, increases PMP production faster and enables activation of arteriogenesis at an earlier point in time during therapy than lower pressure ECP therapy, e.g. 80 mmHg. Hence, PMP concentration increases at the on-set of collateral growth followed by a decrease once collateral growth has reached a physiologically maximal level, determined by genetic predisposition. The data of patient #14 supports such a hypothesis. Forth, due to the small sample size, the presence or absence of statistical significance can be due to chance and needs further validation with more targeted approaches, e.g. more accurate protein quantification with LC-MS assays employing selected reaction monitoring assays on specific target proteins.

In conclusion, our data show that ECP therapy increases PMPs with a concomitant change of their protein composition in patients with CAD. PMPs are released even at low shear stress levels. Although, it is not absolutely clear if these PMPs are a cause or an effect of the CFI improvement, our data indicate that they might be players in a multifactorial process where inflammation-like processes are also involved. Further studies are necessary with blood sampling at shorter time intervals during therapy and more participating patients in order to elucidate the exact underlying cellular mechanisms and to improve the statistical power.

## Supporting Information

Figure S1 **Correlation of CD31 or CD41 positive MPs and CFI changes.** Correlation of MP changes (Fup minus BL) positive for CD31 (left side) or CD41 (right side) with corresponding changes of collateral flow index (CFI) are plotted. Values are given for ECP therapy at high (300 mmHg) on top or low inflation pressure (80 mmHg) at bottom, respectively. Filled triangles represent CFI values from stenosed and open circles from normal vessels. The values encircled red in the 80 mmHg inflation pressure group belong to patient #14.
(TIF)

Figure S2 ***In vitro*** **angiogenesis assay with HUVECs in Matrigel matrix.** HUVECs were seeded in EBM2 medium (left side) or in EBM2 medium supplemented with growth factors (EGM2, right side) and incubated for 24 hours. Pictures taken from the same area after 2, 4, 6, 8, and 12 hours of incubation at 37°C are shown.
(TIF)

Figure S3 **Quantitative evaluation of mesh formation by HUVECs in** ***in vitro*** **angiogenesis assay.** Ratios of intact mesh numbers after 6, 8, and 12 hours against the 4-hour values were calculated for each well. Crosses represent values with EBM2 (n = 5) and red circles with EGM2 (n = 6) medium. The horizontal bars represent the means of all measurements at each time point.
(TIF)

Figure S4 **Qualitative assessment of MP associated protein identifications with Venn diagrams.** Our own core and complete protein sets were compared with the Uniprot_SwissProt protein identifications of several MP proteome studies published. Østergaard and Little analyzed MPs isolated from human plasma. The complete SwissProt protein sets from endothelial derived MPs (EMP) and activated platelet derived MPs (PMP) from Peterson and Garcia, respectively, were also included in this comparison. Results demonstrate clearly that our MP associated proteins corresponded well with the ones described from other MP and PMP studies, but were less congruent with endothelial cell derived MPs.
(TIF)

Figure S5 **Label-free quantification of protein classes.** Boxplot representations of normalized protein abundance values (LOG2 values) of structural cell proteins (top panels) and cell surface proteins (bottom panels). The group of myosin proteins encompasses MYL6, MYH9, MYH10, MYH13, MYH14, MYL9, and ML12A that were identified in 12, 12, 6, 7, 10, 9, and 12 out of the 12 analyzed MP samples, respectively. Tubulins were TBA1A, TBA1B, TBA4A, TBA4B, TBB1, TBB4A, TBB4B, TBB5, TBB6, and TBB8, identified in at least eight MP samples. Platelet markers CD36, CXCL7, ITA2B, PECA1, PLF4 were identified in at least 12 MP samples, except LYAM3 with only seven. Integrins were ITA2, ITA2B, ITA6, ITB1, ITB3, and identified in at least 8 MP samples. When one of the proteins was not identified, its value was set at zero for statistical analysis of *P* values. *P* values are given above each box if the likelihood for a statistical difference between BL and Fup was close or better to 95%.
(TIF)

Table S1 **List of all identified proteins.**
(PDF)

Table S2 **Cellular component gene ontology enrichment analysis result with BINGO for identified core proteins.**
(TXT)

Table S3 **Molecular function gene ontology enrichment analysis result with BINGO for identified core proteins.**
(TXT)

Table S4 **Biological process gene ontology enrichment analysis result with BINGO for identified core proteins.**
(TXT)
