# Supplementary figures and images for: Cause or Effect of Arteriogenesis: Compositional Alterations of Microparticles from CAD Patients Undergoing External Counterpulsation Therapy
Source: PLoS One. 2012 Oct 8;7(10):e46822. doi: 10.1371/journal.pone.0046822 (PMC3466210; doi:10.1371/journal.pone.0046822)

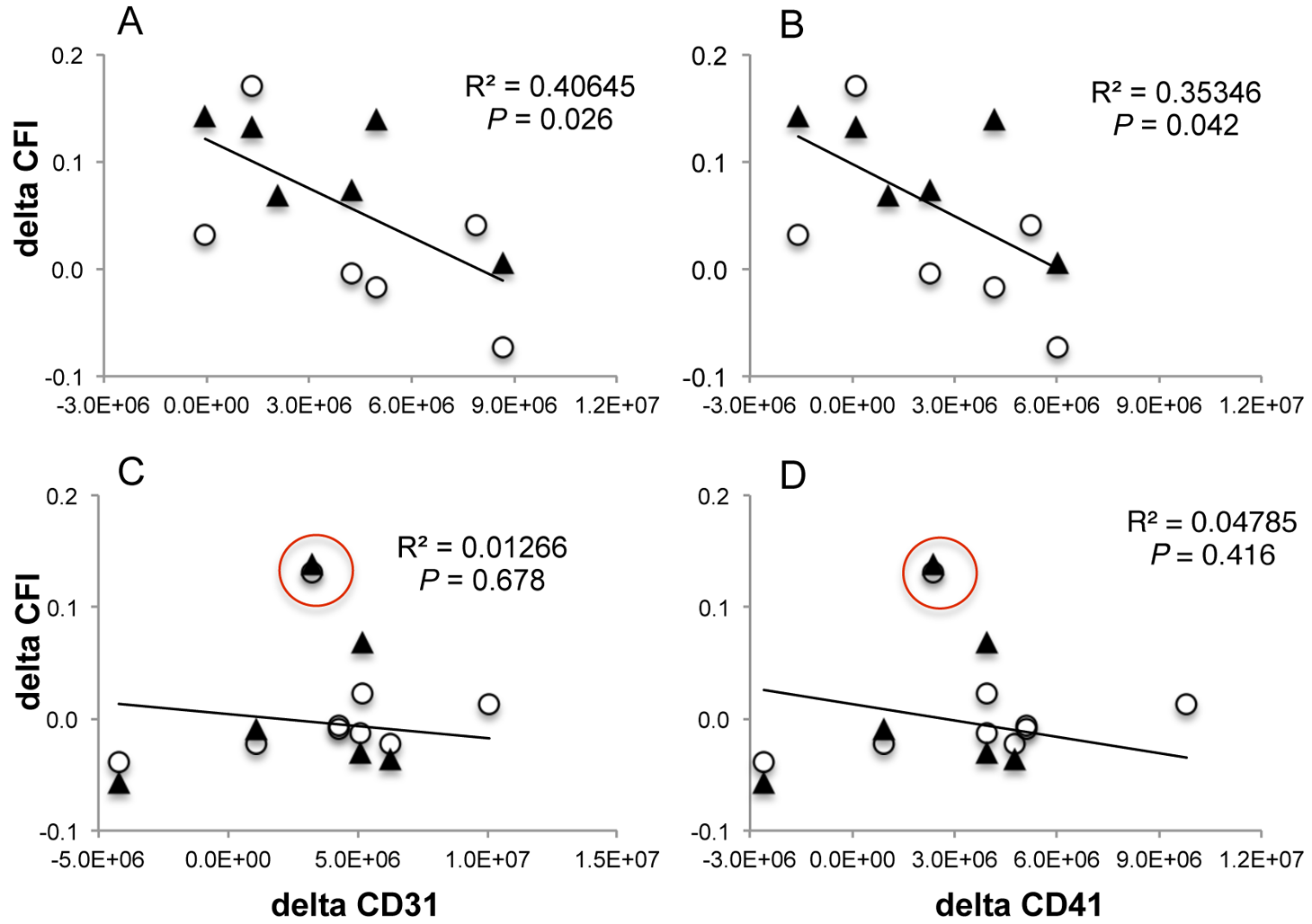

Supplement: Figure S1 — Correlation of CD31 or CD41 positive MPs and CFI changes. Correlation of MP changes (Fup minus BL) positive for CD31 (left side) or CD41 (right side) with corresponding changes of collateral flow index (CFI) are plotted. Values are given for ECP therapy at high (300 mmHg) on top or low inflation pressure (80 mmHg) at bottom, respectively. Filled triangles represent CFI values from stenosed and open circles from normal vessels. The values encircled red in the 80 mmHg inflation pressure group belong to patient #14. (TIF) [file pone.0046822.s001.tif]

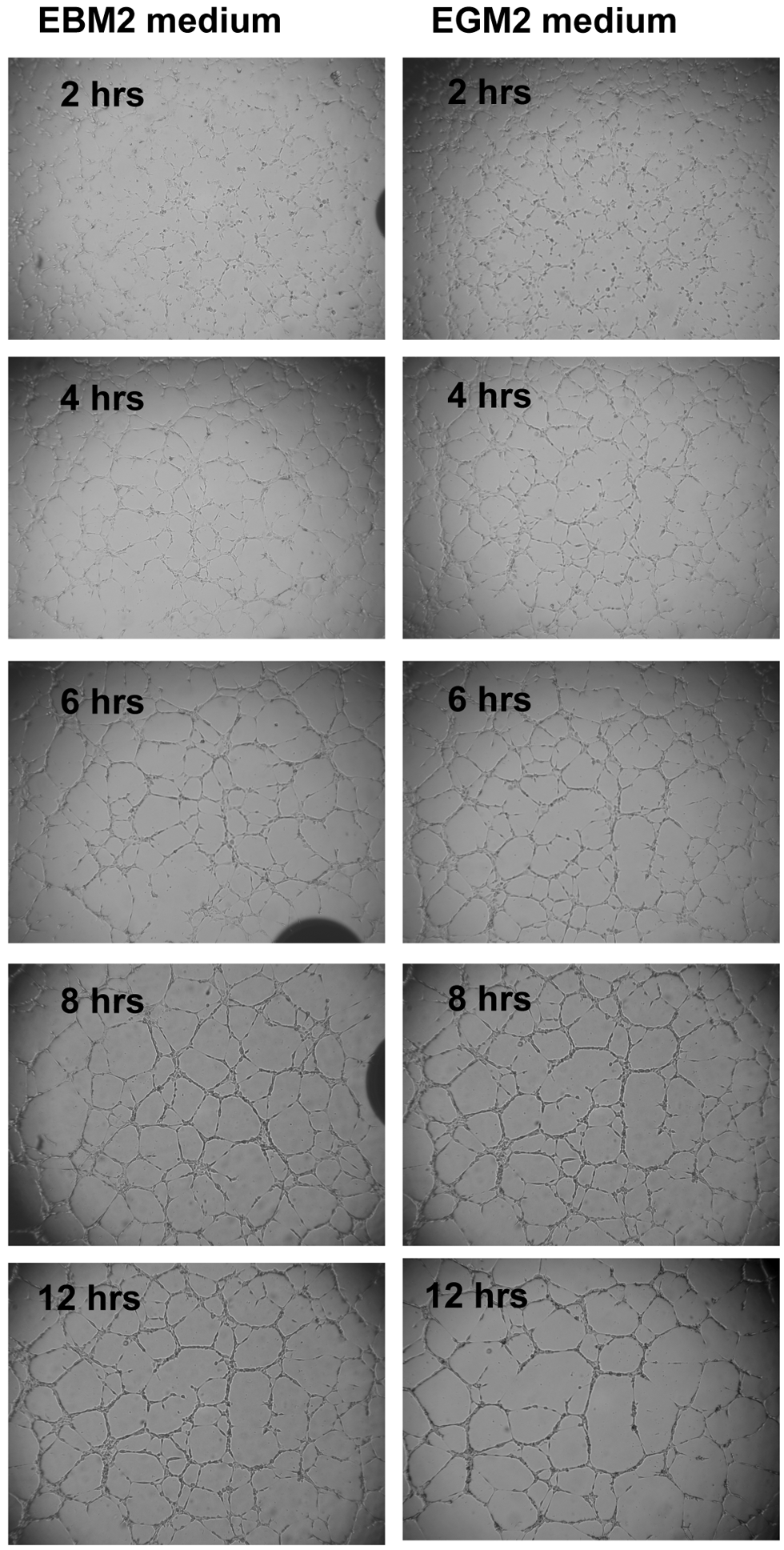

Supplement: Figure S2 — In vitro angiogenesis assay with HUVECs in Matrigel matrix. HUVECs were seeded in EBM2 medium (left side) or in EBM2 medium supplemented with growth factors (EGM2, right side) and incubated for 24 hours. Pictures taken from the same area after 2, 4, 6, 8, and 12 hours of incubation at 37°C are shown. (TIF) [file pone.0046822.s002.tif]

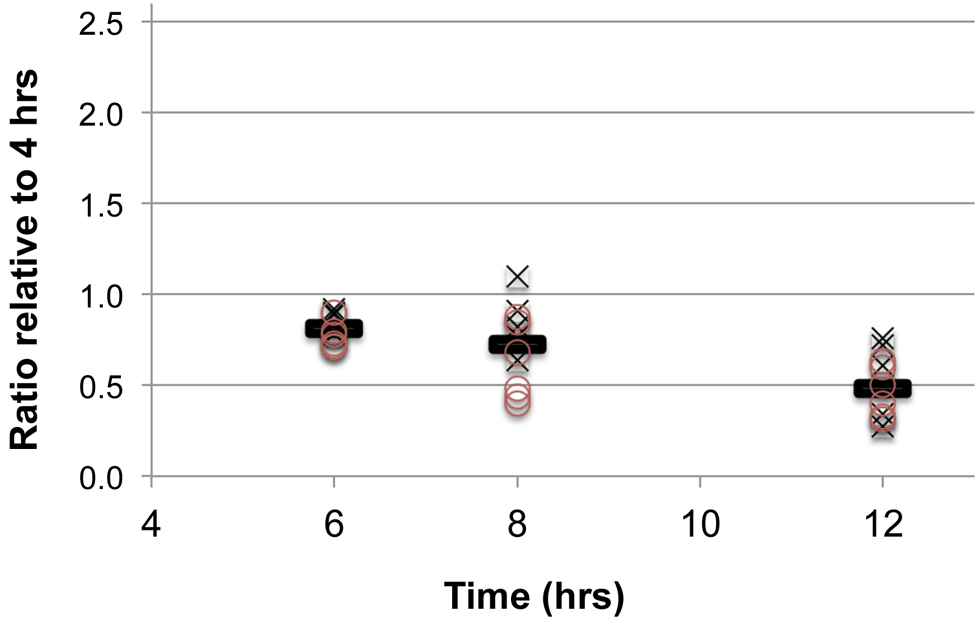

Supplement: Figure S3 — Quantitative evaluation of mesh formation by HUVECs in in vitro angiogenesis assay. Ratios of intact mesh numbers after 6, 8, and 12 hours against the 4-hour values were calculated for each well. Crosses represent values with EBM2 (n = 5) and red circles with EGM2 (n = 6) medium. The horizontal bars represent the means of all measurements at each time point. (TIF) [file pone.0046822.s003.tif]

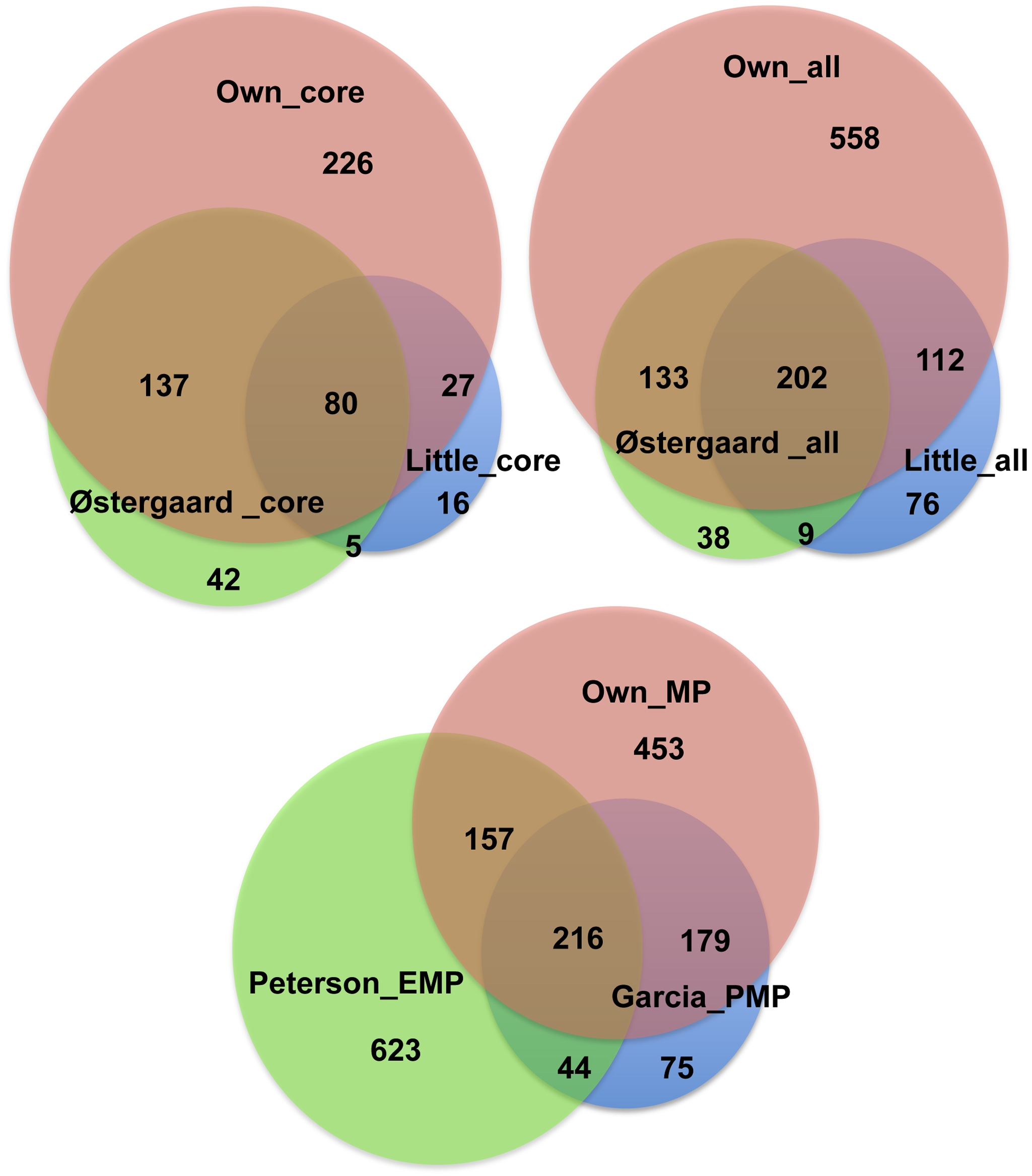

Supplement: Figure S4 — Qualitative assessment of MP associated protein identifications with Venn diagrams. Our own core and complete protein sets were compared with the Uniprot_SwissProt protein identifications of several MP proteome studies published. Østergaard and Little analyzed MPs isolated from human plasma. The complete SwissProt protein sets from endothelial derived MPs (EMP) and activated platelet derived MPs (PMP) from Peterson and Garcia, respectively, were also included in this comparison. Results demonstrate clearly that our MP associated proteins corresponded well with the ones described from other MP and PMP studies, but were less congruent with endothelial cell derived MPs. (TIF) [file pone.0046822.s004.tif]

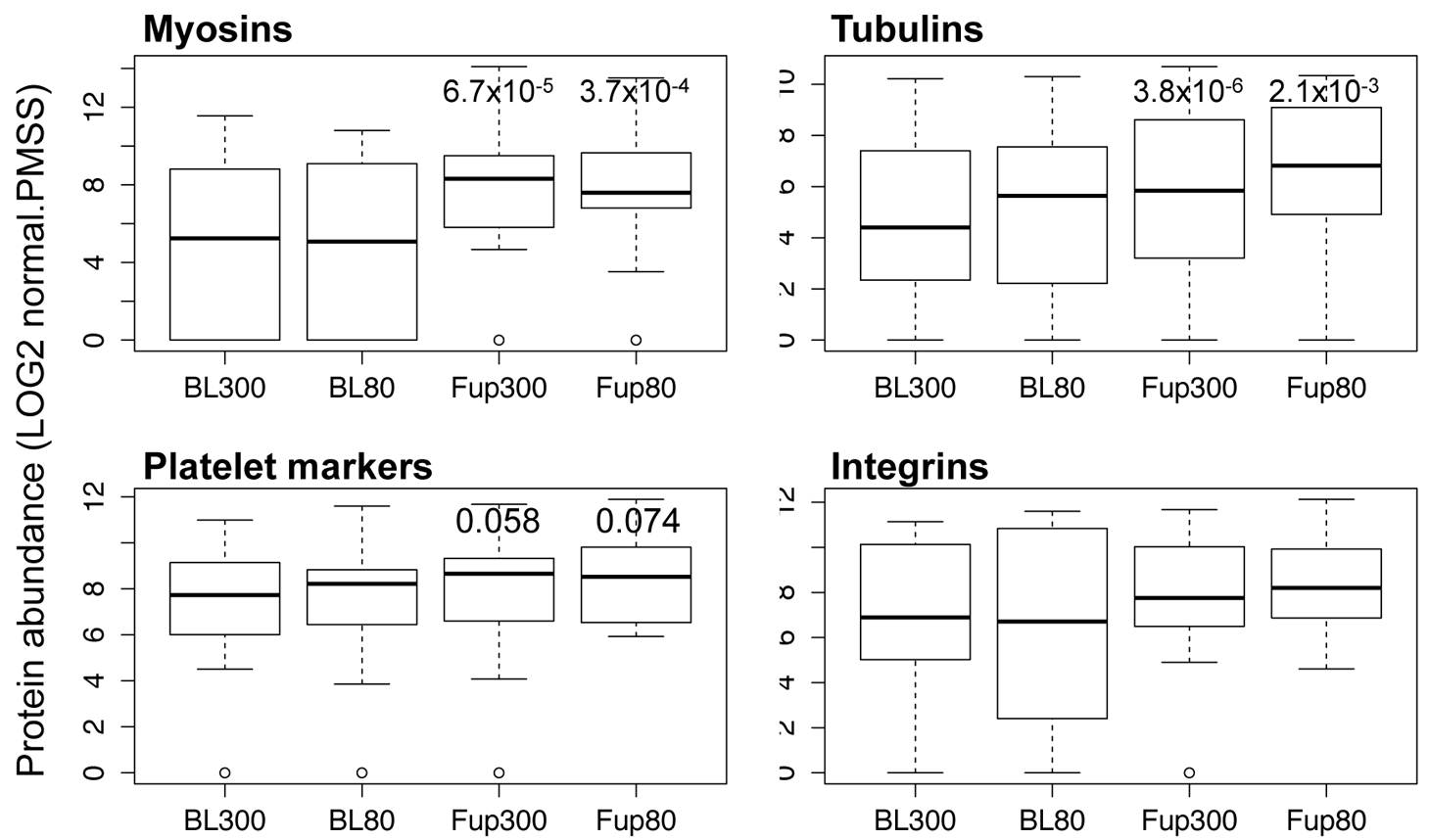

Supplement: Figure S5 — Label-free quantification of protein classes. Boxplot representations of normalized protein abundance values (LOG2 values) of structural cell proteins (top panels) and cell surface proteins (bottom panels). The group of myosin proteins encompasses MYL6, MYH9, MYH10, MYH13, MYH14, MYL9, and ML12A that were identified in 12, 12, 6, 7, 10, 9, and 12 out of the 12 analyzed MP samples, respectively. Tubulins were TBA1A, TBA1B, TBA4A, TBA4B, TBB1, TBB4A, TBB4B, TBB5, TBB6, and TBB8, identified in at least eight MP samples. Platelet markers CD36, CXCL7, ITA2B, PECA1, PLF4 were identified in at least 12 MP samples, except LYAM3 with only seven. Integrins were ITA2, ITA2B, ITA6, ITB1, ITB3, and identified in at least 8 MP samples. When one of the proteins was not identified, its value was set at zero for statistical analysis of P values. P values are given above each box if the likelihood for a statistical difference between BL and Fup was close or better to 95%. (TIF) [file pone.0046822.s005.tif]
