# Supplementary material for: Cause or Effect of Arteriogenesis: Compositional Alterations of Microparticles from CAD Patients Undergoing External Counterpulsation Therapy
Source: PLoS One. 2012 Oct 8;7(10):e46822. doi: 10.1371/journal.pone.0046822 (PMC3466210; doi:10.1371/journal.pone.0046822)
Supplement: Table S1 — List of all identified proteins. (PDF) [file pone.0046822.s006.pdf]

Table S1: List of all identified proteins

					median normalized, LOG2 PMSS values												Confirmed by				
AC	*ID*	*#unique pep*	*cnts PMSS > 40*	*cnts Pat*	*BL300_Pat10*	*BL300_Pat11*	*BL300_Pat5*	*BL80_Pat14*	*BL80_Pat16*	*BL80_Pat7*	*Fup300_Pat10*	*Fup300_Pat11*	*Fup300_Pat5*	*Fup80_Pat14*	*Fup80_Pat16*	*Fup80_Pat7*	*Little_MP*	*Østergaard_MP*	*Garcia_PMP*	*Peterson_EMP*	*# confirmations*
P59190	RAB15_HUMAN	1	9	12	6.621	6.067	5.365	7.302	5.075	5.966	5.676	6.844	4.983	6.063	5.644	6.919					0
Q29960	1C16_HUMAN	2	9	12	4.777	6.246	5.140	7.323	3.798	5.631	6.959	6.406	6.332	3.742	6.690	6.040					0
P01743	HV102_HUMAN	2	12	12	8.241	7.946	6.974	7.715	7.154	6.851	7.035	7.783	6.027	7.034	7.162	5.559					0
P35609	ACTN2_HUMAN	3	5	12	4.451	6.631	3.496	6.764	5.710	2.441	2.770	6.601	2.989	6.042	3.806	2.503			yes		1
P01766	HV305_HUMAN	3	12	12	8.049	8.855	7.991	7.831	7.388	7.548	7.332	7.966	8.210	6.969	7.790	6.343		yes			1
Q15286	RAB35_HUMAN	4	5	12	5.889	4.529	3.901	5.926	3.110	5.076	4.275	5.870	3.804	4.392	2.978	5.773					0
P04222	1C03_HUMAN	4	6	12	3.878	5.614	3.228	5.513	5.975	5.075	4.821	6.604	4.001	4.767	6.475	4.713					0
P02724	GLPA_HUMAN	4	12	12	7.979	7.865	8.063	8.659	6.821	5.299	5.828	6.790	6.720	6.579	6.965	7.143					0
P29972	AQP1_HUMAN	5	9	12	6.258	6.536	6.929	6.162	4.046	6.158	4.185	5.750	5.991	4.441	6.032	7.625					0
P01593	KV101_HUMAN	5	12	12	8.169	7.220	8.540	8.127	7.439	8.915	8.203	7.561	7.145	7.327	6.490	7.513					0
P06309	KV205_HUMAN	5	9	12	7.032	4.290	6.803	6.006	6.722	6.502	6.638	5.194	6.236	6.236	3.260	5.547		yes			1
P01764	HV303_HUMAN	5	12	12	8.002	7.981	8.628	7.781	8.084	7.380	7.647	7.916	8.461	8.200	7.163	6.652	yes				1
Q9NRW1	RAB6B_HUMAN	6	10	12	5.556	6.339	4.246	6.697	5.718	6.952	6.584	7.188	6.727	6.781	5.147	8.328	yes		yes		2
P02775	CXCL7_HUMAN	6	12	12	8.819	9.141	8.381	9.259	8.436	8.675	10.287	9.294	8.632	9.419	9.076	9.809	yes	yes	yes		3
P80748	LV302_HUMAN	6	8	12	7.216	6.439	7.512	6.486	6.044	7.542	5.215	7.171	6.729	4.875	4.720	5.053					0
P68371	TBB2C_HUMAN	7	9	12	5.018	3.467	4.132	7.464	6.576	4.110	5.832	6.463	5.855	6.903	7.165	5.243	yes	yes		yes	3
Q9Y277	VDAC3_HUMAN	7	10	12	5.321	7.167	6.074	5.112	5.513	5.049	6.634	7.411	5.815	4.993	6.751	6.925	yes	yes	yes	yes	4
P04208	LV106_HUMAN	7	9	12	6.814	5.997	7.370	5.775	5.296	7.697	6.639	5.721	6.822	4.759	5.735	6.136					0
P04207	KV308_HUMAN	7	11	12	7.338	5.750	7.275	6.438	6.367	7.608	5.847	6.941	6.382	6.682	5.674	5.850					0
P18428	LBP_HUMAN	8	11	12	5.863	6.701	6.208	5.466	6.780	5.859	7.413	7.573	6.881	6.506	4.586	6.597	yes	yes			2
P51148	RAB5C_HUMAN	8	7	12	5.337	5.538	6.803	5.385	5.640	6.315	5.190	6.653	4.774	5.214	5.323	6.618	yes	yes	yes	yes	4
P05141	ADT2_HUMAN	8	10	12	6.220	6.746	5.610	5.228	5.170	6.498	7.054	7.063	5.487	5.831	6.598	7.106	yes	yes		yes	3
P06310	KV206_HUMAN	8	12	12	7.595	5.850	8.126	6.892	6.056	7.533	7.240	6.486	7.328	5.993	6.188	6.508	yes	yes			2
Q7Z794	K2C1B_HUMAN	9	12	12	7.859	7.234	7.663	8.067	9.330	8.466	6.161	7.263	9.780	9.909	9.088	8.184					0
P04792	HSPB1_HUMAN	9	8	12	5.747	5.384	4.347	7.268	6.023	6.576	5.693	7.405	5.363	6.360	4.899	5.280	yes	yes	yes	yes	4
P61981	1433G_HUMAN	9	11	12	5.741	6.376	6.008	6.042	6.301	6.367	5.982	7.357	5.747	5.064	6.062	6.244	yes	yes	yes	yes	4
P61225	RAP2B_HUMAN	9	10	12	6.483	6.591	7.111	6.509	4.757	5.386	5.857	6.783	5.883	5.850	4.843	7.966		yes	yes	yes	3
P01591	IGJ_HUMAN	9	10	12	7.867	6.646	7.780	4.204	6.960	6.500	6.343	5.039	6.759	5.754	7.068	6.024	yes	yes			2
P01602	KV110_HUMAN	9	11	12	7.933	6.364	7.818	7.632	7.039	8.739	8.366	7.868	7.144	6.912	5.774	7.294	yes	yes			2
P61006	RAB8A_HUMAN	10	8	12	2.195	5.463	5.414	6.037	5.550	5.229	5.787	6.859	4.338	5.907	4.970	6.837		yes	yes		2
P09211	GSTP1_HUMAN	10	9	12	7.163	7.568	6.904	7.092	5.413	7.322	4.940	8.103	5.625	7.634	4.916	7.323	yes	yes	yes	yes	4
O15145	ARPC3_HUMAN	10	10	12	6.397	7.359	5.749	7.346	5.248	6.077	6.981	8.086	6.144	6.669	5.507	7.428	yes	yes		yes	3
P04179	SODM_HUMAN	10	10	12	3.933	7.470	6.415	6.767	5.718	6.645	8.033	7.732	6.409	6.740	5.260	8.556	yes	yes	yes		3
P00915	CAH1_HUMAN	10	9	12	7.507	7.381	7.910	6.194	5.092	7.069	6.182	6.513	5.393	4.301	6.144	6.825	yes		yes		2
P06313	KV403_HUMAN	10	12	12	8.752	7.286	9.452	8.449	8.236	8.995	8.192	8.107	8.979	8.554	7.328	7.790					0
P27348	1433T_HUMAN	11	7	12	4.354	6.170	5.300	6.268	5.877	4.919	3.966	7.399	5.237	5.570	4.799	5.269	yes	yes	yes	yes	4
P23284	PPIB_HUMAN	11	10	12	6.314	7.528	6.785	7.292	5.837	6.463	6.400	8.013	4.449	6.294	4.489	6.745	yes	yes	yes	yes	4
P14770	GPIX_HUMAN	11	12	12	5.878	7.447	6.527	8.073	6.193	6.643	7.966	7.620	6.595	7.653	7.786	8.056	yes	yes	yes		3
P13224	GP1BB_HUMAN	11	12	12	6.962	6.654	6.638	7.332	6.226	7.422	6.357	7.557	6.154	7.891	6.772	8.044	yes	yes	yes		3
P60660	MYL6_HUMAN	11	12	12	9.018	8.816	8.852	9.151	6.341	9.097	9.911	9.698	8.744	8.594	9.651	10.050	yes		yes	yes	3
O00161	SNP23_HUMAN	12	9	12	5.198	5.735	3.845	6.467	6.007	5.343	6.768	7.453	5.014	5.542	5.188	7.050		yes	yes	yes	3
P00387	NB5R3_HUMAN	12	12	12	6.034	7.448	5.774	6.665	6.034	6.433	6.841	8.045	6.226	5.466	7.035	7.754	yes	yes	yes	yes	4
Q15084	PDIA6_HUMAN	12	9	12	4.313	6.503	3.677	7.366	6.142	4.059	6.479	7.379	5.419	6.586	6.840	6.917	yes	yes	yes	yes	4
P31146	COR1A_HUMAN	12	6	12	4.612	6.293	3.884	6.786	5.633	4.078	5.211	7.265	4.487	6.204	6.738	4.434	yes	yes	yes		3
P61026	RAB10_HUMAN	12	12	12	6.733	7.015	6.387	7.424	6.160	7.358	7.639	8.048	6.540	6.947	6.091	8.569	yes	yes	yes	yes	4
Q15907	RB11B_HUMAN	12	12	12	5.512	7.507	6.492	7.133	6.827	7.538	7.043	8.298	6.715	6.738	6.366	7.608	yes		yes		2
P11166	GTR1_HUMAN	12	11	12	6.966	8.782	7.368	8.407	6.964	4.137	5.823	7.587	7.327	6.591	8.953	7.774	yes	yes		yes	3
P05090	APOD_HUMAN	12	12	12	8.972	6.524	9.473	8.023	7.465	8.924	7.517	7.796	7.821	6.563	6.414	7.348	yes				1
O00299	CLIC1_HUMAN	13	12	12	7.432	7.805	7.429	7.293	6.450	7.866	6.618	8.422	6.799	6.244	6.375	7.001	yes	yes	yes	yes	4
P16671	CD36_HUMAN	13	11	12	6.987	8.539	6.887	8.512	7.480	7.791	4.082	8.678	6.024	7.464	6.011	6.043	yes	yes	yes		3
P07195	LDHB_HUMAN	13	11	12	4.754	7.986	4.873	7.082	6.835	6.849	6.184	8.282	6.450	6.144	6.939	6.085	yes	yes	yes	yes	4
P36955	PEDF_HUMAN	13	11	12	7.151	5.171	6.520	6.872	5.563	7.237	6.857	6.656	5.869	5.627	6.228	6.477					0
P52907	CAZA1_HUMAN	13	11	12	6.341	7.263	5.786	5.960	5.046	6.745	6.757	7.163	5.971	6.058	6.236	7.295	yes	yes	yes	yes	4
Q9H0U4	RAB1B_HUMAN	13	11	12	7.820	7.235	8.011	7.747	6.744	7.368	8.506	8.023	7.654	7.391	5.787	9.289		yes		yes	2
P07737	PROF1_HUMAN	13	12	12	7.890	10.414	8.070	9.834	7.672	9.046	9.331	10.327	8.254	9.046	8.563	8.936	yes	yes	yes	yes	4
P02743	SAMP_HUMAN	13	11	12	8.438	6.695	8.144	7.006	6.879	9.026	7.582	7.086	6.537	7.386	4.928	6.362		yes			1
P02763	A1AG1_HUMAN	13	12	12	8.459	7.130	8.247	8.550	7.941	9.209	8.699	7.670	8.789	7.636	7.953	7.116	yes	yes			2
P04433	KV309_HUMAN	13	11	12	8.320	6.562	7.974	7.010	6.880	7.492	7.920	7.129	7.225	7.807	5.800	6.702	yes	yes			2

P07203	GPX1_HUMAN	14	7	12	5.635	6.081	4.154	6.265	3.832	6.535	6.174	7.500	5.065	3.667	4.917	7.596			yes	yes	2
P10316	1A69_HUMAN	14	12	12	6.032	6.578	4.692	6.906	7.686	6.000	5.489	6.575	5.975	5.886	6.541	5.606		yes			1
P07359	GP1BA_HUMAN	14	11	12	7.120	7.572	6.475	5.761	5.390	7.136	5.626	7.506	6.573	6.438	7.341	5.615	yes		yes		2
P01019	ANGT_HUMAN	14	10	12	7.623	6.245	6.893	5.692	5.375	7.129	6.520	5.959	7.240	5.288	6.762	5.372		yes			1
P48059	LIMS1_HUMAN	14	12	12	4.927	8.190	5.034	7.399	7.318	5.820	7.094	8.456	6.463	7.150	7.504	6.354	yes		yes		2
P01861	IGHG4_HUMAN	14	12	12	8.711	7.785	8.188	8.860	8.201	8.035	9.312	8.878	8.498	7.812	7.044	7.384		yes			1
Q9HBI1	PARVB_HUMAN	14	12	12	6.946	8.486	6.798	7.331	7.219	7.101	6.376	8.658	7.285	5.664	7.529	6.920	yes	yes	yes		3
P02760	AMBP_HUMAN	14	11	12	8.204	7.507	8.408	7.051	5.298	9.199	8.385	7.837	8.309	6.435	6.328	7.576		yes			1
P02753	RET4_HUMAN	14	10	12	8.072	6.416	8.603	6.828	5.246	9.526	8.515	6.896	7.744	6.637	4.683	8.374					0
P00491	PNPH_HUMAN	15	10	12	5.964	7.572	5.835	7.482	5.104	6.062	6.211	8.616	5.359	5.672	6.039	5.513	yes	yes	yes	yes	4
P08567	PLEK_HUMAN	15	12	12	6.612	9.292	6.940	8.981	8.632	7.624	7.811	9.550	8.210	6.098	8.164	7.942	yes	yes	yes		3
P40926	MDHM_HUMAN	15	11	12	6.119	7.667	5.552	5.319	5.667	7.061	7.366	7.608	6.014	4.253	7.191	7.824	yes	yes	yes	yes	4
P51149	RAB7A_HUMAN	15	12	12	6.766	7.572	6.019	6.849	6.065	7.567	6.820	8.282	6.116	7.212	5.995	8.148	yes		yes	yes	3
P02776	PLF4_HUMAN	15	12	12	8.168	9.152	7.289	8.827	8.718	8.008	10.193	9.092	9.217	9.355	8.999	10.390	yes		yes		2
P37802	TAGL2_HUMAN	15	12	12	9.874	9.302	9.252	9.980	7.734	9.498	9.654	9.679	9.098	9.090	9.203	9.915	yes	yes	yes	yes	4
P27169	PON1_HUMAN	15	12	12	8.447	7.715	8.182	8.004	8.648	9.176	7.137	7.919	8.888	7.140	7.780	8.544		yes			1
P61158	ARP3_HUMAN	16	12	12	4.932	7.352	5.411	7.689	6.815	5.749	6.348	7.984	5.971	6.764	7.123	6.700	yes	yes	yes	yes	4
P07237	PDIA1_HUMAN	16	9	12	4.856	6.804	4.799	4.978	5.577	4.412	5.910	7.302	4.559	5.588	7.312	6.064	yes	yes	yes	yes	4
P30041	PRDX6_HUMAN	16	11	12	6.248	6.752	6.735	7.282	6.291	6.282	5.612	8.081	5.659	7.105	5.759	6.693	yes	yes	yes	yes	4
Q15404	RSU1_HUMAN	16	12	12	7.111	8.407	7.237	7.946	6.791	7.578	7.500	8.370	7.339	7.215	7.030	7.493	yes		yes		2
O00194	RB27B_HUMAN	16	12	12	7.998	7.733	7.656	8.498	7.559	8.126	8.287	8.929	7.460	8.206	7.257	8.840	yes	yes	yes		3
P04899	GNAI2_HUMAN	16	11	12	6.061	8.066	6.490	5.483	7.098	6.847	6.963	7.716	7.096	5.293	7.664	7.707	yes		yes	yes	3
P60174	TPIS_HUMAN	17	10	12	6.931	7.184	6.963	7.232	6.599	6.958	5.107	8.513	5.280	7.025	6.038	6.363	yes		yes	yes	3
O95810	SDPR_HUMAN	17	12	12	6.198	9.146	6.831	8.982	8.495	6.867	5.466	9.290	7.482	8.252	7.590	7.455	yes	yes	yes		3
P23528	COF1_HUMAN	17	12	12	8.286	8.978	8.180	9.065	6.133	7.578	8.481	9.218	8.033	8.588	7.700	8.632	yes	yes	yes	yes	4
O14791	APOL1_HUMAN	17	11	12	8.410	7.767	7.281	6.360	6.454	7.981	7.212	7.607	7.479	4.551	6.872	7.857	yes	yes			2
P27797	CALR_HUMAN	18	10	12	5.277	7.057	5.483	5.001	6.800	5.521	5.951	7.580	5.162	7.588	7.273	6.109	yes	yes		yes	3
P05556	ITB1_HUMAN	18	12	12	6.696	8.505	5.318	7.177	6.591	6.426	7.758	9.055	6.428	7.171	8.268	8.199	yes	yes	yes	yes	4
P19105	ML12A_HUMAN	18	12	12	8.665	8.663	8.738	9.517	7.148	8.476	9.504	9.211	8.814	9.083	9.141	10.032		yes		yes	2
P18135	KV312_HUMAN	18	12	12	10.597	7.802	9.026	9.009	8.214	9.434	8.460	8.740	8.351	8.851	7.335	7.378	yes				1
P04004	VTNC_HUMAN	18	11	12	8.850	9.265	8.807	9.168	9.739	9.197	8.378	9.138	8.317	8.909	5.839	8.018	yes	yes	yes	yes	4
Q14254	FLOT2_HUMAN	19	8	12	6.568	6.725	7.825	6.791	5.630	3.706	4.439	5.914	5.296	5.110	8.519	6.624			yes	yes	2
P30101	PDIA3_HUMAN	19	12	12	5.983	7.866	5.339	6.678	7.091	5.074	6.941	8.226	5.425	7.163	8.112	7.366	yes	yes	yes	yes	4
P50552	VASP_HUMAN	19	11	12	5.241	7.086	5.104	6.741	5.745	6.234	5.241	7.297	5.922	6.940	7.913	5.649	yes	yes	yes		3
P06753	TPM3_HUMAN	19	11	12	4.465	8.787	7.900	8.704	7.928	6.939	7.793	8.963	6.748	8.245	8.822	7.272	yes	yes	yes	yes	4
P06733	ENOA_HUMAN	20	12	12	6.466	8.599	6.753	8.533	7.089	6.857	6.677	8.588	6.949	6.056	7.541	6.898	yes	yes		yes	3
P02748	CO9_HUMAN	20	10	12	5.861	7.367	7.066	6.373	7.364	6.690	6.003	7.346	7.232	7.662	4.192	4.689	yes	yes			2
P02652	APOA2_HUMAN	20	12	12	9.168	7.181	8.613	7.427	7.749	9.362	8.576	7.684	9.581	8.333	7.721	8.285		yes			1
P01042	KNG1_HUMAN	20	12	12	8.230	7.987	8.075	7.506	6.436	8.759	7.094	7.980	7.402	6.849	7.125	5.581	yes	yes			2
Q01518	CAP1_HUMAN	21	12	12	5.607	9.228	5.332	7.870	7.347	5.610	5.979	9.467	6.744	7.657	8.158	6.967	yes	yes	yes	yes	4
P06576	ATPB_HUMAN	21	10	12	5.333	7.390	4.262	7.259	7.219	4.456	7.191	7.894	7.272	6.747	8.595	7.271	yes	yes		yes	3
P62258	1433E_HUMAN	21	12	12	6.974	8.107	7.062	7.300	7.294	7.057	7.402	8.431	6.975	6.376	7.312	7.524	yes	yes	yes	yes	4
P01859	IGHG2_HUMAN	21	12	12	8.754	7.430	8.645	9.639	8.236	8.844	9.361	8.952	8.919	8.146	7.450	8.301	yes				1
P0CG05	LAC2_HUMAN	21	12	12	11.291	9.097	11.349	10.503	10.056	11.655	10.862	8.937	10.252	9.621	8.507	9.960	yes	yes			2
P04196	HRG_HUMAN	21	12	12	8.251	8.938	8.015	8.258	7.897	9.389	5.698	8.012	7.661	7.630	6.561	6.520	yes	yes			2
P04217	A1BG_HUMAN	21	12	12	8.218	8.962	8.689	8.525	8.151	8.319	7.355	8.045	7.853	7.946	5.858	6.632		yes			1
P09493	TPM1_HUMAN	22	12	12	5.809	8.175	7.599	8.304	7.785	6.369	6.986	8.784	5.519	7.285	8.445	7.255	yes		yes	yes	3
P04406	G3P_HUMAN	22	12	12	8.553	9.529	8.439	8.270	8.522	8.328	7.808	9.275	8.614	7.675	8.313	8.725	yes	yes	yes	yes	4
P25311	ZA2G_HUMAN	22	11	12	7.845	7.530	7.262	7.589	6.380	8.303	7.621	7.214	7.639	4.689	6.705	6.775		yes			1
P02656	APOC3_HUMAN	22	12	12	10.243	8.684	9.546	8.687	8.299	10.159	10.316	8.674	10.396	8.346	8.653	10.213	yes				1
P02765	FETUA_HUMAN	23	12	12	8.647	8.367	7.035	8.333	7.300	7.875	7.021	8.508	7.925	8.250	7.768	7.540					0
Q13418	ILK_HUMAN	24	12	12	5.247	8.149	5.269	8.082	7.163	5.545	6.690	8.696	6.902	7.500	8.078	7.066	yes	yes	yes		3
P14618	KPYM_HUMAN	25	12	12	6.237	8.990	6.087	7.510	7.740	6.094	6.027	9.357	6.250	7.646	8.070	6.545	yes	yes	yes	yes	4
P68363	TBA1B_HUMAN	25	12	12	7.955	9.823	7.402	9.578	9.263	7.177	9.661	10.199	8.899	10.045	9.967	9.345		yes			1
P04075	ALDOA_HUMAN	25	12	12	6.913	9.161	6.824	8.046	8.011	6.854	6.749	8.972	7.312	5.492	7.763	7.320	yes	yes	yes	yes	4
O00151	PDLI1_HUMAN	25	11	12	7.166	8.688	7.112	7.261	7.705	7.330	7.742	8.789	7.359	5.278	7.774	8.001	yes	yes	yes		3
P01860	IGHG3_HUMAN	25	12	12	10.689	9.711	8.946	10.254	8.694	9.758	10.891	9.942	9.614	9.118	8.794	9.598		yes			1
P02746	C1QB_HUMAN	25	12	12	10.096	8.949	7.485	8.640	9.170	8.455	11.444	8.382	11.443	7.790	10.767	11.680	yes	yes			2
P01876	IGHA1_HUMAN	25	12	12	10.033	9.300	10.434	10.195	10.463	10.064	9.825	10.397	10.281	10.438	8.093	8.102					0
P11021	GRP78_HUMAN	26	12	12	6.128	9.017	6.824	8.226	7.684	5.841	6.260	8.737	5.500	7.777	7.016	6.383	yes	yes	yes	yes	4
P48735	IDHP_HUMAN	26	12	12	5.372	7.858	4.884	8.000	7.430	5.975	7.234	8.071	6.870	5.888	8.273	7.527	yes	yes	yes	yes	4
P07437	TBB5_HUMAN	26	12	12	7.090	9.266	6.453	9.467	8.310	6.257	8.614	9.956	8.118	9.098	9.651	8.666			yes	yes	2

Q15942	ZYX_HUMAN	26	12	12	6.571	9.293	7.321	9.367	8.171	7.850	5.874	9.558	6.620	8.364	7.019	6.423			yes		1
P63104	1433Z_HUMAN	26	12	12	8.343	9.730	8.716	9.504	9.451	8.608	8.934	10.024	8.888	9.408	9.358	9.210	yes	yes	yes	yes	4
P27105	STOM_HUMAN	26	12	12	9.263	9.987	9.953	9.594	9.311	8.806	9.222	10.019	9.361	9.405	9.808	10.430	yes	yes	yes	yes	4
P04003	C4BPA_HUMAN	26	12	12	7.032	8.671	7.530	8.844	8.776	7.869	7.684	8.204	7.890	8.451	6.668	6.216	yes				1
Q93084	AT2A3_HUMAN	27	12	12	6.380	8.645	5.407	8.419	8.005	7.086	6.449	8.898	5.807	8.045	7.532	7.864	yes				1
P61224	RAP1B_HUMAN	28	12	12	8.816	9.192	9.030	9.416	8.169	9.028	9.388	9.524	8.755	9.060	7.571	10.176	yes	yes	yes		3
P16284	PECA1_HUMAN	29	11	12	6.006	6.750	5.284	5.854	7.376	6.446	7.378	8.158	5.238	7.526	7.853	8.040	yes	yes	yes	yes	4
O43707	ACTN4_HUMAN	29	9	12	5.694	9.033	5.133	7.779	6.257	5.729	5.104	9.362	5.175	7.635	5.039	5.195	yes	yes	yes	yes	4
P14625	ENPL_HUMAN	29	10	12	6.544	8.649	5.520	8.675	6.945	6.477	5.825	9.414	4.896	8.261	5.864	5.127	yes	yes	yes	yes	4
P11142	HSP7C_HUMAN	29	12	12	6.028	9.831	5.641	8.979	8.601	5.930	6.154	9.706	6.334	8.823	7.080	6.367	yes	yes	yes	yes	4
P10909	CLUS_HUMAN	29	12	12	10.461	9.875	10.107	8.837	10.181	9.759	9.069	9.489	9.647	8.884	9.262	8.950	yes	yes	yes		3
P01011	AACT_HUMAN	29	12	12	8.568	8.947	9.217	8.101	8.459	9.271	9.612	8.582	9.800	8.605	7.542	8.280		yes			1
P05155	IC1_HUMAN	31	11	12	9.412	8.608	9.421	9.543	9.056	9.923	6.974	8.874	7.706	9.967	4.922	6.663	yes	yes			2
P02649	APOE_HUMAN	33	12	12	11.087	10.096	10.316	9.296	10.603	9.998	9.716	9.599	9.658	10.443	9.508	9.563	yes	yes	yes		3
P02747	C1QC_HUMAN	33	12	12	10.902	8.487	8.964	7.713	7.730	9.122	10.546	7.871	11.166	7.244	10.699	11.406	yes	yes			2
P67936	TPM4_HUMAN	34	12	12	7.688	9.861	8.183	9.970	9.445	8.429	8.896	10.002	7.648	9.626	9.880	8.884	yes	yes			2
P69905	HBA_HUMAN	34	12	12	11.095	11.612	11.374	10.626	9.790	10.612	11.598	10.967	10.419	10.822	10.683	11.316	yes	yes		yes	3
Q9H4B7	TBB1_HUMAN	36	12	12	7.812	10.222	7.477	10.300	9.562	7.460	9.690	10.690	9.332	9.681	10.341	9.432	yes	yes		yes	3
Q13201	MMRN1_HUMAN	36	12	12	9.119	8.576	8.530	5.388	6.793	8.053	10.341	9.162	8.699	8.577	9.366	10.547	yes	yes	yes	yes	4
P00734	THRB_HUMAN	38	11	12	9.498	8.710	9.691	8.597	8.501	10.246	6.494	7.967	7.213	8.306	5.545	6.347		yes			1
P01008	ANT3_HUMAN	39	12	12	9.100	8.991	9.082	8.121	9.707	9.382	7.909	8.239	9.050	8.384	7.210	7.888		yes			1
P19823	ITIH2_HUMAN	39	12	12	9.652	8.071	8.684	6.079	7.127	10.269	10.137	7.503	9.952	8.485	7.767	9.489		yes			1
P02790	HEMO_HUMAN	39	12	12	8.622	9.486	8.767	9.678	8.965	9.085	8.086	9.102	8.981	9.211	7.084	7.158	yes	yes			2
P00488	F13A_HUMAN	40	12	12	8.107	10.011	8.624	10.132	9.598	8.562	5.536	9.962	6.223	9.556	6.050	5.614	yes	yes	yes		3
P02774	VTDB_HUMAN	41	12	12	9.021	9.201	8.751	9.267	8.567	9.328	9.569	8.853	9.839	9.122	9.103	9.134		yes			1
P02766	TTHY_HUMAN	42	12	12	10.915	9.077	10.344	9.624	7.186	10.794	10.796	9.182	10.458	9.030	8.408	9.882	yes				1
P68871	HBB_HUMAN	42	12	12	11.417	11.970	11.777	11.642	10.153	11.134	11.761	11.341	10.827	11.668	11.290	12.071	yes	yes		yes	3
P00747	PLMN_HUMAN	42	11	12	9.546	9.411	9.319	9.604	9.364	10.089	6.349	9.877	7.879	9.359	4.169	6.360		yes	yes		2
P35527	K1C9_HUMAN	43	12	12	11.097	9.613	10.227	10.053	11.080	11.477	9.640	9.262	12.065	11.655	10.523	11.499	yes	yes		yes	3
P01834	IGKC_HUMAN	43	12	12	13.219	10.793	12.757	11.873	11.469	12.933	12.594	11.582	11.968	11.422	9.997	11.622		yes	yes		2
Q86UX7	URP2_HUMAN	44	12	12	7.419	10.708	8.003	9.676	9.387	8.272	5.982	10.742	7.800	9.588	8.261	8.107	yes	yes	yes	yes	4
P00751	CFAB_HUMAN	47	11	12	9.401	9.066	8.905	9.151	9.214	9.944	7.737	9.139	7.575	9.573	5.790	6.127					0
P02749	APOH_HUMAN	51	12	12	9.006	9.840	9.074	9.744	8.950	8.205	10.688	9.815	10.327	9.652	11.014	10.905	yes				1
P05106	ITB3_HUMAN	52	12	12	9.870	11.137	9.439	11.293	11.505	9.334	8.577	11.586	10.934	12.128	9.536	8.843	yes	yes	yes	yes	4
P01871	IGHM_HUMAN	53	12	12	12.118	10.997	11.098	8.566	10.611	10.312	10.223	8.817	10.569	10.224	9.903	8.770		yes			1
Q14624	ITIH4_HUMAN	55	12	12	10.250	9.144	9.834	8.460	9.851	10.872	8.903	9.814	8.415	9.783	8.741	7.491					0
P06396	GELS_HUMAN	56	12	12	10.621	10.752	9.707	11.074	10.178	10.029	8.396	11.061	9.270	10.635	8.834	9.057	yes	yes	yes		3
P01031	CO5_HUMAN	57	10	12	8.967	7.804	8.390	8.136	7.137	10.150	5.851	8.198	7.012	7.610	5.810	4.116		yes			1
P06727	APOA4_HUMAN	58	12	12	9.375	9.698	9.092	9.888	9.409	10.378	8.689	9.899	10.834	7.100	9.874	8.621	yes	yes			2
P07996	TSP1_HUMAN	69	12	12	8.966	10.433	8.302	7.977	8.322	9.377	12.049	10.429	11.051	10.356	11.260	11.589	yes	yes	yes	yes	4
P18206	VINC_HUMAN	70	12	12	10.167	11.454	10.238	11.008	10.963	10.100	8.700	11.960	9.148	11.349	10.832	9.422	yes	yes	yes	yes	4
P04264	K2C1_HUMAN	70	12	12	12.053	11.255	12.146	11.688	12.688	12.303	10.948	11.107	13.020	13.138	12.646	12.095	yes	yes		yes	3
P08514	ITA2B_HUMAN	70	12	12	10.607	10.989	10.385	11.175	11.598	10.496	10.732	11.674	9.320	11.890	10.602	10.310	yes	yes	yes		3
P08603	CFAH_HUMAN	71	11	12	9.875	7.827	9.292	3.695	6.302	10.447	11.239	8.060	10.449	8.229	8.786	9.476		yes			1
P01009	A1AT_HUMAN	72	12	12	12.126	10.995	12.099	10.268	10.860	11.914	11.952	10.572	12.192	11.565	10.410	10.898	yes	yes			2
P01857	IGHG1_HUMAN	73	12	12	12.417	11.808	12.034	12.364	12.076	12.499	12.950	11.846	12.184	12.243	11.345	11.166					0
P35908	K22E_HUMAN	74	12	12	10.678	10.023	12.700	9.841	11.533	10.902	9.760	9.812	11.570	11.909	11.478	10.830	yes	yes		yes	3
P12259	FA5_HUMAN	78	12	12	9.647	9.616	9.203	8.593	9.277	8.087	10.481	10.045	10.431	10.278	10.741	10.498		yes	yes		2
P13645	K1C10_HUMAN	81	12	12	11.830	10.850	12.436	10.698	12.334	12.064	10.456	10.719	12.987	12.660	12.323	11.823	yes	yes		yes	3
P12814	ACTN1_HUMAN	83	12	12	10.067	11.622	9.625	12.046	12.076	9.969	8.578	12.330	8.585	12.593	9.885	9.277	yes		yes	yes	3
P00450	CERU_HUMAN	88	12	12	10.449	8.950	10.434	6.715	8.038	11.068	10.905	9.400	10.102	9.289	8.228	9.830		yes			1
P02730	B3AT_HUMAN	90	12	12	11.770	12.748	12.445	12.515	11.803	9.484	10.532	11.815	10.241	11.998	12.154	11.890	yes	yes			2
P02675	FIBB_HUMAN	95	12	12	11.631	12.445	11.122	11.921	13.121	11.848	12.203	12.336	12.161	12.148	12.047	11.340	yes	yes	yes		3
P00738	HPT_HUMAN	96	12	12	12.145	10.778	12.510	11.481	9.952	12.758	13.391	10.501	11.992	9.968	10.138	10.395	yes	yes			2
P02679	FIBG_HUMAN	98	12	12	11.132	11.599	10.769	12.019	12.751	11.476	12.270	11.699	11.968	11.628	11.675	11.189	yes		yes		2
P0C0L5	CO4B_HUMAN	102	12	12	11.073	10.965	10.573	10.872	9.816	11.566	10.566	10.811	9.849	9.866	8.791	9.517		yes			1
P02647	APOA1_HUMAN	104	12	12	13.027	10.464	12.802	11.768	11.035	13.159	11.738	11.398	11.930	11.254	10.462	12.487	yes	yes		yes	3
P16157	ANK1_HUMAN	109	12	12	10.287	10.493	10.983	7.537	7.068	5.696	11.325	9.790	11.227	9.940	11.914	11.684	yes	yes			2
P02671	FIBA_HUMAN	110	12	12	12.168	12.672	11.753	12.244	12.997	12.316	12.293	12.448	11.951	12.049	11.758	11.241	yes	yes	yes		3
P60709	ACTB_HUMAN	118	12	12	11.913	14.012	11.637	13.306	12.822	12.293	13.008	14.088	12.672	12.039	13.197	13.315	yes	yes	yes	yes	4
P02751	FINC_HUMAN	153	12	12	9.016	11.949	10.077	6.095	13.309	11.396	11.959	10.067	12.969	10.072	10.730	12.265	yes	yes	yes	yes	4
P02787	TRFE_HUMAN	166	12	12	12.156	12.763	12.607	12.845	12.042	13.456	11.389	12.310	11.830	12.490	9.832	11.102	yes	yes	yes		3

Q9Y490	TLN1_HUMAN	174	12	12	10.949	11.970	11.069	10.610	9.949	11.565	13.690	12.227	13.062	11.762	12.521	12.624	yes	yes	yes	yes	4
P01023	A2MG_HUMAN	174	12	12	13.186	10.814	12.816	10.777	9.785	13.158	13.629	11.574	12.682	11.844	10.768	12.460	yes	yes		yes	3
P11277	SPTB1_HUMAN	197	12	12	11.080	11.536	11.770	7.501	7.441	7.224	12.134	10.642	11.663	11.150	12.882	12.982	yes		yes		2
P01024	CO3_HUMAN	197	12	12	12.811	12.225	12.361	12.641	12.451	13.576	11.450	12.483	11.906	12.761	10.142	10.611	yes	yes			2
P02549	SPTA1_HUMAN	206	12	12	11.283	11.588	12.010	8.066	7.253	7.582	12.435	11.028	11.998	10.904	12.757	12.632	yes	yes			2
P35579	MYH9_HUMAN	213	12	12	10.678	11.554	10.523	9.777	9.646	10.807	14.095	11.709	12.710	11.527	13.212	13.515	yes	yes		yes	3
P21333	FLNA_HUMAN	246	12	12	10.688	12.021	10.502	10.690	9.918	11.152	13.875	12.247	13.097	11.701	12.588	13.697	yes	yes		yes	3
P02768	ALBU_HUMAN	299	12	12	14.451	15.020	14.318	14.586	14.340	14.918	14.882	15.061	15.119	14.574	13.913	14.654		yes	yes	yes	3
P04114	APOB_HUMAN	355	12	12	12.010	9.721	11.406	8.110	7.545	13.073	14.333	10.272	13.328	11.350	11.824	13.409	yes	yes	yes		3
P04350	TBB4_HUMAN	2	0	11	2.343	2.851	0.000	4.221	4.420	2.217	2.579	3.202	2.907	2.467	5.675	3.527		yes	yes	yes	3
P31947	1433S_HUMAN	3	2	11	4.573	5.358	4.738	5.251	5.066	4.167	0.000	5.943	2.631	4.889	5.154	3.356					0
P63261	ACTG_HUMAN	3	11	11	7.851	8.878	7.824	8.511	8.170	7.825	8.546	9.304	0.000	7.179	8.699	8.782					0
O75915	PRAF3_HUMAN	4	8	11	5.659	7.551	5.046	4.532	0.000	5.012	7.397	6.422	6.388	4.653	3.961	7.037	yes	yes		yes	3
P23083	HV103_HUMAN	5	4	11	4.252	5.371	5.391	5.852	3.762	3.484	6.085	6.243	2.871	5.074	4.187	0.000					0
P02042	HBD_HUMAN	5	10	11	8.242	0.000	8.576	7.276	5.793	8.620	8.599	7.450	7.513	5.863	5.519	8.811	yes	yes	yes		3
O75396	SC22B_HUMAN	7	7	11	4.510	5.912	5.861	5.372	4.157	5.088	4.892	7.320	0.000	6.159	5.138	6.692			yes	yes	2
Q8TC12	RDH11_HUMAN	7	6	11	3.473	6.264	0.000	4.676	3.882	5.769	5.883	6.259	4.070	4.329	6.192	6.652		yes	yes	yes	3
P19652	A1AG2_HUMAN	7	9	11	6.515	6.113	6.435	6.124	3.904	7.569	6.775	6.018	7.464	4.889	0.000	5.343	yes	yes			2
P04632	CPNS1_HUMAN	8	8	11	4.514	6.176	5.118	5.542	5.153	4.947	5.777	6.795	5.604	6.173	0.000	6.815	yes		yes		2
P61204	ARF3_HUMAN	8	10	11	7.553	7.635	7.842	7.423	0.000	7.469	6.820	7.863	6.115	5.407	5.799	7.312			yes		1
P30405	PPIF_HUMAN	8	8	11	6.235	5.301	5.946	6.218	0.000	5.894	7.385	6.879	4.389	6.239	4.623	7.391		yes	yes		2
P32119	PRDX2_HUMAN	8	8	11	7.460	6.474	7.608	5.252	5.726	7.034	4.587	6.798	0.000	5.632	3.308	6.945	yes	yes	yes	yes	4
P07951	TPM2_HUMAN	9	8	11	4.858	6.708	1.869	7.441	6.419	4.122	5.646	7.173	0.000	7.015	7.329	5.180	yes	yes	yes	yes	4
P02655	APOC2_HUMAN	9	6	11	8.654	4.306	7.861	5.211	5.049	8.562	7.419	5.568	8.861	0.000	5.022	7.678	yes	yes			2
P01717	LV403_HUMAN	9	10	11	7.390	6.534	7.264	5.956	5.941	7.402	6.864	6.436	6.396	4.624	0.000	5.564		yes			1
P61106	RAB14_HUMAN	10	6	11	5.013	3.939	5.745	4.950	4.347	6.284	3.462	6.734	4.015	6.207	0.000	6.564	yes	yes	yes	yes	4
O75558	STX11_HUMAN	10	10	11	0.000	6.910	3.388	6.011	6.284	5.373	6.330	7.187	5.482	6.178	7.032	5.793		yes	yes		2
P10809	CH60_HUMAN	11	5	11	3.923	6.125	3.212	4.421	4.452	3.541	4.454	6.455	0.000	5.428	6.376	5.503	yes	yes	yes	yes	4
P47755	CAZA2_HUMAN	11	7	11	4.911	6.582	5.456	0.000	2.606	5.526	6.197	6.699	4.534	2.438	5.207	5.822	yes	yes	yes		3
P08697	A2AP_HUMAN	11	7	11	6.254	6.742	6.425	5.365	4.943	6.695	0.000	5.611	5.443	5.590	4.205	3.645		yes			1
P31946	1433B_HUMAN	12	5	11	3.880	2.766	4.013	6.232	3.924	5.489	5.545	6.963	4.649	2.458	0.000	6.440	yes	yes	yes	yes	4
P00918	CAH2_HUMAN	12	7	11	5.683	7.451	5.667	6.016	5.401	5.890	5.701	7.317	0.000	4.200	5.530	4.888	yes	yes	yes		3
P12931	SRC_HUMAN	13	6	11	4.262	6.687	3.455	6.074	5.354	0.000	3.956	7.387	5.183	5.832	6.818	5.308	yes	yes	yes		3
P01594	KV102_HUMAN	14	5	11	6.462	3.015	6.851	4.986	6.021	7.410	6.036	5.354	5.319	5.298	0.000	3.870					0
P61160	ARP2_HUMAN	15	11	11	5.481	7.466	4.784	6.316	6.166	5.522	6.297	8.153	6.419	0.000	6.743	6.572	yes		yes	yes	3
P08779	K1C16_HUMAN	15	7	11	7.560	4.536	6.198	4.932	6.614	7.551	0.000	5.419	7.645	8.492	4.297	6.263	yes	yes		yes	3
P00338	LDHA_HUMAN	15	11	11	6.806	8.065	6.275	6.762	6.706	7.089	7.393	8.277	5.756	0.000	6.619	6.215	yes	yes	yes	yes	4
P05156	CFAI_HUMAN	15	7	11	6.972	4.772	6.864	5.798	0.000	7.700	7.466	4.853	7.054	5.250	4.705	6.048					0
Q04917	1433F_HUMAN	16	11	11	6.284	7.325	6.928	7.243	7.064	7.323	0.000	8.353	6.749	6.583	7.185	6.864	yes	yes	yes		3
Q15485	FCN2_HUMAN	16	10	11	0.000	7.262	7.071	5.444	9.340	6.689	5.620	7.111	9.098	8.208	5.717	7.133	yes				1
Q08495	DEMA_HUMAN	17	10	11	7.329	7.455	7.959	7.664	6.069	0.000	4.289	7.445	5.908	6.691	8.576	6.931					0
P00558	PGK1_HUMAN	17	5	11	5.256	6.784	4.691	7.596	5.789	4.939	3.567	6.879	5.308	0.000	5.326	4.662	yes	yes	yes	yes	4
Q96PD5	PGRP2_HUMAN	17	9	11	6.639	7.524	5.786	5.098	4.592	7.278	0.000	6.763	6.287	6.097	6.101	5.554		yes			1
P25705	ATPA_HUMAN	18	9	11	3.909	6.622	4.137	6.600	6.448	0.000	6.022	7.089	5.856	6.778	7.944	6.954	yes	yes	yes	yes	4
P30740	ILEU_HUMAN	18	6	11	5.311	7.483	4.324	6.998	5.513	4.203	3.784	7.622	4.036	0.000	6.207	3.507	yes	yes	yes		3
P47756	CAPZB_HUMAN	19	10	11	5.050	7.628	0.000	6.082	6.306	6.176	6.934	8.464	5.454	4.471	6.708	6.863	yes		yes	yes	3
P62937	PPIA_HUMAN	19	11	11	7.962	8.005	7.952	8.062	0.000	7.511	9.010	8.638	7.573	7.678	7.450	8.685	yes	yes	yes	yes	4
P00736	C1R_HUMAN	19	9	11	8.037	8.021	7.397	7.916	7.744	8.292	3.836	7.407	6.120	6.296	0.000	4.545	yes	yes			2
P27824	CALX_HUMAN	20	9	11	5.548	7.882	5.426	8.062	7.335	5.398	0.000	8.708	3.647	8.196	6.486	5.106			yes	yes	2
P13647	K2C5_HUMAN	20	10	11	7.458	6.832	8.820	6.593	8.209	7.286	0.000	5.605	9.095	9.272	7.327	7.126	yes	yes		yes	3
P05546	HEP2_HUMAN	20	9	11	6.611	7.424	6.953	7.032	7.400	7.069	3.471	6.892	6.441	7.919	0.000	3.508	yes	yes			2
O75955	FLOT1_HUMAN	21	11	11	7.093	7.675	7.641	8.014	6.155	0.000	5.324	7.569	5.953	6.531	8.720	7.700		yes	yes	yes	3
P09871	C1S_HUMAN	22	7	11	8.237	6.855	7.813	7.066	6.020	8.405	5.047	6.726	4.025	4.586	0.000	4.585	yes	yes			2
P02745	C1QA_HUMAN	25	11	11	9.359	7.306	6.957	7.109	7.025	6.840	9.861	6.747	10.279	0.000	9.370	10.509	yes	yes			2
P07900	HS90A_HUMAN	26	9	11	6.535	8.749	6.228	8.518	7.710	5.400	4.561	9.003	0.000	7.413	5.977	4.427	yes	yes	yes	yes	4
P02533	K1C14_HUMAN	26	10	11	8.446	5.573	7.941	7.122	7.793	8.042	0.000	6.418	8.828	8.743	7.575	7.355	yes	yes		yes	3
P55072	TERA_HUMAN	31	9	11	6.979	8.798	6.585	8.231	6.672	6.188	4.773	9.535	5.187	7.647	5.937	0.000	yes	yes	yes	yes	4
P19827	ITIH1_HUMAN	36	10	11	9.021	8.034	9.043	0.000	5.387	10.060	10.686	6.672	10.488	7.696	7.903	10.212		yes			1
P08519	APOA_HUMAN	41	9	11	7.661	5.588	0.000	4.738	5.135	7.660	10.936	6.884	9.244	6.082	8.538	9.412	yes	yes	yes		3
P16452	EPB42_HUMAN	42	10	11	7.646	10.637	8.660	9.435	8.628	0.000	4.623	9.309	6.373	8.829	8.743	8.374	yes				1
P11171	41_HUMAN	50	10	11	9.543	10.701	10.732	9.845	8.463	0.000	5.174	9.192	7.173	8.849	9.482	8.475	yes	yes			2
P04275	VWF_HUMAN	64	11	11	5.664	8.105	6.357	0.000	8.635	8.073	8.801	8.347	9.002	6.660	9.262	9.965	yes	yes	yes	yes	4

Q9BUF5	TBB6_HUMAN	1	1	10	2.307	2.864	0.000	5.818	4.393	0.000	2.540	2.953	2.860	4.382	4.911	3.517		yes		yes	2
Q9H853	TBA4B_HUMAN	2	4	10	0.000	5.431	0.000	5.457	4.938	2.202	3.808	7.036	3.735	6.810	6.829	3.530					0
Q3ZCM7	TBB8_HUMAN	3	3	10	3.175	5.533	1.884	6.776	0.000	0.000	3.595	6.335	4.339	5.225	5.976	2.478					0
P62820	RAB1A_HUMAN	4	6	10	0.000	4.603	5.121	5.410	4.862	5.518	0.000	5.791	5.006	5.775	3.126	7.057	yes	yes	yes	yes	4
Q7Z406	MYH14_HUMAN	4	6	10	5.249	4.093	2.895	0.000	0.000	5.077	7.713	4.751	6.134	4.330	7.066	7.062		yes		yes	2
P01613	KV121_HUMAN	4	7	10	7.199	4.535	7.288	6.047	4.823	7.868	5.560	6.134	6.080	0.000	0.000	5.162		yes			1
P01781	HV320_HUMAN	5	9	10	5.447	7.369	5.701	7.169	7.098	5.297	0.000	7.264	5.916	7.278	4.414	0.000					0
P01610	KV118_HUMAN	5	4	10	5.753	0.000	6.313	4.819	3.684	6.958	4.944	5.956	5.307	4.143	0.000	3.682	yes	yes			2
P01714	LV301_HUMAN	5	8	10	6.700	6.459	7.395	6.199	5.193	7.463	0.000	7.021	6.557	4.550	5.918	0.000	yes	yes			2
P04439	1A03_HUMAN	6	3	10	0.000	4.358	0.000	5.912	4.954	4.242	2.978	7.514	3.248	2.972	5.098	5.793					0
P49755	TMEDA_HUMAN	6	7	10	5.050	5.135	4.734	5.140	0.000	5.227	6.629	6.233	5.086	5.584	0.000	7.251		yes	yes	yes	3
P63000	RAC1_HUMAN	7	10	10	6.857	6.741	6.048	6.405	0.000	6.927	6.449	7.106	5.953	5.787	0.000	7.370	yes		yes	yes	3
P11169	GTR3_HUMAN	7	3	10	0.000	5.251	0.000	4.844	4.417	4.326	5.205	5.134	5.876	5.267	6.308	5.994	yes	yes	yes		3
P61163	ACTZ_HUMAN	7	2	10	4.665	5.920	0.000	5.166	4.580	3.523	4.726	6.372	4.799	0.000	5.536	3.658	yes		yes	yes	3
Q06830	PRDX1_HUMAN	8	4	10	5.254	4.982	2.945	5.808	0.000	5.729	4.462	6.582	0.000	2.624	3.322	4.994	yes	yes	yes	yes	4
Q99439	CNN2_HUMAN	8	7	10	0.000	7.479	3.704	4.649	5.474	6.393	7.128	7.795	5.896	3.515	6.807	0.000	yes		yes	yes	3
P52566	GDIR2_HUMAN	9	6	10	5.460	6.093	6.098	5.897	4.510	5.800	0.000	6.872	3.840	4.952	0.000	4.221	yes	yes	yes		3
P68366	TBA4A_HUMAN	9	9	10	4.676	7.621	4.882	7.672	7.551	0.000	0.000	8.073	6.630	7.820	7.483	6.838	yes	yes		yes	3
P61586	RHOA_HUMAN	10	9	10	5.700	6.990	5.276	6.028	0.000	6.871	7.025	7.471	5.624	4.136	0.000	7.184			yes		1
Q9NYL9	TMOD3_HUMAN	10	7	10	4.588	6.683	0.000	5.245	4.900	5.001	5.637	6.709	5.617	0.000	6.178	6.670	yes	yes			2
P16615	AT2A2_HUMAN	11	3	10	3.930	5.666	0.000	4.758	3.720	2.088	5.313	5.890	0.000	4.299	4.938	4.182	yes	yes	yes	yes	4
P02654	APOC1_HUMAN	11	7	10	9.284	0.000	8.556	0.000	4.883	9.316	7.538	4.517	9.817	7.532	6.112	3.415	yes	yes			2
P54920	SNAA_HUMAN	12	5	10	3.975	6.589	4.244	5.171	0.000	5.887	6.094	6.877	0.000	3.723	5.492	5.520	yes		yes	yes	3
Q9NQC3	RTN4_HUMAN	12	9	10	0.000	7.281	6.040	6.530	5.481	0.000	6.145	7.618	5.364	6.448	7.060	7.324	yes	yes	yes	yes	4
Q9Y624	JAM1_HUMAN	12	8	10	5.445	6.840	4.231	0.000	5.342	6.011	6.606	6.953	5.794	0.000	5.962	6.839	yes		yes	yes	3
P04259	K2C6B_HUMAN	14	5	10	5.370	2.885	0.000	4.776	5.849	6.794	0.000	3.016	5.877	7.873	5.603	3.876	yes	yes			2
Q9NZN3	EHD3_HUMAN	14	3	10	0.000	6.289	3.294	3.900	5.636	4.247	3.726	7.663	0.000	3.846	5.530	4.194	yes	yes	yes	yes	4
P40197	GPV_HUMAN	15	5	10	3.568	8.100	3.521	6.420	5.885	4.031	0.000	8.001	0.000	6.289	4.382	5.054	yes	yes	yes		3
P50395	GDIB_HUMAN	16	4	10	5.345	6.560	0.000	6.618	3.929	4.458	0.000	7.250	4.764	4.976	5.508	4.562			yes	yes	2
P62873	GBB1_HUMAN	16	9	10	5.477	8.147	5.410	0.000	5.149	7.092	7.544	7.083	6.915	0.000	6.607	7.920	yes	yes	yes	yes	4
O43866	CD5L_HUMAN	17	9	10	8.408	8.429	6.709	0.000	7.564	7.229	8.046	5.562	8.717	0.000	7.988	6.566	yes			yes	2
Q9ULV4	COR1C_HUMAN	18	8	10	0.000	7.705	0.000	7.045	6.069	4.135	6.196	8.139	4.585	6.471	7.603	6.280			yes	yes	2
Q00013	EM55_HUMAN	19	7	10	5.287	6.173	6.505	5.216	4.816	0.000	0.000	6.160	4.729	5.745	7.681	6.061	yes		yes		2
P13671	CO6_HUMAN	22	7	10	7.520	7.003	7.215	6.660	0.000	8.459	3.697	7.342	6.041	4.718	5.298	0.000					0
P35611	ADDA_HUMAN	24	10	10	6.928	8.323	8.041	8.280	5.639	0.000	0.000	8.120	5.822	6.990	7.737	6.012					0
P43652	AFAM_HUMAN	25	9	10	8.205	7.888	8.216	7.058	5.919	8.991	0.000	6.923	5.796	6.302	0.000	5.073					0
Q8WZ42	TITIN_HUMAN	37	9	10	7.130	6.766	5.699	0.000	0.000	7.451	5.849	6.952	4.609	7.001	6.284	5.329		yes			1
Q00610	CLH1_HUMAN	52	10	10	5.573	7.114	6.925	0.000	0.000	6.569	8.937	7.626	5.854	7.408	8.702	8.993	yes	yes	yes	yes	4
P24928	RPB1_HUMAN	67	10	10	6.611	8.583	8.467	9.117	6.345	0.000	10.241	0.000	8.115	6.061	6.561	9.019					0
P0CG04	LAC1_HUMAN	1	7	9	8.433	6.547	7.735	0.000	0.000	3.212	7.512	6.694	7.494	0.000	4.592	6.686					0
P13929	ENOB_HUMAN	1	2	9	3.675	4.815	4.388	5.845	4.416	4.041	0.000	5.689	4.432	0.000	4.882	0.000			yes		1
P17066	HSP76_HUMAN	1	6	9	0.000	7.611	0.000	6.872	6.180	3.627	0.000	7.269	3.161	6.579	6.042	3.625		yes	yes		2
P30464	1B15_HUMAN	2	0	9	2.985	3.834	1.985	2.526	2.791	0.000	2.912	2.779	2.569	0.000	3.222	0.000					0
P01599	KV107_HUMAN	3	2	9	5.258	0.000	5.658	4.237	4.547	4.783	4.156	4.611	4.532	4.288	0.000	0.000					0
P01621	KV303_HUMAN	3	0	9	0.000	2.981	1.847	3.474	5.009	2.191	4.506	4.425	0.000	4.279	0.000	2.287		yes			1
P35542	SAA4_HUMAN	3	8	9	0.000	5.965	0.000	6.299	5.340	7.716	8.389	7.039	7.064	5.347	0.000	7.819	yes	yes			2
P06316	LV107_HUMAN	4	2	9	0.000	3.972	5.104	3.693	2.647	5.298	0.000	3.219	4.208	2.513	4.204	0.000					0
P18085	ARF4_HUMAN	5	2	9	4.014	5.776	3.904	4.911	0.000	4.876	2.683	5.818	0.000	3.530	0.000	2.551				yes	1
P62879	GBB2_HUMAN	5	6	9	5.070	5.643	5.239	0.000	0.000	4.978	5.751	5.112	4.312	0.000	5.084	5.481	yes	yes	yes		3
P21741	MK_HUMAN	5	8	9	6.446	7.426	8.458	0.000	6.858	8.101	6.844	7.615	4.759	7.159	0.000	0.000					0
Q95604	1C17_HUMAN	6	0	9	4.068	5.566	1.880	4.634	5.119	0.000	0.000	5.556	0.000	2.630	4.066	2.704		yes			1
P30044	PRDX5_HUMAN	6	6	9	3.134	5.982	5.449	6.083	0.000	0.000	5.768	6.107	3.918	4.984	0.000	6.437	yes		yes	yes	3
P62158	CALM_HUMAN	6	2	9	5.429	5.397	4.566	5.050	0.000	0.000	4.817	5.935	0.000	4.597	5.147	4.806	yes	yes		yes	3
P21926	CD9_HUMAN	6	8	9	6.049	6.193	0.000	6.718	0.000	6.361	6.910	6.906	5.105	6.567	0.000	7.333	yes	yes	yes	yes	4
Q9Y2Q3	GSTK1_HUMAN	6	2	9	0.000	4.090	3.791	4.283	3.901	5.052	3.818	6.495	0.000	3.841	0.000	4.994		yes		yes	2
P24844	MYL9_HUMAN	6	8	9	0.000	6.421	0.000	7.023	4.892	0.000	7.372	6.573	5.812	6.794	6.809	7.598	yes	yes	yes		3
P19013	K2C4_HUMAN	7	8	9	0.000	0.000	7.199	2.804	6.760	5.534	5.366	0.000	6.927	6.949	6.186	5.604		yes		yes	2
O15511	ARPC5_HUMAN	7	7	9	5.090	6.046	5.974	5.902	0.000	4.842	7.122	6.177	0.000	4.338	0.000	7.037			yes		1
O00264	PGRC1_HUMAN	8	2	9	3.375	0.000	0.000	4.196	4.405	5.220	4.751	5.370	4.452	3.903	0.000	5.894		yes	yes	yes	3
P48047	ATPO_HUMAN	8	5	9	0.000	5.745	0.000	4.559	4.434	5.558	5.982	6.679	0.000	3.751	4.027	7.503		yes	yes	yes	3
P60953	CDC42_HUMAN	8	7	9	0.000	7.439	6.706	4.045	0.000	7.002	7.404	7.525	7.392	4.872	0.000	7.746	yes		yes	yes	3
Q14847	LASP1_HUMAN	8	3	9	0.000	6.731	4.211	4.153	0.000	5.246	4.563	7.003	4.790	0.000	5.748	4.450			yes		1

P59998	ARPC4_HUMAN	8	9	9	5.541	6.753	6.143	6.528	0.000	5.781	6.856	6.829	0.000	5.850	0.000	7.059			yes	yes	2
Q03591	FHR1_HUMAN	8	5	9	5.524	7.003	0.000	5.050	6.983	5.651	0.000	2.834	6.216	2.928	0.000	4.355					0
P07360	CO8G_HUMAN	8	6	9	6.031	5.073	6.459	4.439	0.000	8.142	7.226	5.099	5.509	0.000	0.000	5.537					0
P10644	KAP0_HUMAN	9	1	9	3.794	5.568	3.704	4.901	4.569	4.413	0.000	5.110	0.000	5.698	5.279	0.000			yes		1
P11234	RALB_HUMAN	9	2	9	3.589	4.906	3.388	5.224	3.981	3.966	0.000	6.091	0.000	5.381	4.234	0.000			yes	yes	2
Q16643	DREB_HUMAN	10	3	9	4.847	6.605	3.868	4.664	4.679	4.433	0.000	7.140	0.000	5.187	4.873	0.000					0
O94919	ENDD1_HUMAN	11	6	9	0.000	5.920	4.433	6.017	0.000	0.000	5.285	6.545	5.243	4.406	7.423	6.133		yes	yes		2
P04040	CATA_HUMAN	11	4	9	4.074	6.682	5.825	3.960	4.105	0.000	0.000	6.647	0.000	5.445	4.426	3.773	yes	yes	yes		3
Q9Y6C2	EMIL1_HUMAN	11	5	9	3.265	5.321	3.603	0.000	0.000	0.000	6.614	5.751	4.153	5.722	6.512	6.675					0
P00739	HPTR_HUMAN	12	8	9	7.869	0.000	7.787	5.784	5.894	8.561	8.434	6.241	8.295	0.000	5.468	0.000	yes	yes			2
P24557	THAS_HUMAN	13	8	9	0.000	5.938	0.000	6.994	5.745	0.000	5.425	6.849	5.216	5.985	7.613	7.037			yes		1
Q06033	ITIH3_HUMAN	13	6	9	6.252	5.866	6.431	4.539	0.000	7.131	7.448	5.371	0.000	0.000	4.839	5.769					0
P00748	FA12_HUMAN	15	4	9	3.628	6.392	3.066	6.128	5.574	7.149	0.000	5.549	3.863	4.783	0.000	0.000					0
P28289	TMOD1_HUMAN	16	7	9	6.184	6.783	7.275	6.393	0.000	0.000	0.000	5.858	4.811	3.653	7.267	6.754					0
P03952	KLKB1_HUMAN	16	5	9	6.082	6.785	6.930	5.774	3.757	7.572	0.000	5.233	5.280	5.118	0.000	0.000					0
Q9UBW5	BIN2_HUMAN	17	8	9	6.601	7.913	6.117	6.715	6.092	6.431	0.000	8.560	0.000	6.542	4.151	0.000	yes	yes	yes		3
P0C0L4	CO4A_HUMAN	18	6	9	6.358	5.518	6.170	7.386	5.682	6.128	2.866	0.000	4.583	6.226	0.000	0.000	yes				1
P23229	ITA6_HUMAN	18	8	9	6.890	6.333	4.719	0.000	0.000	6.709	4.903	7.605	0.000	5.856	7.233	5.316	yes	yes	yes	yes	4
P07225	PROS_HUMAN	18	7	9	6.519	7.279	6.714	6.562	5.325	7.779	0.000	6.844	0.000	6.240	4.105	0.000	yes	yes	yes		3
P10643	CO7_HUMAN	19	6	9	6.587	5.906	6.173	6.968	0.000	6.851	3.555	6.338	3.953	3.625	0.000	0.000					0
P26038	MOES_HUMAN	21	9	9	6.346	9.068	6.109	7.372	7.071	6.059	0.000	8.570	0.000	7.125	6.476	0.000	yes	yes	yes	yes	4
Q05682	CALD1_HUMAN	21	5	9	3.533	7.361	3.732	7.607	6.735	0.000	0.000	7.988	0.000	6.164	5.790	5.044	yes	yes	yes		3
O75636	FCN3_HUMAN	23	7	9	7.522	9.533	7.790	0.000	3.697	9.133	6.873	0.000	9.713	0.000	4.784	6.582	yes	yes			2
P35612	ADDB_HUMAN	25	6	9	6.443	8.301	8.371	7.438	4.259	0.000	0.000	7.005	0.000	5.087	5.998	4.576					0
Q14766	LTBP1_HUMAN	28	6	9	4.767	7.074	3.291	0.000	0.000	3.770	8.692	7.155	0.000	6.087	7.096	6.887		yes	yes		2
P12236	ADT3_HUMAN	2	0	8	2.553	3.361	3.519	0.000	0.000	2.666	0.000	3.240	0.000	2.984	5.178	5.137		yes	yes	yes	3
Q71U36	TBA1A_HUMAN	2	1	8	3.107	3.245	0.000	0.000	4.747	0.000	3.830	4.963	2.666	6.299	5.443	0.000		yes		yes	2
P01620	KV302_HUMAN	2	1	8	5.640	2.841	1.894	4.603	3.028	0.000	0.000	4.670	0.000	4.266	3.001	0.000		yes			1
P05976	MYL1_HUMAN	2	8	8	7.011	6.326	6.858	5.781	0.000	7.054	5.717	7.135	0.000	0.000	0.000	7.137					0
P00403	COX2_HUMAN	6	3	8	0.000	4.899	4.348	5.333	0.000	4.383	4.436	5.907	4.020	0.000	0.000	6.655		yes	yes	yes	3
Q3ZCW2	LEGL_HUMAN	6	3	8	0.000	5.831	3.158	5.081	3.808	5.535	0.000	6.345	0.000	4.940	0.000	3.736	yes				1
P21796	VDAC1_HUMAN	6	7	8	0.000	6.449	0.000	0.000	4.434	5.092	6.267	6.415	5.624	0.000	6.333	6.125		yes	yes	yes	3
P07358	CO8B_HUMAN	6	1	8	4.649	0.000	4.110	5.094	0.000	4.973	5.029	0.000	4.416	3.701	0.000	4.127					0
P06744	G6PI_HUMAN	6	3	8	3.256	5.956	0.000	4.870	3.977	0.000	0.000	6.120	3.883	5.476	4.324	0.000	yes		yes	yes	3
O15117	FYB_HUMAN	7	6	8	5.284	5.654	4.718	0.000	0.000	5.427	5.607	6.429	0.000	4.959	0.000	4.272			yes		1
Q96AX2	RAB37_HUMAN	8	0	8	0.000	2.825	1.888	2.444	0.000	2.173	3.697	4.931	3.936	0.000	0.000	4.106					0
Q9Y251	HPSE_HUMAN	8	4	8	0.000	6.215	0.000	5.094	4.415	0.000	6.190	6.616	0.000	5.342	5.488	4.953		yes	yes		2
P19086	GNAZ_HUMAN	8	3	8	0.000	6.031	3.170	0.000	0.000	3.826	4.315	6.211	3.858	0.000	5.522	5.736		yes	yes		2
O15400	STX7_HUMAN	8	5	8	0.000	6.775	5.929	0.000	0.000	4.790	4.730	6.005	5.111	0.000	6.001	6.819			yes		1
P02750	A2GL_HUMAN	8	5	8	5.524	0.000	5.602	5.107	0.000	6.874	6.664	4.245	5.740	0.000	4.447	0.000					0
Q00325	MPCP_HUMAN	9	7	8	0.000	5.906	0.000	5.707	4.868	0.000	6.565	6.393	0.000	5.396	6.361	6.684	yes	yes		yes	3
P08238	HS90B_HUMAN	9	4	8	4.377	6.808	3.806	7.413	5.609	4.153	0.000	6.268	0.000	4.627	0.000	0.000			yes	yes	2
P30048	PRDX3_HUMAN	9	3	8	4.548	5.338	0.000	5.059	0.000	4.870	6.275	7.427	0.000	0.000	4.260	6.855		yes	yes	yes	3
Q14344	GNA13_HUMAN	9	4	8	4.029	5.999	0.000	0.000	5.710	0.000	4.648	6.685	4.427	0.000	5.299	5.227	yes	yes	yes	yes	4
P05452	TETN_HUMAN	9	3	8	6.725	0.000	0.000	4.022	4.542	8.079	4.427	5.501	4.550	0.000	0.000	5.260					0
Q15833	STXB2_HUMAN	10	3	8	0.000	7.061	0.000	4.849	3.745	0.000	4.375	7.169	0.000	5.821	5.628	5.054			yes		1
P52209	6PGD_HUMAN	10	3	8	0.000	6.287	0.000	5.894	4.833	3.508	4.085	7.009	0.000	0.000	5.236	3.708	yes		yes	yes	3
P08107	HSP71_HUMAN	11	4	8	0.000	6.937	0.000	4.959	5.869	2.197	4.044	6.955	0.000	6.292	3.773	0.000	yes	yes	yes	yes	4
P78417	GSTO1_HUMAN	11	6	8	5.515	5.865	5.353	6.079	0.000	4.996	0.000	7.465	0.000	5.184	0.000	5.158	yes	yes		yes	3
Q14247	SRC8_HUMAN	11	4	8	4.155	8.057	0.000	7.039	5.789	3.489	0.000	7.963	4.076	4.997	0.000	0.000			yes		1
P01137	TGFB1_HUMAN	11	4	8	0.000	5.852	0.000	4.587	3.849	0.000	6.820	5.956	4.529	0.000	4.928	6.959		yes	yes		2
O75390	CISY_HUMAN	11	4	8	0.000	5.729	0.000	5.382	4.262	3.414	4.239	6.286	0.000	0.000	5.591	5.844		yes	yes	yes	3
Q15555	MARE2_HUMAN	12	3	8	0.000	7.187	0.000	0.000	4.860	4.131	5.315	7.102	4.357	0.000	5.736	3.742	yes	yes	yes		3
P02538	K2C6A_HUMAN	13	3	8	4.984	2.896	0.000	3.759	4.330	6.497	0.000	0.000	7.247	0.000	3.311	4.714	yes	yes		yes	3
P50148	GNAQ_HUMAN	14	5	8	0.000	7.454	0.000	0.000	3.712	3.284	5.969	7.332	5.178	0.000	6.745	6.609	yes	yes	yes	yes	4
Q14520	HABP2_HUMAN	15	5	8	5.313	7.535	0.000	4.945	7.904	3.725	0.000	7.199	4.197	7.348	0.000	0.000					0
Q12913	PTPRJ_HUMAN	16	3	8	3.120	0.000	0.000	0.000	0.000	4.100	7.304	4.398	5.172	4.332	6.698	6.716	yes	yes	yes		3
P35858	ALS_HUMAN	16	4	8	6.165	7.115	6.753	4.866	3.770	7.782	0.000	5.096	5.070	0.000	0.000	0.000					0
P17301	ITA2_HUMAN	17	4	8	2.906	4.447	0.000	0.000	0.000	4.813	7.383	6.563	0.000	4.613	6.571	7.830		yes	yes	yes	3
P06681	CO2_HUMAN	17	5	8	6.700	6.504	5.716	4.933	3.781	7.521	0.000	5.752	0.000	3.896	0.000	0.000					0
P19367	HXK1_HUMAN	21	7	8	0.000	8.389	0.000	7.287	5.824	4.928	0.000	8.856	0.000	6.677	6.950	5.566	yes		yes	yes	3
Q14697	GANAB_HUMAN	21	4	8	4.668	7.364	4.086	6.798	0.000	4.215	0.000	8.270	0.000	5.845	0.000	3.962	yes		yes	yes	3

P02788	TRFL_HUMAN	28	7	8	6.708	7.168	7.657	3.960	8.006	6.804	0.000	7.666	0.000	7.177	0.000	0.000	yes				1
P68032	ACTC_HUMAN	1	0	7	3.098	4.528	0.000	0.000	0.000	4.660	3.668	4.408	0.000	0.000	4.872	4.790					0
P04434	KV310_HUMAN	1	0	7	3.673	0.000	4.422	4.778	3.871	0.000	0.000	5.050	2.812	2.455	0.000	0.000					0
P15153	RAC2_HUMAN	2	0	7	4.041	3.162	0.000	0.000	0.000	4.949	4.278	3.984	2.599	0.000	0.000	4.439	yes		yes	yes	3
Q12955	ANK3_HUMAN	2	5	7	5.534	0.000	5.999	0.000	0.000	0.000	6.634	3.403	5.822	4.608	0.000	6.825					0
P01825	HV207_HUMAN	2	3	7	3.994	0.000	4.716	5.009	5.305	5.592	6.147	0.000	4.724	0.000	0.000	0.000					0
P0CG47	UBB_HUMAN	2	4	7	5.148	6.098	4.957	0.000	0.000	0.000	0.000	5.451	0.000	4.872	5.743	6.213	yes			yes	2
P04430	KV122_HUMAN	2	5	7	6.880	0.000	5.981	5.971	4.384	7.205	0.000	5.840	0.000	4.799	0.000	0.000					0
P62834	RAP1A_HUMAN	3	0	7	0.000	3.901	0.000	3.584	0.000	0.000	2.392	4.070	2.920	2.463	0.000	3.613				yes	1
Q15836	VAMP3_HUMAN	3	3	7	4.609	5.557	4.555	0.000	0.000	5.665	6.946	5.126	0.000	0.000	0.000	6.683					0
Q9NX76	CKLF6_HUMAN	3	4	7	5.039	4.997	0.000	0.000	0.000	6.274	6.446	5.223	0.000	3.771	0.000	6.629				yes	1
P04431	KV123_HUMAN	4	0	7	0.000	0.000	2.865	4.145	2.831	3.115	0.000	5.393	0.000	3.857	4.793	0.000					0
P37840	SYUA_HUMAN	4	5	7	5.637	5.762	6.990	5.731	0.000	0.000	0.000	6.376	0.000	3.474	0.000	4.524			yes		1
P69892	HBG2_HUMAN	4	1	7	3.642	0.000	3.286	5.144	0.000	2.769	4.007	6.254	0.000	0.000	0.000	2.279					0
P13501	CCL5_HUMAN	5	5	7	0.000	5.506	5.175	0.000	0.000	5.207	6.963	5.545	6.228	0.000	0.000	6.610			yes		1
P13987	CD59_HUMAN	5	4	7	0.000	6.916	0.000	5.260	4.631	0.000	0.000	5.999	5.427	0.000	5.491	5.967	yes			yes	2
P35232	PHB_HUMAN	5	4	7	5.453	0.000	4.219	4.657	0.000	0.000	6.539	4.994	5.404	0.000	0.000	6.247		yes	yes	yes	3
P08185	CBG_HUMAN	5	3	7	5.174	0.000	5.517	0.000	0.000	5.487	0.000	5.279	5.376	0.000	4.523	3.820		yes			1
Q13103	SPP24_HUMAN	5	2	7	4.178	0.000	0.000	0.000	5.699	6.100	3.638	5.157	4.460	4.258	0.000	0.000					0
Q9UL25	RAB21_HUMAN	6	0	7	0.000	3.112	0.000	3.590	4.605	0.000	4.001	5.345	0.000	5.071	0.000	3.724					0
P30040	ERP29_HUMAN	6	3	7	4.095	4.604	4.661	3.583	0.000	0.000	5.799	5.930	0.000	0.000	0.000	5.345			yes	yes	2
P07339	CATD_HUMAN	6	0	7	3.704	0.000	0.000	0.000	4.338	3.374	3.704	4.556	3.691	0.000	0.000	4.393			yes	yes	2
P20340	RAB6A_HUMAN	7	2	7	0.000	2.909	0.000	2.742	0.000	4.410	3.905	6.197	0.000	2.676	0.000	5.262		yes		yes	2
Q13637	RAB32_HUMAN	7	1	7	0.000	4.509	0.000	4.316	3.954	0.000	3.469	6.049	0.000	3.763	0.000	4.965		yes	yes		2
Q0ZGT2	NEXN_HUMAN	7	3	7	4.275	5.890	3.769	5.872	4.921	0.000	0.000	6.330	0.000	0.000	0.000	4.775	yes				1
Q86YW5	TRML1_HUMAN	7	4	7	0.000	5.483	0.000	4.723	3.702	5.305	6.626	6.644	0.000	0.000	0.000	6.876	yes		yes		2
P22352	GPX3_HUMAN	7	4	7	5.656	0.000	6.368	0.000	0.000	6.614	5.179	0.000	5.410	0.000	4.010	5.020					0
P23219	PGH1_HUMAN	8	2	7	0.000	6.245	0.000	5.112	3.771	0.000	0.000	6.672	0.000	5.279	4.725	5.089	yes	yes	yes		3
Q15691	MARE1_HUMAN	8	2	7	3.212	5.232	3.214	4.259	0.000	0.000	5.500	6.346	0.000	0.000	4.154	0.000	yes		yes		2
Q14141	SEPT6_HUMAN	8	3	7	0.000	4.607	0.000	5.605	4.947	0.000	0.000	5.678	0.000	5.280	5.875	3.477					0
Q96AG4	LRC59_HUMAN	9	2	7	0.000	5.268	4.040	0.000	0.000	4.716	5.185	6.036	3.593	0.000	0.000	5.453			yes		1
Q9UKX3	MYH13_HUMAN	10	6	7	0.000	0.000	0.000	0.000	0.000	4.407	9.100	5.634	8.318	7.376	7.233	8.165					0
O15143	ARC1B_HUMAN	10	4	7	0.000	6.568	0.000	0.000	0.000	3.440	4.580	6.631	4.828	0.000	6.077	6.025	yes	yes	yes		3
Q16181	SEPT7_HUMAN	11	1	7	0.000	4.954	0.000	6.019	0.000	4.283	0.000	4.982	0.000	3.641	5.284	3.538		yes			1
Q27J81	INF2_HUMAN	12	2	7	3.500	4.960	0.000	0.000	0.000	3.315	5.950	3.943	4.569	0.000	0.000	6.223					0
P09486	SPRC_HUMAN	12	4	7	0.000	6.706	0.000	5.480	4.265	0.000	4.682	6.597	0.000	0.000	6.061	4.664	yes	yes	yes	yes	4
O15144	ARPC2_HUMAN	12	5	7	0.000	7.026	0.000	5.100	5.777	0.000	4.453	7.572	0.000	6.046	6.407	0.000		yes	yes	yes	3
O75083	WDR1_HUMAN	13	4	7	0.000	7.036	0.000	5.844	4.341	3.360	0.000	7.632	0.000	6.309	0.000	4.264	yes	yes	yes	yes	4
P07948	LYN_HUMAN	13	3	7	0.000	5.304	0.000	5.953	4.654	0.000	0.000	5.569	0.000	5.369	6.840	4.515			yes		1
P16109	LYAM3_HUMAN	14	5	7	0.000	4.508	0.000	0.000	0.000	3.862	6.603	6.631	0.000	5.927	6.046	6.533	yes	yes	yes		3
Q9NYU2	UGGG1_HUMAN	14	2	7	0.000	4.810	3.894	0.000	0.000	0.000	6.352	5.013	0.000	3.549	5.766	6.390			yes	yes	2
O00429	DNM1L_HUMAN	15	3	7	3.145	7.674	2.966	5.827	4.955	0.000	0.000	7.272	3.787	0.000	0.000	0.000	yes			yes	2
Q70J99	UN13D_HUMAN	16	3	7	3.830	7.297	0.000	5.113	0.000	3.359	0.000	7.765	0.000	6.074	5.291	0.000			yes		1
Q14644	RASA3_HUMAN	20	2	7	4.101	6.917	0.000	5.074	3.833	4.410	0.000	7.839	0.000	4.732	0.000	0.000			yes		1
P07384	CAN1_HUMAN	24	5	7	0.000	8.307	3.992	7.278	6.008	0.000	0.000	7.738	0.000	6.436	4.202	0.000	yes	yes	yes	yes	4
Q13576	IQGA2_HUMAN	32	5	7	0.000	4.349	0.000	0.000	0.000	3.289	7.456	5.905	0.000	5.525	7.147	7.072	yes		yes		2
Q7RTS7	K2C74_HUMAN	1	3	6	0.000	4.587	4.272	0.000	5.530	5.004	0.000	0.000	6.731	0.000	3.247	0.000					0
P01612	KV120_HUMAN	1	1	6	4.326	0.000	4.547	4.159	0.000	5.732	0.000	3.615	0.000	3.017	0.000	0.000					0
P18464	1B51_HUMAN	2	0	6	3.110	2.957	0.000	0.000	3.714	3.248	2.593	0.000	0.000	0.000	4.377	0.000					0
Q92930	RAB8B_HUMAN	2	1	6	0.000	3.001	0.000	3.077	0.000	4.776	0.000	4.635	0.000	0.000	3.015	5.241				yes	1
P01624	KV306_HUMAN	2	0	6	3.336	0.000	3.211	0.000	0.000	4.069	3.789	0.000	2.948	0.000	0.000	2.767					0
P35580	MYH10_HUMAN	2	1	6	2.311	0.000	2.175	0.000	0.000	2.460	5.472	4.673	0.000	0.000	0.000	3.531			yes	yes	2
P08493	MGP_HUMAN	2	2	6	0.000	0.000	0.000	0.000	5.082	5.357	6.903	4.581	5.192	0.000	0.000	5.005					0
Q9BWP8	COL11_HUMAN	2	1	6	0.000	4.435	4.644	0.000	0.000	4.138	4.815	4.155	5.797	0.000	0.000	0.000	yes				1
P04220	MUCB_HUMAN	3	5	6	7.633	7.614	0.000	0.000	0.000	5.984	5.290	0.000	5.020	0.000	6.306	0.000	yes				1
P01611	KV119_HUMAN	3	3	6	0.000	4.426	7.301	5.948	0.000	7.134	0.000	5.551	0.000	5.086	0.000	0.000		yes			1
P25325	THTM_HUMAN	3	0	6	0.000	5.033	3.117	0.000	0.000	3.662	4.406	4.167	0.000	0.000	0.000	3.667					0
Q9P0L0	VAPA_HUMAN	3	0	6	0.000	4.362	0.000	4.830	0.000	0.000	4.738	4.877	0.000	0.000	5.339	5.040					0
P14174	MIF_HUMAN	3	2	6	3.911	6.307	4.521	0.000	0.000	0.000	5.799	5.608	0.000	0.000	0.000	5.106				yes	1
P61088	UBE2N_HUMAN	3	2	6	5.243	6.142	4.401	0.000	0.000	4.781	0.000	5.478	0.000	0.000	0.000	4.588			yes		1
P61020	RAB5B_HUMAN	4	1	6	0.000	0.000	5.327	3.729	0.000	3.657	2.910	3.234	0.000	0.000	0.000	4.969				yes	1
Q9BVK6	TMED9_HUMAN	4	0	6	0.000	0.000	0.000	0.000	0.000	0.000	3.617	5.373	3.881	4.232	4.089	4.187				yes	1

P81605	DCD_HUMAN	4	3	6	4.689	5.254	0.000	4.328	0.000	6.949	0.000	0.000	7.935	0.000	0.000	6.509	yes				1
Q9H299	SH3L3_HUMAN	4	4	6	5.319	5.473	5.942	0.000	0.000	5.943	0.000	4.189	0.000	0.000	0.000	6.741				yes	1
P62805	H4_HUMAN	4	4	6	5.743	6.502	0.000	0.000	5.504	4.428	0.000	3.917	5.624	0.000	0.000	0.000				yes	1
P55056	APOC4_HUMAN	4	2	6	6.937	4.602	0.000	3.549	4.342	5.105	0.000	4.865	0.000	0.000	0.000	0.000	yes	yes			2
P61626	LYSC_HUMAN	4	6	6	7.716	0.000	7.562	0.000	0.000	8.625	6.859	0.000	5.697	0.000	0.000	6.962	yes	yes			2
P01877	IGHA2_HUMAN	5	2	6	0.000	4.556	0.000	0.000	0.000	0.000	5.400	7.959	3.708	0.000	4.207	4.268					0
Q9NR31	SAR1A_HUMAN	5	3	6	0.000	5.268	0.000	5.933	0.000	4.809	0.000	6.404	0.000	4.695	0.000	5.290			yes		1
O95197	RTN3_HUMAN	5	0	6	0.000	0.000	0.000	0.000	0.000	0.000	3.923	4.970	4.047	3.477	4.113	4.248		yes			1
P51572	BAP31_HUMAN	5	3	6	3.964	0.000	3.535	5.829	0.000	4.181	0.000	6.282	0.000	0.000	0.000	5.395					0
P52565	GDIR1_HUMAN	5	0	6	3.020	5.122	2.892	0.000	0.000	3.239	0.000	5.572	0.000	0.000	0.000	3.543	yes	yes		yes	3
P61769	B2MG_HUMAN	5	4	6	0.000	6.140	0.000	0.000	0.000	4.914	6.737	6.052	4.284	0.000	0.000	6.088	yes			yes	2
P21291	CSRP1_HUMAN	6	2	6	0.000	3.947	0.000	5.249	0.000	5.526	3.613	6.204	0.000	0.000	0.000	4.906	yes		yes	yes	3
P36542	ATPG_HUMAN	6	3	6	0.000	6.140	0.000	0.000	4.266	0.000	5.452	6.225	4.416	0.000	0.000	5.141			yes	yes	2
Q14165	MLEC_HUMAN	6	3	6	0.000	5.911	0.000	0.000	0.000	0.000	5.584	6.092	4.091	0.000	4.947	4.817		yes	yes	yes	3
P26641	EF1G_HUMAN	6	1	6	0.000	0.000	0.000	5.745	0.000	3.393	0.000	5.193	0.000	3.935	4.309	3.691				yes	1
P06311	KV311_HUMAN	6	3	6	6.706	0.000	5.157	0.000	2.826	6.723	4.290	4.621	0.000	0.000	0.000	0.000		yes			1
P09104	ENOG_HUMAN	7	1	6	0.000	5.331	1.937	5.173	0.000	2.264	0.000	5.743	0.000	0.000	4.216	0.000	yes		yes		2
P29622	KAIN_HUMAN	7	1	6	3.813	0.000	4.454	0.000	4.258	5.528	0.000	0.000	5.184	3.880	0.000	0.000					0
P45880	VDAC2_HUMAN	7	2	6	0.000	6.295	0.000	0.000	0.000	3.460	4.810	5.690	0.000	0.000	4.248	3.617		yes	yes	yes	3
Q16762	THTR_HUMAN	7	2	6	0.000	5.775	0.000	3.467	0.000	3.078	0.000	6.132	0.000	0.000	4.832	3.538			yes		1
O75947	ATP5H_HUMAN	7	3	6	0.000	5.237	3.315	0.000	0.000	4.831	5.538	5.883	0.000	0.000	0.000	6.850		yes	yes	yes	3
P13804	ETFA_HUMAN	8	1	6	0.000	5.630	0.000	0.000	0.000	3.295	5.017	5.375	0.000	0.000	4.281	4.920			yes	yes	2
Q13162	PRDX4_HUMAN	8	4	6	0.000	5.645	0.000	5.894	4.960	0.000	0.000	6.836	0.000	5.734	0.000	4.479		yes	yes	yes	3
Q99798	ACON_HUMAN	8	1	6	3.971	6.061	0.000	4.706	4.025	3.967	0.000	5.232	0.000	0.000	0.000	0.000			yes	yes	2
P50990	TCPQ_HUMAN	8	2	6	0.000	4.326	0.000	3.859	3.969	0.000	0.000	5.792	0.000	4.273	5.886	0.000			yes	yes	2
P55209	NP1L1_HUMAN	8	0	6	0.000	5.504	0.000	5.130	4.584	0.000	0.000	5.231	0.000	4.242	4.623	0.000	yes	yes	yes	yes	4
O75563	SKAP2_HUMAN	8	0	6	0.000	4.113	0.000	5.126	5.170	0.000	0.000	4.198	4.703	4.875	0.000	0.000	yes				1
P62140	PP1B_HUMAN	9	2	6	0.000	6.702	3.793	0.000	0.000	0.000	0.000	6.898	4.846	0.000	4.019	3.913					0
P07355	ANXA2_HUMAN	9	3	6	5.051	4.835	5.275	0.000	0.000	6.247	0.000	4.945	4.007	0.000	0.000	0.000		yes		yes	2
Q8N474	SFRP1_HUMAN	9	6	6	6.830	6.712	5.743	0.000	7.230	0.000	0.000	6.441	0.000	5.769	0.000	0.000					0
P05160	F13B_HUMAN	9	3	6	6.291	4.196	5.281	4.326	5.142	6.422	0.000	0.000	0.000	0.000	0.000	0.000					0
Q15019	SEPT2_HUMAN	10	4	6	0.000	6.718	0.000	0.000	0.000	0.000	5.111	7.007	4.533	0.000	5.969	5.409				yes	1
O95445	APOM_HUMAN	10	4	6	5.373	0.000	6.201	0.000	0.000	7.433	4.178	0.000	0.000	3.791	0.000	5.580	yes				1
Q9BSJ8	ESYT1_HUMAN	12	3	6	0.000	5.373	0.000	0.000	0.000	0.000	5.300	6.651	0.000	3.649	6.060	4.607	yes	yes			2
Q01813	K6PP_HUMAN	12	3	6	3.869	6.657	5.862	4.760	0.000	3.610	0.000	6.353	0.000	0.000	0.000	0.000	yes		yes	yes	3
Q13642	FHL1_HUMAN	12	4	6	0.000	7.348	0.000	5.910	4.713	0.000	4.498	7.697	0.000	0.000	6.145	0.000			yes		1
Q13045	FLII_HUMAN	13	2	6	3.231	0.000	0.000	0.000	0.000	3.484	5.299	4.073	0.000	4.429	0.000	6.632			yes		1
P22314	UBA1_HUMAN	13	3	6	4.722	7.363	3.143	5.497	0.000	0.000	0.000	7.889	0.000	3.653	0.000	0.000	yes		yes	yes	3
P23142	FBLN1_HUMAN	13	6	6	6.034	5.592	5.404	5.769	6.215	6.351	0.000	0.000	0.000	0.000	0.000	0.000		yes			1
P80108	PHLD_HUMAN	18	1	6	4.486	5.208	0.000	4.342	0.000	7.564	0.000	4.818	0.000	0.000	4.099	0.000					0
P18577	RHCE_HUMAN	2	4	5	0.000	5.958	0.000	6.400	0.000	0.000	0.000	0.000	4.891	0.000	6.335	6.961					0
P15814	IGLL1_HUMAN	2	0	5	4.631	0.000	1.825	0.000	0.000	4.888	2.615	0.000	3.721	0.000	0.000	0.000					0
P84085	ARF5_HUMAN	3	0	5	4.012	3.957	0.000	0.000	0.000	4.358	0.000	2.816	0.000	0.000	0.000	4.585					0
Q562R1	ACTBL_HUMAN	3	0	5	0.000	3.897	0.000	3.498	3.653	0.000	2.512	0.000	0.000	0.000	0.000	2.370	yes	yes			2
P63092	GNAS2_HUMAN	3	0	5	0.000	3.938	0.000	0.000	0.000	0.000	0.000	3.158	2.928	0.000	3.067	3.614					0
P62491	RB11A_HUMAN	3	0	5	0.000	0.000	0.000	0.000	3.510	0.000	4.437	3.898	0.000	3.710	0.000	4.872		yes		yes	2
P01617	KV204_HUMAN	3	1	5	3.084	0.000	4.737	2.474	0.000	4.010	0.000	0.000	2.754	0.000	0.000	0.000					0
P20339	RAB5A_HUMAN	3	0	5	3.215	0.000	2.141	0.000	0.000	3.901	0.000	3.099	0.000	0.000	0.000	4.032				yes	1
P05534	1A24_HUMAN	3	0	5	0.000	4.892	2.006	0.000	4.614	0.000	0.000	0.000	3.779	0.000	4.888	0.000		yes			1
Q8IZP2	ST134_HUMAN	3	0	5	0.000	4.226	3.064	0.000	0.000	0.000	0.000	4.189	0.000	2.587	4.917	0.000					0
Q15363	TMED2_HUMAN	3	1	5	0.000	4.180	4.099	0.000	0.000	0.000	5.248	4.000	0.000	0.000	0.000	6.746			yes	yes	2
O00483	NDUA4_HUMAN	3	2	5	0.000	5.054	2.805	0.000	0.000	3.283	6.899	0.000	0.000	0.000	0.000	6.556					0
O14745	NHRF1_HUMAN	3	0	5	0.000	4.062	0.000	3.690	0.000	3.646	0.000	4.603	0.000	3.744	0.000	0.000					0
Q96DZ9	CKLF5_HUMAN	3	2	5	0.000	5.682	0.000	3.635	0.000	0.000	6.456	5.352	4.399	0.000	0.000	0.000		yes			1
P04921	GLPC_HUMAN	3	4	5	6.967	6.744	7.613	0.000	0.000	0.000	5.228	0.000	0.000	0.000	0.000	7.460					0
P01601	KV109_HUMAN	3	2	5	6.549	0.000	0.000	5.134	5.469	0.000	0.000	0.000	0.000	4.848	3.130	0.000					0
P11177	ODPB_HUMAN	4	0	5	0.000	5.182	0.000	0.000	0.000	4.017	4.250	3.886	0.000	0.000	0.000	4.256			yes	yes	2
Q5SQ64	LY66F_HUMAN	4	0	5	0.000	5.474	0.000	0.000	0.000	0.000	4.237	5.416	0.000	0.000	4.821	3.801					0
O14773	TPP1_HUMAN	4	1	5	0.000	4.721	0.000	4.680	0.000	0.000	0.000	5.629	0.000	0.000	4.109	3.732				yes	1
P62330	ARF6_HUMAN	4	0	5	3.618	5.343	4.090	0.000	0.000	4.647	0.000	4.645	0.000	0.000	0.000	0.000					0
Q9NVA2	SEP11_HUMAN	4	0	5	0.000	0.000	0.000	4.661	3.732	0.000	0.000	5.230	0.000	4.152	4.248	0.000	yes				1
P15169	CBPN_HUMAN	4	1	5	3.005	0.000	3.721	0.000	0.000	5.533	0.000	0.000	4.266	3.897	0.000	0.000					0

P01112	RASH_HUMAN	5	1	5	0.000	2.984	5.024	0.000	0.000	3.275	0.000	4.509	0.000	0.000	0.000	4.244				yes	1
Q9NY65	TBA8_HUMAN	5	1	5	3.281	0.000	0.000	0.000	4.005	0.000	0.000	4.418	2.666	0.000	6.302	0.000	yes		yes		2
P62136	PP1A_HUMAN	5	0	5	0.000	5.471	0.000	0.000	0.000	3.427	0.000	5.323	3.806	0.000	0.000	4.432					0
P61923	COPZ1_HUMAN	5	0	5	0.000	3.812	0.000	0.000	0.000	3.792	4.155	3.900	0.000	0.000	0.000	4.930					0
Q15746	MYLK_HUMAN	5	1	5	0.000	5.166	0.000	4.774	4.358	0.000	0.000	6.016	0.000	4.717	0.000	0.000			yes		1
P14314	GLU2B_HUMAN	5	3	5	0.000	5.968	0.000	5.888	4.981	0.000	0.000	6.739	0.000	3.855	0.000	0.000			yes	yes	2
P62826	RAN_HUMAN	5	2	5	0.000	3.879	0.000	4.295	4.423	0.000	0.000	5.747	0.000	5.485	0.000	0.000	yes			yes	2
P48426	PI42A_HUMAN	5	1	5	0.000	5.508	0.000	5.019	0.000	0.000	0.000	6.101	0.000	4.465	4.982	0.000	yes				1
P46109	CRKL_HUMAN	5	0	5	3.724	5.006	3.100	0.000	0.000	4.029	0.000	5.602	0.000	0.000	0.000	0.000	yes		yes		2
P51884	LUM_HUMAN	5	4	5	6.133	0.000	6.091	0.000	0.000	7.142	0.000	5.147	5.532	0.000	0.000	0.000					0
Q16836	HCDH_HUMAN	6	1	5	0.000	6.322	0.000	0.000	0.000	0.000	3.756	4.174	0.000	0.000	4.265	4.922		yes	yes	yes	3
Q99685	MGLL_HUMAN	6	1	5	4.187	5.516	0.000	0.000	0.000	4.775	4.341	5.877	0.000	0.000	0.000	0.000			yes		1
P63241	IF5A1_HUMAN	6	1	5	4.313	5.516	0.000	0.000	0.000	0.000	4.932	5.726	3.870	0.000	0.000	0.000				yes	1
P61019	RAB2A_HUMAN	7	1	5	0.000	4.743	0.000	0.000	0.000	4.551	4.174	6.456	0.000	0.000	0.000	5.102				yes	1
Q9NP72	RAB18_HUMAN	7	2	5	0.000	5.408	0.000	0.000	0.000	4.463	3.702	6.309	0.000	0.000	0.000	5.695					0
O43488	ARK72_HUMAN	7	2	5	0.000	6.211	0.000	0.000	0.000	4.385	4.864	6.369	0.000	0.000	0.000	5.122			yes		1
P40925	MDHC_HUMAN	7	3	5	0.000	6.153	3.965	0.000	0.000	4.965	4.364	5.815	0.000	0.000	0.000	0.000	yes		yes	yes	3
P49411	EFTU_HUMAN	7	1	5	0.000	4.100	0.000	4.343	0.000	0.000	0.000	4.099	0.000	0.000	5.059	5.305			yes	yes	2
P50402	EMD_HUMAN	8	3	5	0.000	6.471	0.000	0.000	0.000	4.818	5.626	5.742	0.000	0.000	0.000	4.674			yes		1
Q13011	ECH1_HUMAN	8	3	5	0.000	6.060	0.000	3.792	0.000	0.000	5.946	5.820	0.000	0.000	5.783	0.000		yes		yes	2
Q14974	IMB1_HUMAN	8	3	5	3.943	5.853	0.000	5.737	0.000	0.000	0.000	6.727	0.000	4.912	0.000	0.000			yes	yes	2
P14923	PLAK_HUMAN	8	2	5	3.142	0.000	5.344	0.000	3.755	5.901	0.000	0.000	0.000	3.900	0.000	0.000		yes			1
P24539	AT5F1_HUMAN	9	1	5	0.000	0.000	0.000	4.520	0.000	0.000	5.094	5.336	0.000	0.000	4.664	5.909				yes	1
P08758	ANXA5_HUMAN	9	2	5	0.000	6.733	0.000	0.000	0.000	3.927	0.000	6.041	0.000	0.000	4.091	4.697	yes		yes	yes	3
Q6IBS0	TWF2_HUMAN	9	2	5	0.000	6.152	0.000	3.514	0.000	0.000	0.000	6.036	0.000	0.000	5.206	3.706		yes	yes		2
Q99623	PHB2_HUMAN	9	4	5	0.000	6.211	0.000	0.000	4.223	0.000	5.455	6.348	0.000	0.000	5.880	0.000		yes	yes	yes	3
P13667	PDIA4_HUMAN	10	4	5	0.000	6.606	0.000	6.733	4.664	0.000	0.000	6.835	0.000	5.576	0.000	0.000	yes		yes	yes	3
O75116	ROCK2_HUMAN	11	0	5	0.000	0.000	0.000	0.000	0.000	0.000	4.765	4.574	0.000	3.715	5.322	4.870			yes		1
Q92954	PRG4_HUMAN	11	2	5	5.130	0.000	3.808	0.000	0.000	6.279	0.000	0.000	3.765	0.000	0.000	4.169	yes				1
P51659	DHB4_HUMAN	12	2	5	0.000	6.804	0.000	4.862	4.672	0.000	0.000	6.651	0.000	3.879	0.000	0.000			yes	yes	2
P34932	HSP74_HUMAN	12	2	5	3.554	6.091	3.575	3.804	0.000	0.000	0.000	6.752	0.000	0.000	0.000	0.000			yes	yes	2
P11216	PYGB_HUMAN	15	3	5	0.000	7.227	0.000	4.952	3.723	0.000	0.000	6.262	0.000	5.740	0.000	0.000	yes		yes		2
P15924	DESP_HUMAN	19	3	5	3.913	0.000	7.483	0.000	0.000	6.245	0.000	0.000	6.266	3.717	0.000	0.000		yes		yes	2
P20742	PZP_HUMAN	2	0	4	0.000	3.229	0.000	2.563	0.000	0.000	0.000	0.000	0.000	2.541	4.117	0.000	yes				1
P58546	MTPN_HUMAN	2	2	4	0.000	4.173	0.000	0.000	0.000	0.000	6.132	5.472	0.000	0.000	0.000	5.298			yes		1
P59665	DEF1_HUMAN	2	3	4	6.049	0.000	5.102	0.000	0.000	5.800	0.000	0.000	4.278	0.000	0.000	0.000	yes				1
P01770	HV309_HUMAN	2	0	4	0.000	0.000	4.565	4.315	4.065	0.000	0.000	0.000	3.959	0.000	0.000	0.000					0
P01880	IGHD_HUMAN	2	1	4	0.000	0.000	4.434	5.809	0.000	0.000	0.000	4.880	4.490	0.000	0.000	0.000					0
Q96E17	RAB3C_HUMAN	3	0	4	3.613	0.000	0.000	0.000	0.000	3.162	0.000	0.000	0.000	3.514	0.000	2.524					0
P13747	HLAE_HUMAN	3	0	4	0.000	0.000	2.174	0.000	0.000	2.577	0.000	3.970	4.045	0.000	0.000	0.000					0
P11233	RALA_HUMAN	3	0	4	0.000	2.814	0.000	0.000	0.000	0.000	0.000	3.995	0.000	4.209	0.000	4.013				yes	1
Q96KK5	H2A1H_HUMAN	3	1	4	0.000	3.430	3.888	0.000	0.000	5.724	0.000	0.000	0.000	3.592	0.000	0.000					0
P30273	FCERG_HUMAN	3	4	4	0.000	6.000	0.000	0.000	0.000	0.000	6.687	6.061	0.000	0.000	0.000	5.851					0
O14818	PSA7_HUMAN	3	0	4	0.000	3.829	0.000	4.438	0.000	0.000	0.000	4.879	0.000	0.000	0.000	3.974				yes	1
P13598	ICAM2_HUMAN	3	0	4	0.000	5.544	0.000	0.000	0.000	0.000	0.000	4.873	0.000	0.000	4.228	3.717		yes	yes	yes	3
P01614	KV201_HUMAN	3	2	4	3.070	0.000	4.890	0.000	0.000	5.238	0.000	3.054	0.000	0.000	0.000	0.000					0
P36980	FHR2_HUMAN	3	0	4	0.000	3.255	0.000	0.000	2.766	0.000	4.745	2.992	0.000	0.000	0.000	0.000					0
P22694	KAPCB_HUMAN	4	0	4	0.000	5.062	0.000	0.000	0.000	0.000	4.423	5.557	0.000	0.000	0.000	3.818			yes		1
P49721	PSB2_HUMAN	4	0	4	0.000	4.540	0.000	0.000	0.000	4.125	0.000	4.261	0.000	0.000	0.000	4.412			yes	yes	2
Q9HDC9	APMAP_HUMAN	4	0	4	0.000	0.000	0.000	4.775	0.000	4.750	0.000	4.606	0.000	0.000	0.000	4.225				yes	1
P28066	PSA5_HUMAN	4	0	4	0.000	3.875	0.000	3.782	0.000	0.000	0.000	5.443	0.000	5.111	0.000	0.000			yes	yes	2
P61604	CH10_HUMAN	4	2	4	0.000	4.153	0.000	0.000	0.000	0.000	5.546	0.000	0.000	0.000	4.177	6.030				yes	1
Q96FZ7	CHMP6_HUMAN	4	0	4	0.000	5.241	0.000	0.000	0.000	0.000	4.034	5.167	0.000	0.000	4.928	0.000					0
O75368	SH3L1_HUMAN	4	1	4	0.000	5.053	4.099	0.000	0.000	4.187	0.000	0.000	0.000	0.000	0.000	5.488	yes		yes		2
P30086	PEBP1_HUMAN	4	0	4	4.025	4.792	0.000	0.000	0.000	4.406	0.000	5.341	0.000	0.000	0.000	0.000			yes	yes	2
P37837	TALDO_HUMAN	4	0	4	0.000	5.506	0.000	0.000	0.000	3.384	0.000	5.344	0.000	0.000	4.940	0.000	yes			yes	2
P04278	SHBG_HUMAN	4	1	4	5.561	0.000	3.159	0.000	0.000	3.574	0.000	0.000	3.703	0.000	0.000	0.000					0
Q8WWA1	TMM40_HUMAN	5	1	4	0.000	0.000	0.000	0.000	0.000	3.314	4.417	4.817	0.000	0.000	0.000	6.306					0
O95866	G6B_HUMAN	5	1	4	0.000	5.421	0.000	0.000	0.000	0.000	4.873	5.345	0.000	0.000	0.000	6.176		yes			1
Q9Y394	DHRS7_HUMAN	5	0	4	0.000	4.939	0.000	0.000	0.000	0.000	4.473	4.794	0.000	0.000	0.000	3.920					0
Q9BV40	VAMP8_HUMAN	5	3	4	0.000	5.612	0.000	0.000	0.000	0.000	6.691	4.602	0.000	0.000	0.000	6.900			yes		1
Q99733	NP1L4_HUMAN	5	1	4	0.000	4.844	0.000	3.631	0.000	0.000	0.000	5.619	0.000	0.000	0.000	3.802				yes	1

Q9UJU6	DBNL_HUMAN	5	1	4	0.000	4.509	0.000	4.575	0.000	0.000	0.000	6.059	0.000	3.996	0.000	0.000					0
P11413	G6PD_HUMAN	5	0	4	0.000	4.493	0.000	4.529	0.000	0.000	0.000	4.886	0.000	3.723	0.000	0.000	yes		yes	yes	3
P51452	DUS3_HUMAN	5	3	4	0.000	5.811	0.000	4.201	0.000	5.067	0.000	6.041	0.000	0.000	0.000	0.000	yes		yes		2
Q53GQ0	DHB12_HUMAN	5	0	4	0.000	0.000	0.000	0.000	0.000	0.000	4.710	3.929	0.000	0.000	4.232	4.180		yes			1
Q06323	PSME1_HUMAN	5	1	4	3.287	5.464	0.000	0.000	0.000	0.000	3.862	5.678	0.000	0.000	0.000	0.000		yes		yes	2
Q12907	LMAN2_HUMAN	5	0	4	0.000	5.548	0.000	0.000	0.000	0.000	3.773	5.566	0.000	0.000	4.443	0.000				yes	1
P25787	PSA2_HUMAN	5	0	4	0.000	3.839	4.677	0.000	0.000	0.000	3.611	4.084	0.000	0.000	0.000	0.000			yes	yes	2
Q9H8H3	MET7A_HUMAN	5	0	4	0.000	0.000	3.088	0.000	0.000	0.000	0.000	5.157	0.000	0.000	4.027	4.483		yes			1
O43294	TGFI1_HUMAN	5	0	4	0.000	4.204	0.000	4.550	0.000	0.000	0.000	4.822	0.000	0.000	4.160	0.000	yes				1
Q6DD88	ATLA3_HUMAN	5	0	4	0.000	3.886	0.000	4.409	0.000	0.000	0.000	4.080	0.000	0.000	4.590	0.000					0
P07357	CO8A_HUMAN	5	0	4	3.609	0.000	3.804	0.000	0.000	4.877	0.000	0.000	4.277	0.000	0.000	0.000					0
P60981	DEST_HUMAN	5	2	4	4.353	5.803	3.008	0.000	0.000	0.000	0.000	6.045	0.000	0.000	0.000	0.000			yes	yes	2
P54819	KAD2_HUMAN	6	0	4	0.000	0.000	0.000	0.000	0.000	0.000	3.562	5.026	0.000	3.548	0.000	5.104			yes		1
P18669	PGAM1_HUMAN	6	1	4	0.000	0.000	0.000	4.792	0.000	0.000	0.000	5.757	0.000	4.434	0.000	4.085			yes	yes	2
Q8NBX0	SCPDH_HUMAN	6	0	4	0.000	5.117	0.000	4.161	0.000	0.000	0.000	5.209	0.000	0.000	0.000	4.509				yes	1
P55145	MANF_HUMAN	6	2	4	0.000	4.955	0.000	0.000	0.000	0.000	5.463	5.832	0.000	3.521	0.000	0.000			yes	yes	2
P39656	OST48_HUMAN	6	0	4	0.000	0.000	0.000	0.000	0.000	0.000	4.760	5.465	0.000	0.000	4.139	4.050		yes	yes	yes	3
P05067	A4_HUMAN	6	1	4	0.000	5.322	0.000	0.000	0.000	0.000	0.000	6.089	0.000	0.000	4.725	3.576					0
Q16698	DECR_HUMAN	6	1	4	0.000	4.648	0.000	0.000	0.000	0.000	3.723	5.768	0.000	0.000	4.704	0.000			yes	yes	2
Q9BWM7	SFXN3_HUMAN	6	0	4	0.000	4.871	0.000	0.000	0.000	0.000	4.927	4.672	0.000	0.000	4.898	0.000			yes	yes	2
Q13423	NNTM_HUMAN	6	0	4	0.000	5.061	0.000	4.255	0.000	0.000	0.000	5.379	0.000	0.000	4.294	0.000		yes	yes	yes	3
P55058	PLTP_HUMAN	6	2	4	4.192	0.000	6.112	4.159	0.000	5.071	0.000	0.000	0.000	0.000	0.000	0.000					0
Q5D862	FILA2_HUMAN	6	2	4	0.000	0.000	6.477	0.000	4.317	0.000	0.000	0.000	7.289	0.000	0.000	4.569		yes			1
Q9UFN0	NPS3A_HUMAN	7	2	4	0.000	0.000	0.000	0.000	0.000	0.000	4.145	5.987	0.000	4.577	0.000	5.621		yes		yes	2
P31150	GDIA_HUMAN	7	2	4	0.000	5.847	0.000	4.623	0.000	0.000	0.000	6.118	0.000	4.582	0.000	0.000	yes		yes	yes	3
P43304	GPDM_HUMAN	7	0	4	0.000	5.271	0.000	3.868	0.000	0.000	0.000	5.078	0.000	4.860	0.000	0.000		yes	yes	yes	3
P22695	QCR2_HUMAN	7	1	4	0.000	0.000	0.000	5.070	0.000	0.000	0.000	6.357	0.000	0.000	5.249	3.930		yes	yes	yes	3
Q06187	BTK_HUMAN	7	0	4	3.059	5.439	0.000	0.000	0.000	0.000	0.000	5.099	0.000	3.676	0.000	0.000			yes		1
P55084	ECHB_HUMAN	7	2	4	0.000	0.000	0.000	5.386	3.725	0.000	0.000	5.809	0.000	0.000	5.574	0.000			yes	yes	2
O60610	DIAP1_HUMAN	8	2	4	0.000	0.000	0.000	0.000	0.000	0.000	6.631	5.869	0.000	3.747	0.000	4.856			yes		1
Q7L576	CYFP1_HUMAN	8	1	4	0.000	0.000	0.000	0.000	0.000	3.913	4.748	0.000	0.000	4.002	0.000	5.197			yes		1
Q9UHQ9	NB5R1_HUMAN	8	1	4	0.000	6.258	0.000	0.000	0.000	0.000	5.198	5.487	0.000	0.000	4.322	0.000			yes		1
P22792	CPN2_HUMAN	8	3	4	6.003	5.247	5.405	0.000	0.000	7.179	0.000	0.000	0.000	0.000	0.000	0.000					0
Q05209	PTN12_HUMAN	9	3	4	0.000	6.103	0.000	5.503	0.000	0.000	3.837	7.069	0.000	0.000	0.000	0.000			yes	yes	2
Q9BUL8	PDC10_HUMAN	9	2	4	0.000	4.615	0.000	5.510	0.000	0.000	0.000	6.224	0.000	3.727	0.000	0.000					0
Q01082	SPTB2_HUMAN	10	1	4	0.000	0.000	0.000	0.000	0.000	0.000	6.417	3.947	0.000	2.707	3.152	0.000	yes		yes	yes	3
Q05193	DYN1_HUMAN	10	1	4	2.937	4.743	0.000	4.694	0.000	0.000	0.000	6.598	0.000	0.000	0.000	0.000			yes		1
P14543	NID1_HUMAN	11	2	4	0.000	0.000	0.000	0.000	0.000	0.000	5.667	0.000	0.000	3.801	4.782	6.372			yes		1
O76074	PDE5A_HUMAN	21	3	4	0.000	6.955	0.000	5.383	0.000	0.000	0.000	7.711	0.000	4.711	0.000	0.000			yes		1
Q86Y46	K2C73_HUMAN	1	0	3	0.000	0.000	1.860	0.000	0.000	4.807	0.000	0.000	0.000	0.000	0.000	3.095		yes			1
Q8N1N4	K2C78_HUMAN	2	0	3	0.000	0.000	4.054	0.000	0.000	0.000	0.000	0.000	4.053	3.608	0.000	0.000		yes			1
P11217	PYGM_HUMAN	2	0	3	0.000	3.008	0.000	0.000	0.000	0.000	0.000	3.175	3.664	0.000	0.000	0.000					0
Q6S8J3	POTEE_HUMAN	2	0	3	0.000	3.113	0.000	0.000	0.000	0.000	0.000	2.956	0.000	0.000	3.168	0.000					0
P20338	RAB4A_HUMAN	2	0	3	0.000	2.869	0.000	0.000	0.000	0.000	0.000	4.081	0.000	0.000	0.000	3.697					0
P10301	RRAS_HUMAN	2	0	3	0.000	0.000	0.000	0.000	0.000	4.835	0.000	4.950	0.000	0.000	0.000	4.443				yes	1
Q9H4G4	GAPR1_HUMAN	2	1	3	0.000	5.128	0.000	0.000	0.000	0.000	0.000	4.227	0.000	0.000	0.000	5.240	yes				1
Q8NHG7	SVIP_HUMAN	2	1	3	0.000	4.695	4.488	0.000	0.000	0.000	0.000	0.000	0.000	0.000	0.000	5.839					0
P17858	K6PL_HUMAN	3	0	3	0.000	4.986	0.000	2.810	0.000	0.000	0.000	4.413	0.000	0.000	0.000	0.000			yes		1
P12882	MYH1_HUMAN	3	0	3	0.000	0.000	0.000	0.000	0.000	0.000	4.044	0.000	3.075	3.676	0.000	0.000					0
P10619	PPGB_HUMAN	3	0	3	0.000	0.000	0.000	3.568	0.000	0.000	0.000	4.358	0.000	0.000	0.000	4.512					0
P06858	LIPL_HUMAN	3	0	3	3.211	0.000	0.000	0.000	3.935	0.000	0.000	0.000	0.000	0.000	4.197	0.000					0
Q16718	NDUA5_HUMAN	3	1	3	0.000	0.000	0.000	0.000	0.000	3.330	5.868	0.000	0.000	0.000	0.000	4.674				yes	1
P22234	PUR6_HUMAN	3	0	3	0.000	4.025	0.000	4.012	0.000	0.000	0.000	5.329	0.000	0.000	0.000	0.000			yes	yes	2
P23526	SAHH_HUMAN	3	0	3	0.000	4.242	0.000	4.119	0.000	0.000	0.000	4.342	0.000	0.000	0.000	0.000			yes	yes	2
P56134	ATPK_HUMAN	3	2	3	2.981	0.000	0.000	0.000	0.000	0.000	6.386	0.000	0.000	0.000	0.000	6.410				yes	1
Q9NR12	PDLI7_HUMAN	3	0	3	0.000	0.000	0.000	4.399	0.000	0.000	0.000	0.000	0.000	3.658	5.038	0.000					0
Q9H4X1	RGC32_HUMAN	3	0	3	3.009	0.000	4.442	0.000	0.000	0.000	0.000	3.975	0.000	0.000	0.000	0.000					0
Q13561	DCTN2_HUMAN	3	0	3	0.000	0.000	0.000	3.772	0.000	0.000	0.000	4.275	0.000	0.000	4.343	0.000				yes	1
Q13790	APOF_HUMAN	3	3	3	5.079	0.000	5.964	0.000	0.000	6.689	0.000	0.000	0.000	0.000	0.000	0.000	yes	yes			2
P30043	BLVRB_HUMAN	3	1	3	0.000	0.000	4.507	0.000	0.000	5.170	0.000	0.000	0.000	0.000	0.000	3.586			yes		1
P01719	LV501_HUMAN	3	1	3	4.417	0.000	5.321	0.000	0.000	0.000	4.548	0.000	0.000	0.000	0.000	0.000					0
O95292	VAPB_HUMAN	4	0	3	0.000	0.000	0.000	2.436	0.000	0.000	0.000	3.096	0.000	0.000	0.000	3.547					0

P84095	RHOG_HUMAN	4	0	3	0.000	4.585	0.000	0.000	0.000	3.859	0.000	2.813	0.000	0.000	0.000	0.000					0
Q9Y3A3	PHOCN_HUMAN	4	0	3	0.000	0.000	0.000	0.000	0.000	0.000	4.451	3.957	0.000	0.000	0.000	4.428					0
P49720	PSB3_HUMAN	4	1	3	0.000	0.000	0.000	0.000	0.000	0.000	0.000	5.630	0.000	3.530	0.000	5.069			yes	yes	2
Q12846	STX4_HUMAN	4	0	3	0.000	4.381	0.000	0.000	0.000	0.000	0.000	4.641	0.000	0.000	0.000	4.301					0
Q9H3N1	TMX1_HUMAN	4	0	3	0.000	4.161	0.000	0.000	0.000	0.000	0.000	4.071	0.000	0.000	0.000	3.505				yes	1
P29692	EF1D_HUMAN	4	0	3	0.000	4.242	0.000	0.000	0.000	0.000	4.464	5.207	0.000	0.000	0.000	0.000				yes	1
O75964	ATP5L_HUMAN	4	2	3	0.000	4.125	0.000	0.000	0.000	0.000	6.519	0.000	0.000	0.000	0.000	5.559		yes		yes	2
Q8N392	RHG18_HUMAN	4	0	3	0.000	4.358	0.000	3.744	0.000	0.000	0.000	4.718	0.000	0.000	0.000	0.000					0
P49588	SYAC_HUMAN	4	0	3	0.000	5.512	0.000	3.767	0.000	0.000	0.000	4.686	0.000	0.000	0.000	0.000			yes		1
P13073	COX41_HUMAN	4	1	3	0.000	6.061	0.000	4.644	0.000	0.000	0.000	4.116	0.000	0.000	0.000	0.000		yes		yes	2
Q99497	PARK7_HUMAN	4	2	3	0.000	0.000	0.000	4.097	0.000	4.970	0.000	5.998	0.000	0.000	0.000	0.000	yes		yes	yes	3
P20618	PSB1_HUMAN	4	0	3	0.000	0.000	3.777	0.000	0.000	3.293	0.000	5.244	0.000	0.000	0.000	0.000			yes	yes	2
P10124	SRGN_HUMAN	4	2	3	0.000	0.000	0.000	0.000	0.000	0.000	5.336	0.000	6.143	0.000	5.500	0.000		yes			1
P00505	AATM_HUMAN	4	0	3	0.000	4.881	0.000	0.000	0.000	0.000	0.000	5.202	0.000	0.000	4.367	0.000			yes	yes	2
Q9NTJ5	SAC1_HUMAN	4	0	3	0.000	0.000	0.000	0.000	3.745	0.000	0.000	4.859	0.000	0.000	5.101	0.000	yes	yes			2
Q16851	UGPA_HUMAN	4	0	3	0.000	0.000	0.000	0.000	0.000	0.000	0.000	4.485	0.000	3.838	4.185	0.000			yes		1
Q8N699	MYCT1_HUMAN	4	0	3	0.000	5.061	0.000	0.000	0.000	0.000	0.000	4.913	0.000	0.000	4.220	0.000					0
P50851	LRBA_HUMAN	4	0	3	0.000	0.000	0.000	0.000	0.000	0.000	3.822	0.000	0.000	0.000	4.963	3.914					0
O00391	QSOX1_HUMAN	4	1	3	5.160	0.000	4.360	0.000	0.000	3.999	0.000	0.000	0.000	0.000	0.000	0.000					0
P00492	HPRT_HUMAN	5	1	3	0.000	0.000	0.000	0.000	0.000	0.000	0.000	5.739	0.000	3.818	0.000	4.477			yes		1
Q9NVJ2	ARL8B_HUMAN	5	2	3	0.000	5.745	0.000	0.000	0.000	0.000	5.357	5.120	0.000	0.000	0.000	0.000					0
P11310	ACADM_HUMAN	5	0	3	0.000	4.763	0.000	3.798	0.000	0.000	0.000	5.181	0.000	0.000	0.000	0.000					0
Q8WUM4	PDC6I_HUMAN	5	1	3	0.000	5.699	0.000	3.684	0.000	0.000	0.000	4.782	0.000	0.000	0.000	0.000			yes	yes	2
P25788	PSA3_HUMAN	5	0	3	0.000	0.000	0.000	3.551	0.000	3.366	0.000	5.050	0.000	0.000	0.000	0.000				yes	1
P62714	PP2AB_HUMAN	5	0	3	0.000	4.900	0.000	0.000	0.000	4.034	0.000	5.065	0.000	0.000	0.000	0.000					0
P20930	FILA_HUMAN	5	0	3	3.503	0.000	0.000	0.000	0.000	3.435	0.000	0.000	4.794	0.000	0.000	0.000		yes			1
P35613	BASI_HUMAN	5	0	3	0.000	0.000	0.000	0.000	0.000	0.000	0.000	4.083	0.000	0.000	5.601	3.784	yes			yes	2
P31323	KAP3_HUMAN	5	0	3	0.000	5.206	0.000	0.000	0.000	0.000	0.000	4.927	0.000	0.000	4.274	0.000				yes	1
Q9H223	EHD4_HUMAN	5	0	3	0.000	4.398	0.000	0.000	4.649	0.000	0.000	3.786	0.000	0.000	0.000	0.000	yes		yes	yes	3
P13716	HEM2_HUMAN	5	2	3	0.000	5.652	0.000	0.000	0.000	0.000	0.000	5.710	0.000	0.000	4.290	0.000			yes		1
P06276	CHLE_HUMAN	5	2	3	5.385	0.000	3.136	0.000	0.000	6.109	0.000	0.000	0.000	0.000	0.000	0.000					0
O75882	ATRN_HUMAN	5	1	3	3.517	0.000	0.000	0.000	0.000	5.875	4.489	0.000	0.000	0.000	0.000	0.000					0
Q13586	STIM1_HUMAN	6	1	3	0.000	5.455	0.000	4.516	0.000	0.000	0.000	6.521	0.000	0.000	0.000	0.000		yes	yes		2
Q9H939	PPIP2_HUMAN	6	1	3	0.000	6.735	0.000	0.000	0.000	0.000	3.661	4.501	0.000	0.000	0.000	0.000			yes		1
P21964	COMT_HUMAN	6	0	3	0.000	0.000	3.514	0.000	0.000	0.000	0.000	3.988	0.000	0.000	0.000	4.908				yes	1
P12429	ANXA3_HUMAN	6	0	3	0.000	4.785	0.000	0.000	0.000	3.538	0.000	5.600	0.000	0.000	0.000	0.000	yes		yes	yes	3
P38646	GRP75_HUMAN	6	2	3	0.000	6.136	0.000	0.000	0.000	0.000	0.000	5.964	0.000	4.737	0.000	0.000		yes	yes	yes	3
Q9BYE4	SPR2G_HUMAN	6	2	3	2.969	0.000	0.000	0.000	0.000	6.272	0.000	0.000	5.809	0.000	0.000	0.000					0
P68104	EF1A1_HUMAN	6	1	3	4.885	0.000	0.000	0.000	0.000	3.810	0.000	0.000	0.000	0.000	5.129	0.000				yes	1
Q9UIJ7	KAD3_HUMAN	7	1	3	0.000	3.932	0.000	0.000	0.000	0.000	0.000	5.129	0.000	0.000	0.000	5.838				yes	1
P60842	IF4A1_HUMAN	7	0	3	0.000	4.841	0.000	3.510	0.000	0.000	0.000	5.561	0.000	0.000	0.000	0.000	yes			yes	2
P62993	GRB2_HUMAN	7	1	3	0.000	0.000	0.000	3.881	0.000	0.000	0.000	5.870	0.000	4.353	0.000	0.000	yes		yes		2
P02786	TFR1_HUMAN	7	1	3	0.000	0.000	0.000	0.000	0.000	5.621	4.313	0.000	0.000	4.034	0.000	0.000	yes			yes	2
Q9Y613	FHOD1_HUMAN	7	1	3	0.000	0.000	0.000	0.000	0.000	0.000	5.528	0.000	0.000	0.000	4.871	4.701			yes		1
P05543	THBG_HUMAN	7	0	3	4.617	0.000	4.266	0.000	0.000	4.624	0.000	0.000	0.000	0.000	0.000	0.000					0
Q9H4M9	EHD1_HUMAN	8	1	3	0.000	3.367	0.000	0.000	4.378	0.000	0.000	6.005	0.000	0.000	0.000	0.000	yes		yes	yes	3
P35237	SPB6_HUMAN	8	1	3	0.000	5.571	0.000	0.000	0.000	0.000	0.000	6.166	0.000	0.000	4.044	0.000			yes	yes	2
Q9Y696	CLIC4_HUMAN	8	1	3	0.000	0.000	4.627	4.113	0.000	0.000	0.000	6.016	0.000	0.000	0.000	0.000			yes		1
Q99714	HCD2_HUMAN	9	2	3	0.000	0.000	0.000	0.000	0.000	0.000	3.701	5.736	0.000	0.000	0.000	5.339		yes		yes	2
Q9Y4L1	HYOU1_HUMAN	9	2	3	0.000	0.000	0.000	0.000	0.000	0.000	6.299	5.331	0.000	0.000	0.000	6.059			yes	yes	2
P13489	RINI_HUMAN	9	1	3	0.000	4.071	0.000	5.008	0.000	0.000	0.000	6.075	0.000	0.000	0.000	0.000			yes	yes	2
Q9Y4D1	DAAM1_HUMAN	9	2	3	0.000	5.677	0.000	0.000	0.000	0.000	0.000	6.357	0.000	0.000	5.262	0.000					0
P41226	UBA7_HUMAN	11	2	3	0.000	6.737	0.000	4.137	0.000	0.000	0.000	7.665	0.000	0.000	0.000	0.000			yes		1
Q10567	AP1B1_HUMAN	11	2	3	0.000	5.863	0.000	0.000	0.000	0.000	0.000	6.635	0.000	4.540	0.000	0.000				yes	1
P53396	ACLY_HUMAN	12	2	3	0.000	6.056	0.000	0.000	0.000	0.000	0.000	5.685	0.000	4.291	0.000	0.000				yes	1
Q16610	ECM1_HUMAN	28	2	3	0.000	7.909	0.000	0.000	10.224	4.201	0.000	0.000	0.000	0.000	0.000	0.000		yes			1
P01700	LV102_HUMAN	1	0	2	0.000	0.000	0.000	0.000	0.000	0.000	2.878	0.000	0.000	0.000	0.000	2.647					0
Q6KB66	K2C80_HUMAN	2	0	2	0.000	0.000	4.435	0.000	0.000	0.000	0.000	0.000	2.741	0.000	0.000	0.000					0
Q96BM9	ARL8A_HUMAN	2	0	2	0.000	3.944	0.000	0.000	0.000	0.000	0.000	3.101	0.000	0.000	0.000	0.000					0
P09972	ALDOC_HUMAN	2	0	2	0.000	4.214	0.000	0.000	0.000	0.000	0.000	3.091	0.000	0.000	0.000	0.000	yes	yes	yes	yes	4
P01615	KV202_HUMAN	2	0	2	0.000	0.000	3.577	0.000	0.000	2.119	0.000	0.000	0.000	0.000	0.000	0.000					0
P08754	GNAI3_HUMAN	2	0	2	0.000	3.418	3.177	0.000	0.000	0.000	0.000	0.000	0.000	0.000	0.000	0.000					0

P25789	PSA4_HUMAN	2	0	2	3.716	0.000	4.567	0.000	0.000	0.000	0.000	0.000	0.000	0.000	0.000	0.000				yes	1
Q14008	CKAP5_HUMAN	2	0	2	0.000	0.000	0.000	0.000	0.000	0.000	4.785	0.000	0.000	0.000	0.000	4.163					0
P05089	ARGI1_HUMAN	2	0	2	0.000	0.000	4.505	0.000	0.000	0.000	0.000	0.000	4.550	0.000	0.000	0.000		yes			1
P26447	S10A4_HUMAN	2	0	2	0.000	4.493	0.000	0.000	0.000	0.000	0.000	5.414	0.000	0.000	0.000	0.000	yes				1
Q15185	TEBP_HUMAN	2	0	2	0.000	3.850	0.000	0.000	0.000	0.000	0.000	4.595	0.000	0.000	0.000	0.000			yes		1
Q9NTK5	OLA1_HUMAN	2	0	2	0.000	0.000	0.000	4.280	0.000	0.000	0.000	5.105	0.000	0.000	0.000	0.000				yes	1
Q99828	CIB1_HUMAN	2	0	2	0.000	0.000	0.000	0.000	0.000	3.370	0.000	0.000	0.000	0.000	0.000	4.076					0
P24666	PPAC_HUMAN	2	0	2	0.000	3.961	0.000	0.000	0.000	0.000	0.000	4.099	0.000	0.000	0.000	0.000			yes		1
P56377	AP1S2_HUMAN	2	0	2	0.000	4.591	0.000	0.000	0.000	0.000	0.000	4.737	0.000	0.000	0.000	0.000					0
P51571	SSRD_HUMAN	2	0	2	0.000	4.239	0.000	0.000	0.000	0.000	0.000	4.258	0.000	0.000	0.000	0.000				yes	1
O60518	RNBP6_HUMAN	2	0	2	0.000	4.105	0.000	0.000	0.000	0.000	0.000	4.071	0.000	0.000	0.000	0.000					0
Q15257	PTPA_HUMAN	2	0	2	0.000	4.327	0.000	0.000	0.000	0.000	0.000	4.281	0.000	0.000	0.000	0.000					0
P00441	SODC_HUMAN	2	0	2	0.000	4.745	0.000	0.000	0.000	0.000	0.000	4.653	0.000	0.000	0.000	0.000	yes			yes	2
P09543	CN37_HUMAN	2	0	2	0.000	3.971	0.000	0.000	0.000	0.000	0.000	3.873	0.000	0.000	0.000	0.000					0
Q9H2U2	IPYR2_HUMAN	2	0	2	0.000	4.892	0.000	0.000	0.000	0.000	0.000	4.144	0.000	0.000	0.000	0.000				yes	1
O00187	MASP2_HUMAN	2	0	2	0.000	0.000	0.000	0.000	0.000	4.653	0.000	0.000	0.000	0.000	0.000	3.631					0
P59768	GBG2_HUMAN	2	0	2	0.000	0.000	4.136	0.000	0.000	3.920	0.000	0.000	0.000	0.000	0.000	0.000					0
Q02094	RHAG_HUMAN	2	0	2	0.000	3.929	0.000	0.000	0.000	0.000	0.000	0.000	0.000	0.000	5.342	0.000					0
P01033	TIMP1_HUMAN	2	0	2	0.000	4.003	0.000	0.000	0.000	0.000	3.610	0.000	0.000	0.000	0.000	0.000					0
B2RUZ4	YA047_HUMAN	2	1	2	0.000	0.000	2.957	0.000	0.000	0.000	0.000	0.000	0.000	0.000	0.000	5.462					0
P01609	KV117_HUMAN	2	0	2	3.525	0.000	0.000	0.000	0.000	3.152	0.000	0.000	0.000	0.000	0.000	0.000					0
O43665	RGS10_HUMAN	2	0	2	0.000	0.000	3.904	0.000	0.000	4.665	0.000	0.000	0.000	0.000	0.000	0.000			yes		1
Q15762	CD226_HUMAN	2	0	2	0.000	4.799	0.000	0.000	0.000	0.000	0.000	0.000	0.000	4.540	0.000	0.000	yes	yes			2
P63218	GBG5_HUMAN	2	0	2	4.116	0.000	0.000	0.000	0.000	0.000	0.000	0.000	0.000	0.000	0.000	5.103					0
O75531	BAF_HUMAN	2	0	2	0.000	0.000	4.063	0.000	0.000	0.000	0.000	0.000	0.000	0.000	0.000	4.572				yes	1
P35443	TSP4_HUMAN	3	0	2	0.000	0.000	0.000	0.000	0.000	0.000	0.000	0.000	4.636	0.000	3.342	0.000					0
P37235	HPCL1_HUMAN	3	0	2	0.000	0.000	0.000	0.000	0.000	0.000	3.732	4.557	0.000	0.000	0.000	0.000					0
Q96A26	F162A_HUMAN	3	1	2	0.000	0.000	0.000	0.000	0.000	0.000	5.430	4.026	0.000	0.000	0.000	0.000			yes		1
P31930	QCR1_HUMAN	3	0	2	0.000	0.000	0.000	0.000	0.000	0.000	3.718	0.000	0.000	0.000	0.000	3.708			yes	yes	2
Q96AP7	ESAM_HUMAN	3	0	2	0.000	4.111	0.000	0.000	0.000	0.000	0.000	5.432	0.000	0.000	0.000	0.000				yes	1
P29350	PTN6_HUMAN	3	1	2	0.000	4.705	0.000	0.000	0.000	0.000	0.000	5.667	0.000	0.000	0.000	0.000	yes		yes		2
Q96FW1	OTUB1_HUMAN	3	0	2	0.000	4.021	0.000	0.000	0.000	0.000	0.000	4.687	0.000	0.000	0.000	0.000				yes	1
P36959	GMPR1_HUMAN	3	0	2	0.000	3.930	0.000	0.000	0.000	0.000	0.000	4.523	0.000	0.000	0.000	0.000			yes		1
P30153	2AAA_HUMAN	3	0	2	0.000	3.934	0.000	0.000	0.000	0.000	0.000	4.461	0.000	0.000	0.000	0.000			yes	yes	2
Q9Y6W5	WASF2_HUMAN	3	0	2	0.000	4.668	0.000	0.000	0.000	0.000	0.000	5.241	0.000	0.000	0.000	0.000					0
Q15819	UB2V2_HUMAN	3	0	2	0.000	4.451	0.000	0.000	0.000	0.000	0.000	4.939	0.000	0.000	0.000	0.000					0
Q8NG06	TRI58_HUMAN	3	0	2	0.000	4.316	0.000	0.000	0.000	0.000	0.000	4.545	0.000	0.000	0.000	0.000			yes		1
Q9H479	FN3K_HUMAN	3	0	2	0.000	4.061	0.000	0.000	0.000	0.000	0.000	4.241	0.000	0.000	0.000	0.000			yes		1
Q9UBQ7	GRHPR_HUMAN	3	0	2	0.000	4.359	0.000	0.000	0.000	0.000	0.000	4.543	0.000	0.000	0.000	0.000			yes		1
P35813	PPM1A_HUMAN	3	0	2	0.000	4.250	0.000	0.000	0.000	0.000	0.000	4.351	0.000	0.000	0.000	0.000			yes		1
O60763	USO1_HUMAN	3	0	2	0.000	4.037	0.000	0.000	0.000	0.000	0.000	4.113	0.000	0.000	0.000	0.000			yes		1
O14579	COPE_HUMAN	3	0	2	0.000	4.715	0.000	0.000	0.000	0.000	0.000	4.705	0.000	0.000	0.000	0.000			yes		1
Q15382	RHEB_HUMAN	3	0	2	0.000	3.932	0.000	0.000	0.000	0.000	0.000	3.859	0.000	0.000	0.000	0.000			yes		1
P05198	IF2A_HUMAN	3	0	2	0.000	4.715	0.000	0.000	0.000	0.000	0.000	4.603	0.000	0.000	0.000	0.000				yes	1
P09525	ANXA4_HUMAN	3	0	2	0.000	4.709	0.000	0.000	0.000	0.000	0.000	4.533	0.000	0.000	0.000	0.000				yes	1
O43561	LAT_HUMAN	3	0	2	0.000	0.000	0.000	0.000	0.000	3.849	0.000	0.000	0.000	0.000	0.000	3.741					0
Q14677	EPN4_HUMAN	3	0	2	0.000	4.652	0.000	0.000	0.000	0.000	0.000	4.267	0.000	0.000	0.000	0.000					0
Q99969	RARR2_HUMAN	3	0	2	0.000	4.610	0.000	0.000	0.000	0.000	0.000	4.121	0.000	0.000	0.000	0.000					0
Q7Z434	MAVS_HUMAN	3	0	2	0.000	0.000	0.000	0.000	0.000	0.000	0.000	4.704	0.000	3.670	0.000	0.000					0
P30049	ATPD_HUMAN	3	1	2	0.000	3.991	0.000	0.000	0.000	0.000	0.000	0.000	0.000	0.000	0.000	5.203					0
P43251	BTD_HUMAN	3	0	2	0.000	0.000	3.851	0.000	0.000	4.548	0.000	0.000	0.000	0.000	0.000	0.000					0
P01040	CYTA_HUMAN	3	1	2	4.688	0.000	0.000	0.000	0.000	6.428	0.000	0.000	0.000	0.000	0.000	0.000		yes			1
O75298	RTN2_HUMAN	3	0	2	0.000	5.219	0.000	4.107	0.000	0.000	0.000	0.000	0.000	0.000	0.000	0.000					0
Q8N5K1	CISD2_HUMAN	3	2	2	0.000	0.000	4.696	0.000	0.000	0.000	0.000	0.000	0.000	0.000	0.000	5.909					0
P35914	HMGCL_HUMAN	3	0	2	0.000	0.000	0.000	0.000	0.000	0.000	0.000	4.754	0.000	0.000	4.354	0.000			yes		1
Q02978	M2OM_HUMAN	3	0	2	0.000	0.000	0.000	0.000	0.000	0.000	0.000	4.623	0.000	0.000	4.895	0.000		yes	yes	yes	3
P48740	MASP1_HUMAN	3	0	2	0.000	0.000	0.000	0.000	4.822	3.887	0.000	0.000	0.000	0.000	0.000	0.000	yes				1
Q15365	PCBP1_HUMAN	3	0	2	0.000	4.783	0.000	0.000	0.000	0.000	0.000	5.060	0.000	0.000	0.000	0.000	yes		yes	yes	3
P05109	S10A8_HUMAN	3	0	2	0.000	5.241	0.000	0.000	0.000	0.000	0.000	4.649	0.000	0.000	0.000	0.000	yes	yes			2
Q10469	MGAT2_HUMAN	3	0	2	0.000	0.000	0.000	0.000	3.657	0.000	0.000	3.816	0.000	0.000	0.000	0.000					0
Q9NZP8	C1RL_HUMAN	3	2	2	4.927	0.000	4.907	0.000	0.000	0.000	0.000	0.000	0.000	0.000	0.000	0.000					0
Q15848	ADIPO_HUMAN	3	0	2	0.000	0.000	0.000	0.000	0.000	3.906	3.703	0.000	0.000	0.000	0.000	0.000					0

Q04695	K1C17_HUMAN	4	1	2	5.955	0.000	0.000	0.000	0.000	0.000	0.000	0.000	2.539	0.000	0.000	0.000		yes			1
Q99832	TCPH_HUMAN	4	0	2	0.000	0.000	0.000	0.000	0.000	0.000	0.000	3.998	0.000	0.000	0.000	3.746			yes	yes	2
P51809	VAMP7_HUMAN	4	0	2	0.000	0.000	0.000	0.000	0.000	0.000	0.000	5.499	0.000	0.000	0.000	4.597			yes		1
Q5JSH3	WDR44_HUMAN	4	0	2	0.000	0.000	0.000	0.000	0.000	0.000	3.786	0.000	0.000	0.000	0.000	4.753					0
Q9UDY2	ZO2_HUMAN	4	0	2	0.000	0.000	0.000	0.000	0.000	0.000	3.742	0.000	0.000	0.000	0.000	4.661			yes		1
Q92619	HMHA1_HUMAN	4	0	2	0.000	0.000	0.000	0.000	0.000	0.000	4.542	0.000	0.000	0.000	0.000	4.754			yes		1
O43747	AP1G1_HUMAN	4	0	2	0.000	3.995	0.000	0.000	0.000	0.000	0.000	4.955	0.000	0.000	0.000	0.000					0
Q08188	TGM3_HUMAN	4	0	2	0.000	0.000	3.804	0.000	0.000	0.000	0.000	0.000	3.935	0.000	0.000	0.000					0
P50453	SPB9_HUMAN	4	0	2	0.000	4.086	0.000	0.000	0.000	0.000	0.000	4.666	0.000	0.000	0.000	0.000			yes	yes	2
P29401	TKT_HUMAN	4	0	2	0.000	5.314	0.000	0.000	0.000	0.000	0.000	5.562	0.000	0.000	0.000	0.000			yes	yes	2
Q16891	IMMT_HUMAN	4	0	2	0.000	4.014	0.000	0.000	0.000	0.000	0.000	3.941	0.000	0.000	0.000	0.000			yes	yes	2
P16152	CBR1_HUMAN	4	0	2	0.000	5.289	0.000	0.000	0.000	0.000	0.000	4.839	0.000	0.000	0.000	0.000			yes		1
P10768	ESTD_HUMAN	4	0	2	0.000	5.399	0.000	0.000	0.000	0.000	0.000	4.881	0.000	0.000	0.000	0.000			yes		1
P18054	LOX12_HUMAN	4	0	2	0.000	0.000	0.000	0.000	4.547	0.000	0.000	0.000	0.000	4.842	0.000	0.000	yes	yes	yes		3
Q14019	COTL1_HUMAN	4	1	2	0.000	5.636	0.000	0.000	0.000	0.000	0.000	4.406	0.000	0.000	0.000	0.000	yes	yes	yes	yes	4
P27918	PROP_HUMAN	4	0	2	0.000	0.000	0.000	0.000	0.000	0.000	0.000	0.000	3.769	0.000	4.823	0.000	yes				1
P07741	APT_HUMAN	4	0	2	0.000	0.000	0.000	0.000	0.000	4.263	0.000	4.398	0.000	0.000	0.000	0.000	yes		yes		2
Q04837	SSBP_HUMAN	4	0	2	0.000	4.557	0.000	0.000	0.000	0.000	4.871	0.000	0.000	0.000	0.000	0.000			yes	yes	2
P28070	PSB4_HUMAN	4	0	2	0.000	0.000	4.304	0.000	0.000	0.000	0.000	4.496	0.000	0.000	0.000	0.000			yes		1
Q96IY4	CBPB2_HUMAN	4	0	2	0.000	0.000	3.337	0.000	0.000	4.578	0.000	0.000	0.000	0.000	0.000	0.000					0
P12273	PIP_HUMAN	4	1	2	3.083	0.000	0.000	0.000	0.000	5.739	0.000	0.000	0.000	0.000	0.000	0.000					0
Q9H0E2	TOLIP_HUMAN	4	0	2	0.000	4.528	3.216	0.000	0.000	0.000	0.000	0.000	0.000	0.000	0.000	0.000					0
O43399	TPD54_HUMAN	4	0	2	0.000	0.000	0.000	0.000	0.000	0.000	0.000	4.617	0.000	0.000	4.109	0.000					0
P07954	FUMH_HUMAN	4	0	2	0.000	0.000	0.000	0.000	0.000	0.000	0.000	0.000	0.000	0.000	5.009	4.258			yes	yes	2
O60885	BRD4_HUMAN	4	0	2	0.000	4.477	0.000	0.000	0.000	0.000	0.000	4.440	0.000	0.000	0.000	0.000					0
Q6PJW8	CNST_HUMAN	5	1	2	0.000	4.280	0.000	0.000	0.000	0.000	0.000	6.229	0.000	0.000	0.000	0.000					0
P28482	MK01_HUMAN	5	0	2	0.000	3.899	0.000	0.000	0.000	0.000	0.000	5.420	0.000	0.000	0.000	0.000			yes		1
P19971	TYPH_HUMAN	5	0	2	0.000	0.000	0.000	3.855	0.000	0.000	0.000	5.448	0.000	0.000	0.000	0.000	yes		yes		2
P40939	ECHA_HUMAN	5	0	2	0.000	4.266	0.000	0.000	0.000	0.000	0.000	5.203	0.000	0.000	0.000	0.000		yes		yes	2
Q16799	RTN1_HUMAN	5	1	2	0.000	0.000	0.000	0.000	0.000	0.000	5.359	0.000	0.000	0.000	0.000	3.749					0
O95373	IPO7_HUMAN	5	0	2	0.000	5.104	0.000	0.000	0.000	0.000	0.000	5.259	0.000	0.000	0.000	0.000					0
Q9Y2A7	NCKP1_HUMAN	5	0	2	0.000	5.312	0.000	0.000	0.000	0.000	0.000	5.087	0.000	0.000	0.000	0.000					0
P22061	PIMT_HUMAN	5	0	2	0.000	0.000	0.000	0.000	0.000	0.000	0.000	5.408	0.000	3.601	0.000	0.000					0
Q9NUQ9	FA49B_HUMAN	5	0	2	0.000	5.058	0.000	0.000	0.000	0.000	0.000	3.930	0.000	0.000	0.000	0.000			yes		1
Q96C24	SYTL4_HUMAN	5	0	2	0.000	4.275	0.000	4.356	0.000	0.000	0.000	0.000	0.000	0.000	0.000	0.000			yes		1
P39060	COIA1_HUMAN	5	0	2	0.000	0.000	0.000	0.000	0.000	0.000	4.669	0.000	3.835	0.000	0.000	0.000					0
P23634	AT2B4_HUMAN	5	2	2	0.000	0.000	5.187	0.000	0.000	0.000	0.000	0.000	0.000	0.000	0.000	5.981					0
Q96MH6	TMM68_HUMAN	5	1	2	0.000	0.000	0.000	4.568	0.000	0.000	0.000	6.035	0.000	0.000	0.000	0.000					0
P02741	CRP_HUMAN	5	2	2	0.000	0.000	0.000	0.000	0.000	6.091	6.275	0.000	0.000	0.000	0.000	0.000					0
O95782	AP2A1_HUMAN	6	2	2	0.000	5.629	0.000	0.000	0.000	0.000	0.000	5.992	0.000	0.000	0.000	0.000			yes	yes	2
P50224	ST1A3_HUMAN	6	0	2	0.000	5.399	0.000	0.000	0.000	0.000	0.000	5.564	0.000	0.000	0.000	0.000	yes				1
O43182	RHG06_HUMAN	6	0	2	0.000	5.502	0.000	0.000	0.000	0.000	0.000	5.464	0.000	0.000	0.000	0.000					0
O95870	ABHGA_HUMAN	6	0	2	0.000	0.000	0.000	4.220	0.000	0.000	0.000	0.000	0.000	0.000	5.580	0.000			yes		1
P78371	TCPB_HUMAN	7	1	2	0.000	5.754	0.000	0.000	0.000	0.000	0.000	5.167	0.000	0.000	0.000	0.000			yes	yes	2
O00410	IPO5_HUMAN	7	1	2	0.000	5.709	0.000	0.000	0.000	0.000	0.000	5.075	0.000	0.000	0.000	0.000				yes	1
P05164	PERM_HUMAN	7	1	2	0.000	0.000	0.000	0.000	5.823	0.000	0.000	0.000	0.000	5.291	0.000	0.000	yes				1
Q8WWZ8	OIT3_HUMAN	7	1	2	0.000	4.269	0.000	0.000	6.849	0.000	0.000	0.000	0.000	0.000	0.000	0.000	yes	yes			2
P23276	KELL_HUMAN	7	2	2	0.000	5.803	5.026	0.000	0.000	0.000	0.000	0.000	0.000	0.000	0.000	0.000	yes				1
Q02413	DSG1_HUMAN	7	1	2	0.000	0.000	6.879	0.000	0.000	0.000	0.000	0.000	4.998	0.000	0.000	0.000		yes			1
Q7KZF4	SND1_HUMAN	8	2	2	0.000	6.143	0.000	0.000	0.000	0.000	0.000	6.677	0.000	0.000	0.000	0.000				yes	1
P15531	NDKA_HUMAN	8	0	2	0.000	5.245	0.000	0.000	0.000	0.000	0.000	4.471	0.000	0.000	0.000	0.000	yes		yes		2
Q08380	LG3BP_HUMAN	8	2	2	5.161	0.000	0.000	0.000	0.000	6.045	0.000	0.000	0.000	0.000	0.000	0.000	yes	yes			2
O00139	KIF2A_HUMAN	9	1	2	0.000	5.206	0.000	0.000	0.000	0.000	0.000	6.593	0.000	0.000	0.000	0.000			yes		1
P02735	SAA_HUMAN	9	1	2	0.000	0.000	0.000	0.000	0.000	0.000	9.282	0.000	0.000	0.000	0.000	4.255		yes			1
P31944	CASPE_HUMAN	10	2	2	0.000	0.000	5.316	0.000	0.000	0.000	0.000	0.000	7.298	0.000	0.000	0.000					0
P06737	PYGL_HUMAN	10	1	2	0.000	6.756	0.000	0.000	0.000	0.000	0.000	4.808	0.000	0.000	0.000	0.000			yes		1
O43490	PROM1_HUMAN	10	2	2	0.000	0.000	0.000	0.000	0.000	0.000	5.307	0.000	0.000	5.568	0.000	0.000					0
Q09666	AHNK_HUMAN	13	2	2	0.000	5.626	0.000	0.000	0.000	5.475	0.000	0.000	0.000	0.000	0.000	0.000				yes	1
P04083	ANXA1_HUMAN	13	1	2	4.446	0.000	0.000	0.000	0.000	7.377	0.000	0.000	0.000	0.000	0.000	0.000	yes	yes		yes	3
P08727	K1C19_HUMAN	14	1	2	0.000	0.000	0.000	0.000	0.000	7.155	0.000	0.000	4.285	0.000	0.000	0.000		yes			1
P13646	K1C13_HUMAN	17	2	2	0.000	0.000	0.000	0.000	0.000	7.268	0.000	0.000	8.374	0.000	0.000	0.000	yes	yes		yes	3
Q5W0A0	F194B_HUMAN	18	2	2	0.000	7.242	0.000	0.000	0.000	0.000	0.000	0.000	0.000	5.411	0.000	0.000					0

P01703	LV105_HUMAN	1	0	1	0.000	0.000	0.000	0.000	0.000	0.000	2.822	0.000	0.000	0.000	0.000	0.000					0
Q13526	PIN1_HUMAN	2	0	1	0.000	0.000	0.000	0.000	0.000	0.000	0.000	3.937	0.000	0.000	0.000	0.000					0
P09417	DHPR_HUMAN	2	0	1	0.000	0.000	0.000	0.000	0.000	0.000	0.000	4.040	0.000	0.000	0.000	0.000					0
P13693	TCTP_HUMAN	2	0	1	0.000	0.000	0.000	0.000	0.000	0.000	0.000	3.869	0.000	0.000	0.000	0.000			yes	yes	2
P55160	NCKPL_HUMAN	2	0	1	0.000	0.000	0.000	0.000	0.000	0.000	0.000	4.889	0.000	0.000	0.000	0.000					0
Q9NXH8	CI167_HUMAN	2	0	1	0.000	0.000	0.000	0.000	0.000	0.000	0.000	4.355	0.000	0.000	0.000	0.000			yes		1
Q9Y3B3	TMED7_HUMAN	2	0	1	0.000	0.000	0.000	0.000	0.000	0.000	0.000	4.468	0.000	0.000	0.000	0.000					0
Q02218	ODO1_HUMAN	2	0	1	0.000	0.000	0.000	0.000	0.000	0.000	0.000	3.991	0.000	0.000	0.000	0.000				yes	1
O14980	XPO1_HUMAN	2	0	1	0.000	0.000	0.000	0.000	0.000	0.000	0.000	3.943	0.000	0.000	0.000	0.000				yes	1
Q9UQ80	PA2G4_HUMAN	2	0	1	0.000	0.000	0.000	0.000	0.000	0.000	0.000	3.941	0.000	0.000	0.000	0.000			yes		1
Q8TEA8	DTD1_HUMAN	2	0	1	0.000	0.000	0.000	0.000	0.000	0.000	0.000	3.917	0.000	0.000	0.000	0.000					0
P09496	CLCA_HUMAN	2	0	1	0.000	0.000	0.000	0.000	0.000	0.000	0.000	3.909	0.000	0.000	0.000	0.000					0
P21281	VATB2_HUMAN	2	0	1	0.000	0.000	0.000	0.000	0.000	0.000	0.000	4.017	0.000	0.000	0.000	0.000			yes	yes	2
P20936	RASA1_HUMAN	2	0	1	0.000	0.000	0.000	0.000	0.000	0.000	0.000	4.681	0.000	0.000	0.000	0.000					0
P51153	RAB13_HUMAN	2	0	1	0.000	0.000	0.000	0.000	0.000	0.000	0.000	3.882	0.000	0.000	0.000	0.000			yes		1
O00231	PSD11_HUMAN	2	0	1	0.000	0.000	0.000	0.000	0.000	0.000	0.000	4.166	0.000	0.000	0.000	0.000			yes	yes	2
P05121	PAI1_HUMAN	2	0	1	0.000	0.000	0.000	0.000	0.000	0.000	0.000	3.994	0.000	0.000	0.000	0.000			yes	yes	2
P19404	NDUV2_HUMAN	2	0	1	0.000	0.000	0.000	0.000	0.000	0.000	0.000	4.047	0.000	0.000	0.000	0.000				yes	1
P23368	MAOM_HUMAN	2	0	1	0.000	0.000	0.000	0.000	0.000	0.000	0.000	5.054	0.000	0.000	0.000	0.000		yes	yes	yes	3
P00568	KAD1_HUMAN	2	0	1	0.000	0.000	0.000	0.000	0.000	0.000	0.000	3.917	0.000	0.000	0.000	0.000			yes		1
P21912	DHSB_HUMAN	2	0	1	0.000	0.000	0.000	0.000	0.000	0.000	0.000	4.058	0.000	0.000	0.000	0.000				yes	1
Q9NR28	DBLOH_HUMAN	2	0	1	0.000	0.000	0.000	0.000	0.000	0.000	0.000	4.017	0.000	0.000	0.000	0.000					0
P20674	COX5A_HUMAN	2	0	1	0.000	0.000	0.000	0.000	0.000	0.000	0.000	4.412	0.000	0.000	0.000	0.000		yes		yes	2
Q9BR76	COR1B_HUMAN	2	0	1	0.000	0.000	0.000	0.000	0.000	0.000	0.000	4.130	0.000	0.000	0.000	0.000			yes		1
P35606	COPB2_HUMAN	2	0	1	0.000	0.000	0.000	0.000	0.000	0.000	0.000	4.031	0.000	0.000	0.000	0.000			yes	yes	2
Q4KMQ2	ANO6_HUMAN	2	0	1	0.000	0.000	0.000	0.000	0.000	0.000	0.000	4.145	0.000	0.000	0.000	0.000					0
O95169	NDUB8_HUMAN	2	0	1	0.000	0.000	0.000	0.000	0.000	0.000	0.000	3.849	0.000	0.000	0.000	0.000					0
Q7LDG7	GRP2_HUMAN	2	0	1	0.000	0.000	0.000	0.000	0.000	0.000	0.000	4.964	0.000	0.000	0.000	0.000					0
P41250	SYG_HUMAN	2	0	1	0.000	0.000	0.000	0.000	0.000	0.000	0.000	4.814	0.000	0.000	0.000	0.000			yes	yes	2
P04844	RPN2_HUMAN	2	0	1	0.000	0.000	0.000	0.000	0.000	0.000	0.000	4.868	0.000	0.000	0.000	0.000		yes	yes	yes	3
Q6XQN6	PNCB_HUMAN	2	0	1	0.000	0.000	0.000	0.000	0.000	0.000	0.000	4.451	0.000	0.000	0.000	0.000					0
O75489	NDUS3_HUMAN	2	0	1	0.000	0.000	0.000	0.000	0.000	0.000	0.000	4.568	0.000	0.000	0.000	0.000				yes	1
Q9ULC4	MCTS1_HUMAN	2	0	1	0.000	0.000	0.000	0.000	0.000	0.000	0.000	4.449	0.000	0.000	0.000	0.000					0
Q99627	CSN8_HUMAN	2	0	1	0.000	0.000	0.000	0.000	0.000	0.000	0.000	4.108	0.000	0.000	0.000	0.000					0
P55786	PSA_HUMAN	2	0	1	0.000	0.000	0.000	0.000	0.000	0.000	0.000	4.191	0.000	0.000	0.000	0.000				yes	1
P14550	AK1A1_HUMAN	2	0	1	0.000	0.000	0.000	0.000	0.000	0.000	0.000	3.885	0.000	0.000	0.000	0.000					0
P40227	TCPZ_HUMAN	2	0	1	0.000	0.000	0.000	0.000	0.000	0.000	0.000	3.964	0.000	0.000	0.000	0.000			yes	yes	2
P50991	TCPD_HUMAN	2	0	1	0.000	0.000	0.000	0.000	0.000	0.000	0.000	4.362	0.000	0.000	0.000	0.000	yes			yes	2
O60493	SNX3_HUMAN	2	0	1	0.000	0.000	0.000	0.000	0.000	0.000	0.000	4.039	0.000	0.000	0.000	0.000					0
Q9NS28	RGS18_HUMAN	2	0	1	0.000	0.000	0.000	0.000	0.000	0.000	0.000	4.205	0.000	0.000	0.000	0.000					0
P05771	KPCB_HUMAN	2	0	1	0.000	0.000	0.000	0.000	0.000	0.000	0.000	4.386	0.000	0.000	0.000	0.000		yes	yes		2
P00367	DHE3_HUMAN	2	0	1	0.000	0.000	0.000	0.000	0.000	0.000	0.000	3.921	0.000	0.000	0.000	0.000				yes	1
Q5XKP0	QIL1_HUMAN	2	0	1	0.000	0.000	0.000	0.000	0.000	0.000	0.000	0.000	0.000	0.000	0.000	3.685					0
P30084	ECHM_HUMAN	2	0	1	0.000	0.000	0.000	0.000	0.000	0.000	0.000	0.000	0.000	0.000	0.000	3.607					0
O15551	CLD3_HUMAN	2	0	1	0.000	0.000	0.000	0.000	0.000	0.000	0.000	0.000	0.000	0.000	0.000	3.412					0
Q9HBH0	RHOF_HUMAN	2	0	1	0.000	0.000	0.000	0.000	0.000	0.000	0.000	0.000	0.000	0.000	0.000	3.851					0
Q6IAA8	LTOR1_HUMAN	2	1	1	0.000	0.000	0.000	0.000	0.000	0.000	0.000	0.000	0.000	0.000	0.000	5.370					0
P53671	LIMK2_HUMAN	2	0	1	0.000	0.000	0.000	0.000	0.000	0.000	0.000	0.000	0.000	0.000	0.000	4.432					0
P42892	ECE1_HUMAN	2	0	1	0.000	0.000	0.000	0.000	0.000	0.000	0.000	0.000	0.000	0.000	0.000	4.393				yes	1
O15260	SURF4_HUMAN	2	0	1	0.000	0.000	0.000	0.000	0.000	0.000	0.000	0.000	0.000	0.000	0.000	4.330				yes	1
P43307	SSRA_HUMAN	2	0	1	0.000	0.000	0.000	0.000	0.000	0.000	0.000	0.000	0.000	0.000	0.000	3.713				yes	1
Q15477	SKIV2_HUMAN	2	0	1	0.000	0.000	0.000	0.000	0.000	0.000	0.000	0.000	0.000	0.000	0.000	3.924					0
Q9UJC5	SH3L2_HUMAN	2	0	1	0.000	0.000	0.000	0.000	0.000	0.000	0.000	0.000	0.000	0.000	0.000	3.480					0
P28072	PSB6_HUMAN	2	0	1	0.000	0.000	0.000	0.000	0.000	0.000	0.000	0.000	0.000	0.000	0.000	3.498				yes	1
Q9Y2Q5	LTOR2_HUMAN	2	0	1	0.000	0.000	0.000	0.000	0.000	0.000	0.000	0.000	0.000	0.000	0.000	3.355					0
O76096	CYTF_HUMAN	2	0	1	0.000	0.000	0.000	0.000	0.000	0.000	0.000	0.000	0.000	0.000	0.000	3.485					0
P53621	COPA_HUMAN	2	0	1	0.000	0.000	0.000	0.000	0.000	0.000	0.000	0.000	0.000	0.000	0.000	3.804			yes	yes	2
P49913	CAMP_HUMAN	2	0	1	0.000	0.000	0.000	0.000	0.000	0.000	0.000	0.000	0.000	0.000	0.000	4.391	yes				1
Q9NRR3	C42S2_HUMAN	2	0	1	0.000	0.000	0.000	0.000	0.000	0.000	0.000	0.000	0.000	0.000	0.000	4.143					0
Q9NW15	ANO10_HUMAN	2	0	1	0.000	0.000	0.000	0.000	0.000	0.000	0.000	0.000	0.000	0.000	0.000	3.868					0
O95336	6PGL_HUMAN	2	0	1	0.000	0.000	0.000	0.000	0.000	0.000	0.000	0.000	0.000	0.000	0.000	3.686			yes		1
Q9UIB8	SLAF5_HUMAN	2	0	1	0.000	0.000	0.000	0.000	0.000	0.000	0.000	0.000	0.000	0.000	0.000	3.958					0

P14854	CX6B1_HUMAN	2	0	1	0.000	0.000	0.000	0.000	0.000	0.000	0.000	0.000	0.000	0.000	0.000	3.684					0
P09622	DLDH_HUMAN	2	0	1	0.000	0.000	0.000	0.000	0.000	0.000	0.000	0.000	0.000	0.000	4.233	0.000		yes	yes	yes	3
Q9Y6N5	SQRD_HUMAN	2	0	1	0.000	0.000	0.000	0.000	0.000	0.000	0.000	0.000	0.000	0.000	4.406	0.000				yes	1
P61803	DAD1_HUMAN	2	0	1	0.000	0.000	0.000	0.000	0.000	0.000	0.000	0.000	0.000	0.000	4.083	0.000				yes	1
P11279	LAMP1_HUMAN	2	0	1	0.000	0.000	0.000	0.000	0.000	0.000	0.000	0.000	0.000	0.000	5.579	0.000		yes		yes	2
P34897	GLYM_HUMAN	2	0	1	0.000	0.000	0.000	0.000	0.000	0.000	0.000	0.000	0.000	0.000	4.213	0.000				yes	1
P20073	ANXA7_HUMAN	2	0	1	0.000	0.000	0.000	0.000	0.000	0.000	0.000	0.000	0.000	0.000	4.312	0.000	yes		yes	yes	3
P49257	LMAN1_HUMAN	2	0	1	0.000	0.000	0.000	0.000	0.000	0.000	0.000	0.000	0.000	0.000	4.060	0.000				yes	1
P16401	H15_HUMAN	2	0	1	3.021	0.000	0.000	0.000	0.000	0.000	0.000	0.000	0.000	0.000	0.000	0.000				yes	1
P01833	PIGR_HUMAN	2	1	1	0.000	0.000	4.780	0.000	0.000	0.000	0.000	0.000	0.000	0.000	0.000	0.000		yes			1
P05388	RLA0_HUMAN	2	0	1	3.155	0.000	0.000	0.000	0.000	0.000	0.000	0.000	0.000	0.000	0.000	0.000				yes	1
Q5VSP4	LC1L1_HUMAN	2	0	1	4.194	0.000	0.000	0.000	0.000	0.000	0.000	0.000	0.000	0.000	0.000	0.000					0
P00451	FA8_HUMAN	2	0	1	0.000	0.000	3.277	0.000	0.000	0.000	0.000	0.000	0.000	0.000	0.000	0.000	yes				1
Q15517	CDSN_HUMAN	2	0	1	0.000	0.000	3.451	0.000	0.000	0.000	0.000	0.000	0.000	0.000	0.000	0.000		yes			1
P20160	CAP7_HUMAN	2	0	1	2.948	0.000	0.000	0.000	0.000	0.000	0.000	0.000	0.000	0.000	0.000	0.000	yes				1
P49908	SEPP1_HUMAN	2	0	1	0.000	0.000	3.113	0.000	0.000	0.000	0.000	0.000	0.000	0.000	0.000	0.000					0
Q9HCY8	S10AE_HUMAN	2	0	1	4.527	0.000	0.000	0.000	0.000	0.000	0.000	0.000	0.000	0.000	0.000	0.000					0
P04206	KV307_HUMAN	2	1	1	0.000	0.000	4.933	0.000	0.000	0.000	0.000	0.000	0.000	0.000	0.000	0.000					0
Q68DN1	CB016_HUMAN	2	0	1	0.000	0.000	2.874	0.000	0.000	0.000	0.000	0.000	0.000	0.000	0.000	0.000					0
Q8NCM8	DYHC2_HUMAN	2	0	1	0.000	0.000	2.832	0.000	0.000	0.000	0.000	0.000	0.000	0.000	0.000	0.000					0
Q13867	BLMH_HUMAN	2	0	1	0.000	0.000	0.000	0.000	0.000	0.000	0.000	0.000	4.182	0.000	0.000	0.000					0
P22531	SPR2E_HUMAN	2	0	1	0.000	0.000	0.000	0.000	0.000	0.000	0.000	0.000	5.326	0.000	0.000	0.000					0
O75874	IDHC_HUMAN	2	0	1	0.000	0.000	0.000	0.000	3.603	0.000	0.000	0.000	0.000	0.000	0.000	0.000			yes	yes	2
O15230	LAMA5_HUMAN	2	0	1	0.000	0.000	0.000	0.000	4.404	0.000	0.000	0.000	0.000	0.000	0.000	0.000					0
O00585	CCL21_HUMAN	2	0	1	0.000	0.000	0.000	0.000	4.325	0.000	0.000	0.000	0.000	0.000	0.000	0.000					0
Q86W10	CP4Z1_HUMAN	2	0	1	0.000	0.000	0.000	0.000	3.705	0.000	0.000	0.000	0.000	0.000	0.000	0.000					0
P60900	PSA6_HUMAN	2	0	1	0.000	0.000	0.000	0.000	0.000	0.000	0.000	0.000	0.000	3.905	0.000	0.000				yes	1
P68402	PA1B2_HUMAN	2	0	1	0.000	0.000	0.000	0.000	0.000	0.000	0.000	0.000	0.000	3.492	0.000	0.000					0
P08575	PTPRC_HUMAN	2	0	1	0.000	0.000	0.000	0.000	0.000	4.250	0.000	0.000	0.000	0.000	0.000	0.000	yes				1
P08571	CD14_HUMAN	2	0	1	0.000	0.000	0.000	0.000	0.000	3.550	0.000	0.000	0.000	0.000	0.000	0.000		yes			1
Q2LD37	K1109_HUMAN	2	0	1	0.000	0.000	0.000	0.000	0.000	3.035	0.000	0.000	0.000	0.000	0.000	0.000					0
P52943	CRIP2_HUMAN	2	0	1	0.000	0.000	0.000	0.000	0.000	3.577	0.000	0.000	0.000	0.000	0.000	0.000				yes	1
P50416	CPT1A_HUMAN	2	0	1	0.000	0.000	0.000	0.000	0.000	3.505	0.000	0.000	0.000	0.000	0.000	0.000				yes	1
P60033	CD81_HUMAN	2	0	1	0.000	0.000	0.000	0.000	0.000	3.550	0.000	0.000	0.000	0.000	0.000	0.000	yes			yes	2
Q2TV78	MSTP9_HUMAN	2	0	1	0.000	0.000	0.000	0.000	0.000	3.508	0.000	0.000	0.000	0.000	0.000	0.000					0
P04211	LV001_HUMAN	2	0	1	0.000	0.000	0.000	0.000	0.000	4.507	0.000	0.000	0.000	0.000	0.000	0.000					0
P47929	LEG7_HUMAN	2	1	1	0.000	0.000	0.000	0.000	0.000	5.098	0.000	0.000	0.000	0.000	0.000	0.000					0
P58107	EPIPL_HUMAN	2	0	1	0.000	0.000	0.000	0.000	0.000	3.559	0.000	0.000	0.000	0.000	0.000	0.000					0
Q6P5S2	CF058_HUMAN	2	0	1	0.000	0.000	0.000	0.000	0.000	3.301	0.000	0.000	0.000	0.000	0.000	0.000					0
Q96IU4	ABHEB_HUMAN	2	0	1	0.000	0.000	0.000	0.000	0.000	3.363	0.000	0.000	0.000	0.000	0.000	0.000					0
Q96DA0	ZG16B_HUMAN	2	0	1	0.000	0.000	0.000	0.000	0.000	3.885	0.000	0.000	0.000	0.000	0.000	0.000					0
Q9BXS5	AP1M1_HUMAN	2	0	1	0.000	0.000	0.000	0.000	0.000	3.264	0.000	0.000	0.000	0.000	0.000	0.000			yes		1
P12883	MYH7_HUMAN	2	0	1	0.000	0.000	0.000	0.000	0.000	0.000	4.489	0.000	0.000	0.000	0.000	0.000					0
Q13510	ASAH1_HUMAN	2	0	1	0.000	0.000	0.000	0.000	0.000	0.000	4.297	0.000	0.000	0.000	0.000	0.000					0
Q96IX5	USMG5_HUMAN	2	0	1	0.000	0.000	0.000	0.000	0.000	0.000	4.983	0.000	0.000	0.000	0.000	0.000		yes			1
O75352	MPU1_HUMAN	2	0	1	0.000	0.000	0.000	0.000	0.000	0.000	3.819	0.000	0.000	0.000	0.000	0.000				yes	1
P01600	KV108_HUMAN	2	0	1	0.000	0.000	0.000	0.000	0.000	0.000	4.781	0.000	0.000	0.000	0.000	0.000					0
P56385	ATP5I_HUMAN	2	0	1	0.000	0.000	0.000	0.000	0.000	0.000	4.046	0.000	0.000	0.000	0.000	0.000					0
Q99943	PLCA_HUMAN	2	0	1	0.000	0.000	0.000	0.000	0.000	0.000	3.526	0.000	0.000	0.000	0.000	0.000					0
P09382	LEG1_HUMAN	2	0	1	0.000	0.000	0.000	0.000	0.000	0.000	5.062	0.000	0.000	0.000	0.000	0.000				yes	1
Q9NS69	TOM22_HUMAN	2	0	1	0.000	0.000	0.000	0.000	0.000	0.000	4.646	0.000	0.000	0.000	0.000	0.000				yes	1
Q9H9B4	SFXN1_HUMAN	2	0	1	0.000	0.000	0.000	0.000	0.000	0.000	4.116	0.000	0.000	0.000	0.000	0.000				yes	1
P53634	CATC_HUMAN	2	0	1	0.000	0.000	0.000	0.000	0.000	0.000	4.355	0.000	0.000	0.000	0.000	0.000				yes	1
P36543	VATE1_HUMAN	2	0	1	0.000	4.846	0.000	0.000	0.000	0.000	0.000	0.000	0.000	0.000	0.000	0.000				yes	1
Q00765	REEP5_HUMAN	2	0	1	0.000	4.896	0.000	0.000	0.000	0.000	0.000	0.000	0.000	0.000	0.000	0.000					0
O60229	KALRN_HUMAN	2	0	1	0.000	4.005	0.000	0.000	0.000	0.000	0.000	0.000	0.000	0.000	0.000	0.000					0
P10599	THIO_HUMAN	2	0	1	0.000	4.600	0.000	0.000	0.000	0.000	0.000	0.000	0.000	0.000	0.000	0.000	yes	yes		yes	3
P62195	PRS8_HUMAN	2	0	1	0.000	4.112	0.000	0.000	0.000	0.000	0.000	0.000	0.000	0.000	0.000	0.000				yes	1
Q96RT1	LAP2_HUMAN	2	0	1	0.000	4.161	0.000	0.000	0.000	0.000	0.000	0.000	0.000	0.000	0.000	0.000					0
Q9NZ08	ERAP1_HUMAN	2	0	1	0.000	4.272	0.000	0.000	0.000	0.000	0.000	0.000	0.000	0.000	0.000	0.000				yes	1
Q9Y315	DEOC_HUMAN	2	0	1	0.000	4.231	0.000	0.000	0.000	0.000	0.000	0.000	0.000	0.000	0.000	0.000			yes		1
Q9UNS2	CSN3_HUMAN	2	0	1	0.000	4.696	0.000	0.000	0.000	0.000	0.000	0.000	0.000	0.000	0.000	0.000					0

P22307	NLTP_HUMAN	2	0	1	0.000	3.989	0.000	0.000	0.000	0.000	0.000	0.000	0.000	0.000	0.000	0.000				yes	1
Q9HC38	GLOD4_HUMAN	2	0	1	0.000	4.094	0.000	0.000	0.000	0.000	0.000	0.000	0.000	0.000	0.000	0.000					0
Q9P126	CLC1B_HUMAN	2	0	1	0.000	4.135	0.000	0.000	0.000	0.000	0.000	0.000	0.000	0.000	0.000	0.000					0
Q969H8	CS010_HUMAN	2	0	1	0.000	3.800	0.000	0.000	0.000	0.000	0.000	0.000	0.000	0.000	0.000	0.000				yes	1
P61421	VA0D1_HUMAN	2	0	1	0.000	4.202	0.000	0.000	0.000	0.000	0.000	0.000	0.000	0.000	0.000	0.000				yes	1
Q14773	ICAM4_HUMAN	2	0	1	0.000	3.977	0.000	0.000	0.000	0.000	0.000	0.000	0.000	0.000	0.000	0.000					0
O95721	SNP29_HUMAN	2	0	1	0.000	4.183	0.000	0.000	0.000	0.000	0.000	0.000	0.000	0.000	0.000	0.000					0
Q01433	AMPD2_HUMAN	2	0	1	0.000	4.035	0.000	0.000	0.000	0.000	0.000	0.000	0.000	0.000	0.000	0.000			yes		1
O75695	XRP2_HUMAN	2	0	1	0.000	3.882	0.000	0.000	0.000	0.000	0.000	0.000	0.000	0.000	0.000	0.000				yes	1
P28062	PSB8_HUMAN	2	0	1	0.000	3.930	0.000	0.000	0.000	0.000	0.000	0.000	0.000	0.000	0.000	0.000			yes		1
Q07960	RHG01_HUMAN	2	0	1	0.000	4.198	0.000	0.000	0.000	0.000	0.000	0.000	0.000	0.000	0.000	0.000			yes	yes	2
P50213	IDH3A_HUMAN	2	0	1	0.000	4.072	0.000	0.000	0.000	0.000	0.000	0.000	0.000	0.000	0.000	0.000				yes	1
P12830	CADH1_HUMAN	2	0	1	0.000	4.501	0.000	0.000	0.000	0.000	0.000	0.000	0.000	0.000	0.000	0.000					0
Q07812	BAX_HUMAN	2	0	1	0.000	4.046	0.000	0.000	0.000	0.000	0.000	0.000	0.000	0.000	0.000	0.000				yes	1
P62333	PRS10_HUMAN	2	0	1	0.000	4.135	0.000	0.000	0.000	0.000	0.000	0.000	0.000	0.000	0.000	0.000				yes	1
Q6NVY1	HIBCH_HUMAN	2	0	1	0.000	4.110	0.000	0.000	0.000	0.000	0.000	0.000	0.000	0.000	0.000	0.000					0
P19338	NUCL_HUMAN	2	0	1	0.000	0.000	0.000	3.771	0.000	0.000	0.000	0.000	0.000	0.000	0.000	0.000				yes	1
P38117	ETFB_HUMAN	2	0	1	0.000	0.000	0.000	3.665	0.000	0.000	0.000	0.000	0.000	0.000	0.000	0.000					0
P43034	LIS1_HUMAN	2	0	1	0.000	0.000	0.000	3.645	0.000	0.000	0.000	0.000	0.000	0.000	0.000	0.000			yes		1
Q9Y6E0	STK24_HUMAN	2	0	1	0.000	0.000	0.000	3.540	0.000	0.000	0.000	0.000	0.000	0.000	0.000	0.000					0
Q9BS26	ERP44_HUMAN	2	0	1	0.000	0.000	0.000	3.677	0.000	0.000	0.000	0.000	0.000	0.000	0.000	0.000				yes	1
P35998	PRS7_HUMAN	2	0	1	0.000	0.000	0.000	3.655	0.000	0.000	0.000	0.000	0.000	0.000	0.000	0.000			yes	yes	2
Q8IUI8	CRLF3_HUMAN	2	0	1	0.000	0.000	0.000	3.790	0.000	0.000	0.000	0.000	0.000	0.000	0.000	0.000			yes		1
P08263	GSTA1_HUMAN	2	0	1	0.000	0.000	0.000	0.000	0.000	0.000	0.000	0.000	0.000	0.000	0.000	3.382					0
P20851	C4BPB_HUMAN	2	0	1	0.000	0.000	0.000	0.000	0.000	0.000	3.819	0.000	0.000	0.000	0.000	0.000	yes				1
Q9H082	RB33B_HUMAN	2	1	1	0.000	0.000	0.000	0.000	0.000	0.000	5.826	0.000	0.000	0.000	0.000	0.000					0
Q04756	HGFA_HUMAN	2	0	1	0.000	4.264	0.000	0.000	0.000	0.000	0.000	0.000	0.000	0.000	0.000	0.000					0
P01780	HV319_HUMAN	2	0	1	0.000	0.000	0.000	3.668	0.000	0.000	0.000	0.000	0.000	0.000	0.000	0.000					0
P05787	K2C8_HUMAN	2	0	1	0.000	0.000	0.000	0.000	0.000	3.965	0.000	0.000	0.000	0.000	0.000	0.000					0
Q9NQ75	CASS4_HUMAN	3	0	1	0.000	0.000	0.000	0.000	0.000	0.000	0.000	4.659	0.000	0.000	0.000	0.000			yes		1
P48643	TCPE_HUMAN	3	1	1	0.000	0.000	0.000	0.000	0.000	0.000	0.000	5.793	0.000	0.000	0.000	0.000			yes	yes	2
Q9H2K8	TAOK3_HUMAN	3	0	1	0.000	0.000	0.000	0.000	0.000	0.000	0.000	4.529	0.000	0.000	0.000	0.000					0
Q9UL46	PSME2_HUMAN	3	0	1	0.000	0.000	0.000	0.000	0.000	0.000	0.000	4.998	0.000	0.000	0.000	0.000				yes	1
P33176	KINH_HUMAN	3	0	1	0.000	0.000	0.000	0.000	0.000	0.000	0.000	4.773	0.000	0.000	0.000	0.000			yes		1
Q16555	DPYL2_HUMAN	3	0	1	0.000	0.000	0.000	0.000	0.000	0.000	0.000	4.849	0.000	0.000	0.000	0.000				yes	1
Q9H3U1	UN45A_HUMAN	3	0	1	0.000	0.000	0.000	0.000	0.000	0.000	0.000	4.802	0.000	0.000	0.000	0.000					0
Q9Y3A6	TMED5_HUMAN	3	0	1	0.000	0.000	0.000	0.000	0.000	0.000	0.000	4.927	0.000	0.000	0.000	0.000					0
P38606	VATA_HUMAN	3	0	1	0.000	0.000	0.000	0.000	0.000	0.000	0.000	4.558	0.000	0.000	0.000	0.000			yes	yes	2
P30626	SORCN_HUMAN	3	0	1	0.000	0.000	0.000	0.000	0.000	0.000	0.000	4.539	0.000	0.000	0.000	0.000	yes				1
P30085	KCY_HUMAN	3	0	1	0.000	0.000	0.000	0.000	0.000	0.000	0.000	4.679	0.000	0.000	0.000	0.000			yes	yes	2
Q96CN7	ISOC1_HUMAN	3	0	1	0.000	0.000	0.000	0.000	0.000	0.000	0.000	5.022	0.000	0.000	0.000	0.000					0
Q9Y678	COPG_HUMAN	3	0	1	0.000	0.000	0.000	0.000	0.000	0.000	0.000	4.827	0.000	0.000	0.000	0.000			yes	yes	2
O14672	ADA10_HUMAN	3	0	1	0.000	0.000	0.000	0.000	0.000	0.000	0.000	4.885	0.000	0.000	0.000	0.000		yes	yes		2
A0FGR8	ESYT2_HUMAN	3	0	1	0.000	0.000	0.000	0.000	0.000	0.000	0.000	4.692	0.000	0.000	0.000	0.000					0
P11586	C1TC_HUMAN	3	0	1	0.000	0.000	0.000	0.000	0.000	0.000	0.000	5.159	0.000	0.000	0.000	0.000			yes		1
Q86WI1	PKHL1_HUMAN	3	0	1	0.000	0.000	0.000	0.000	0.000	0.000	0.000	0.000	0.000	0.000	0.000	4.743					0
O14974	MYPT1_HUMAN	3	0	1	0.000	0.000	0.000	0.000	0.000	0.000	0.000	0.000	0.000	0.000	0.000	4.314					0
Q8WWA0	ITLN1_HUMAN	3	1	1	5.539	0.000	0.000	0.000	0.000	0.000	0.000	0.000	0.000	0.000	0.000	0.000					0
Q9NP58	ABCB6_HUMAN	3	0	1	0.000	0.000	3.695	0.000	0.000	0.000	0.000	0.000	0.000	0.000	0.000	0.000					0
P31151	S10A7_HUMAN	3	0	1	4.782	0.000	0.000	0.000	0.000	0.000	0.000	0.000	0.000	0.000	0.000	0.000					0
P05387	RLA2_HUMAN	3	1	1	0.000	0.000	0.000	0.000	0.000	5.347	0.000	0.000	0.000	0.000	0.000	0.000				yes	1
O75556	SG2A1_HUMAN	3	1	1	0.000	0.000	0.000	0.000	0.000	6.369	0.000	0.000	0.000	0.000	0.000	0.000					0
Q53RT3	APRV1_HUMAN	3	1	1	0.000	0.000	0.000	0.000	0.000	4.967	0.000	0.000	0.000	0.000	0.000	0.000					0
Q9NP55	BPIA1_HUMAN	3	0	1	0.000	0.000	0.000	0.000	0.000	4.734	0.000	0.000	0.000	0.000	0.000	0.000					0
P05154	IPSP_HUMAN	3	0	1	0.000	0.000	0.000	0.000	0.000	4.165	0.000	0.000	0.000	0.000	0.000	0.000					0
Q9C075	K1C23_HUMAN	3	0	1	0.000	0.000	0.000	0.000	0.000	3.811	0.000	0.000	0.000	0.000	0.000	0.000					0
Q9GZZ8	LACRT_HUMAN	3	1	1	0.000	0.000	0.000	0.000	0.000	5.593	0.000	0.000	0.000	0.000	0.000	0.000					0
Q9BSF0	CB088_HUMAN	3	0	1	0.000	0.000	0.000	0.000	0.000	0.000	4.924	0.000	0.000	0.000	0.000	0.000					0
O60234	GMFG_HUMAN	3	0	1	0.000	5.148	0.000	0.000	0.000	0.000	0.000	0.000	0.000	0.000	0.000	0.000			yes		1
O43396	TXNL1_HUMAN	3	0	1	0.000	4.739	0.000	0.000	0.000	0.000	0.000	0.000	0.000	0.000	0.000	0.000			yes		1
O60814	H2B1K_HUMAN	3	1	1	0.000	5.751	0.000	0.000	0.000	0.000	0.000	0.000	0.000	0.000	0.000	0.000					0
Q13609	DNSL3_HUMAN	3	0	1	0.000	4.711	0.000	0.000	0.000	0.000	0.000	0.000	0.000	0.000	0.000	0.000					0

Q8IZ83	A16A1_HUMAN	3	0	1	0.000	0.000	0.000	4.381	0.000	0.000	0.000	0.000	0.000	0.000	0.000	0.000					0
P98160	PGBM_HUMAN	3	0	1	0.000	0.000	0.000	0.000	0.000	0.000	4.441	0.000	0.000	0.000	0.000	0.000		yes		yes	2
P11226	MBL2_HUMAN	3	0	1	4.723	0.000	0.000	0.000	0.000	0.000	0.000	0.000	0.000	0.000	0.000	0.000					0
P49368	TCPG_HUMAN	4	0	1	0.000	0.000	0.000	0.000	0.000	0.000	0.000	5.465	0.000	0.000	0.000	0.000			yes	yes	2
P11150	LIPC_HUMAN	4	1	1	0.000	0.000	0.000	0.000	6.996	0.000	0.000	0.000	0.000	0.000	0.000	0.000					0
P06702	S10A9_HUMAN	4	1	1	0.000	0.000	0.000	0.000	0.000	7.414	0.000	0.000	0.000	0.000	0.000	0.000	yes	yes			2
Q16378	PROL4_HUMAN	4	1	1	0.000	0.000	0.000	0.000	0.000	5.813	0.000	0.000	0.000	0.000	0.000	0.000					0
Q01469	FABP5_HUMAN	4	1	1	0.000	0.000	0.000	0.000	0.000	6.518	0.000	0.000	0.000	0.000	0.000	0.000					0
P30838	AL3A1_HUMAN	4	0	1	0.000	0.000	0.000	0.000	0.000	4.784	0.000	0.000	0.000	0.000	0.000	0.000					0
P14324	FPPS_HUMAN	4	0	1	0.000	4.978	0.000	0.000	0.000	0.000	0.000	0.000	0.000	0.000	0.000	0.000					0
Q86VP6	CAND1_HUMAN	5	1	1	0.000	0.000	0.000	0.000	0.000	0.000	0.000	5.647	0.000	0.000	0.000	0.000				yes	1
Q9NZT1	CALL5_HUMAN	5	1	1	0.000	0.000	0.000	0.000	0.000	7.528	0.000	0.000	0.000	0.000	0.000	0.000		yes			1
O00159	MYO1C_HUMAN	5	0	1	0.000	5.579	0.000	0.000	0.000	0.000	0.000	0.000	0.000	0.000	0.000	0.000				yes	1
Q8IUX7	AEBP1_HUMAN	6	1	1	0.000	0.000	0.000	0.000	0.000	0.000	0.000	0.000	5.569	0.000	0.000	0.000					0
P02808	STAT_HUMAN	6	1	1	0.000	0.000	0.000	0.000	0.000	6.178	0.000	0.000	0.000	0.000	0.000	0.000					0
Q15149	PLEC_HUMAN	16	1	1	0.000	0.000	0.000	0.000	0.000	0.000	0.000	0.000	0.000	0.000	7.215	0.000				yes	1
Q8TDL5	BPIB1_HUMAN	20	1	1	0.000	0.000	0.000	0.000	0.000	7.612	0.000	0.000	0.000	0.000	0.000	0.000		yes			1
